# Making Home Sweet and Sturdy: *Toxoplasma gondii* ppGalNAc-Ts Glycosylate in Hierarchical Order and Confer Cyst Wall Rigidity

**DOI:** 10.1128/mBio.02048-16

**Published:** 2017-01-10

**Authors:** Tadakimi Tomita, Tatsuki Sugi, Rama Yakubu, Vincent Tu, Yanfen Ma, Louis M. Weiss

**Affiliations:** a Department of Pathology, Albert Einstein College of Medicine, Bronx, New York, USA; b Department of Medicine, Albert Einstein College of Medicine, Bronx, New York, USA; Washington University School of Medicine

## Abstract

The protozoan intracellular parasite *Toxoplasma gondii* forms latent cysts in the central nervous system (CNS) and persists for the lifetime of the host. This cyst is cloaked with a glycosylated structure called the cyst wall. Previously, we demonstrated that a mucin-like glycoprotein, CST1, localizes to the cyst wall and confers structural rigidity on brain cysts in a mucin-like domain-dependent manner. The mucin-like domain of CST1 is composed of 20 units of threonine-rich tandem repeats that are *O-*GalNAc glycosylated. A family of enzymes termed polypeptide *N*-acetylgalactosaminyltransferases (ppGalNAc-Ts) initiates *O*-GalNAc glycosylation. To identify which isoforms of ppGalNAc-Ts are responsible for the glycosylation of the CST1 mucin-like domain and to evaluate the function of each ppGalNAc-T in the overall glycosylation of the cyst wall, all five ppGalNAc-T isoforms were deleted individually from the *T. gondii* genome. The ppGalNAc-T2 and -T3 deletion mutants produced various glycosylation defects on the cyst wall, implying that many cyst wall glycoproteins are glycosylated by T2 and T3. Both T2 and T3 glycosylate the CST1 mucin-like domain, and this glycosylation is necessary for CST1 to confer structural rigidity on the cyst wall. We established that T2 is required for the initial glycosylation of the mucin-like domain and that T3 is responsible for the sequential glycosylation on neighboring acceptor sites, demonstrating hierarchical glycosylation by two distinct initiating and filling-in ppGalNAc-Ts in an intact organism.

## IMPORTANCE

*Toxoplasma gondii* is an obligate intracellular parasite that infects a third of the world’s population. It can cause severe congenital disease and devastating encephalitis in immunocompromised individuals. We identified two glycosyltransferases, ppGalNAc-T2 and -T3, which are responsible for glycosylating cyst wall proteins in a hierarchical fashion. This glycosylation confers structural rigidity on the brain cyst. Our studies provide new insights into the mechanisms of *O-*GalNAc glycosylation in *T. gondii*.

## INTRODUCTION

*Toxoplasma gondii* is an intracellular protozoan parasite that infects up to a third of the world’s population (1). The asexual cycle of *T. gondii* has two developmental stages: a rapidly replicating form called the tachyzoite and a slow-growing form called the bradyzoite that forms tissue cysts (2). Development of cysts (modified parasitophorous vacuoles containing bradyzoites) can occur in any warm-blooded animal. Ingestion of these tissue cysts in undercooked meat is an important source of *T. gondii* infection. The other mechanism of transmission is ingestion of oocysts (the sexual stage of this parasite) that are found in cat feces and which can contaminate food or water. The persistence and reactivation of bradyzoites in tissue cysts are a critical factor in the progression of disease in patients with congenital infection as well as in patients who are immunocompromised (3). Disease from chronic toxoplasmosis is thought to occur as a consequence of recrudescence of infection from tissue cysts residing in muscle, eye, or the central nervous system (3–5). Due to its central importance in initiating infection and disease pathogenesis, the biology of the bradyzoite stage and how tissue cysts are formed are an active area of research.

Tissue cysts reside in a modified parasitophorous vacuole termed the cyst wall. This 250- to 500-nm-thick structure consists of the limiting membrane, a compact outer layer, and an inner sponge-like layer (6). The cyst wall is modified with various glycans, and this is most likely due to posttranslational modifications occurring on various cyst wall proteins. Glycosylated cyst wall components are stained by periodic acid-Schiff (PAS) stain, which binds to carbohydrates, as well as various lectins, including *Dolichos biflorus* lectin (DBA), which binds to GalNAcα1-3GalNAc, and succinylated wheat germ lectin (s-WGA), which binds to terminal GlcNAc (7). Recently, characterized cyst wall proteins CST1 (TGME49_264660) and *T. gondii* proteophosphoglycan 1 (TgPPG1; TGME49_297520) have both been demonstrated to be glycosylated proteins (8, 9). Previously, our laboratory examined the *T. gondii* glycoproteome by using lectin affinity purification coupled with mass spectrometry methods (10). This experimental data set contained many secreted glycoproteins, including proteins that are now known to be located in the cyst wall, such as various dense granule proteins (GRA2, -3, and -6) and cyst matrix proteins (GRA7 and MAG1).

*O-*GalNAc glycosylation is a common posttranslational modification in many eukaryotes that is found on various secreted and membrane-associated proteins. These *O-*GalNAc posttranslational modifications often play an important biological role. For example, *O-*GalNAc glycosylation of proapoptotic receptors DR4/DR5 (11), fibroblast growth factor FGF23 (12), or megakaryocyte/platelet glycoprotein GPIbα (13) is critical for their functions, although the exact mechanisms by which this posttranslational modification alters the functions of these various proteins are not completely understood. Mucins are an important class of glycoproteins characterized by a posttranslational modification with hundreds of GalNAc *O-*glycosylations that can represent ~80% of the molecular mass of these proteins. A key characteristic of mucin proteins is the presence of a mucin-like domain composed of unique serine-, threonine-, and proline-rich tandem repeats. Mammalian mucins play a central role in formation of glycocalyx on vascular endothelial tissues and mucus layers in the digestive tract. Some transmembrane mucins have cytoplasmic domains involved in signal transduction and have been implicated in tumor growth and motility (14, 15). Mucin-like domains are also present in yeast osmotic sensors such as Hkr1 and Msb2 (16, 17). CST1, which is found in the cyst wall of *T. gondii*, contains a mucin-like domain, and we have previously demonstrated that this domain is essential for cyst stability and that the absence of this domain alters the ultrastructure of the cyst wall (2). The mucin domain of CST1 is, therefore, a critical component of the cyst wall that is involved in essential functions of this structure.

*O-*GalNAc glycosylation is initiated by the family of evolutionarily conserved enzymes termed polypeptide *N*-α-acetylgalactosaminyltransferases (ppGalNAc-Ts; EC 2.4.1.41). These enzymes (ppGalNAc-Ts) transfer GalNAc from UDP-GalNAc to the hydroxyl group of serine or threonine of polypeptide chains to form GalNAcα1-*O-*serine/threonine. In the *T. gondii* genome, there are five putative isoforms of ppGalNAc-T (T1, TGME49_259530; T2, TGME49_258770; T3, TGME49_318730; T4, TGME49_256080; and T5, TGME49_278518 [ToxoDB]). All of the *T. gondii* ppGalNAc-Ts have conserved glycosyltransferase domains that contain a GT-A motif and a DxH Mn^2+^ ion binding motif (18). Based on stage-specific gene expression data available at ToxoDB.org, *T. gondii* ppGalNAc-T1, -T2, and -T3 are constitutively expressed in both tachyzoite and bradyzoite stages, while the mRNA of T4 is exclusively expressed at the cat enteroepithelial stage and T5 mRNA is expressed at the cat enteroepithelial stage and oocyst stage (19–22) (see Fig. S1 in the supplemental material).

10.1128/mBio.02048-16.1 FIG S1  Expression levels of ppGalNAc-T isoforms determined by RNA-seq data. The relative gene expression levels of the five ppGalNAc-T isoforms identified in the genome are compared across the various differentiation states of *T. gondii* (e.g., tachyzoite, bradyzoite, sporozoite, and merozoite life cycle stages). Of these genes, ppGalNAc-T1, -T2, and -T3 are constitutively expressed; T4 expression is present only in the cat enterocyte stage (merozoite) exclusively; and T5 expression is highly upregulated in the oocyst stage and also present in the cat stage. All expression values were taken from the publicly available RNA-seq data in ToxoDB deposited by the previous studies (19–22). Download FIG S1, TIF file, 12.9 MB. Copyright © 2017 Tomita et al. 2017 Tomita et al. This content is distributed under the terms of the Creative Commons Attribution 4.0 International license.

The *T. gondii* ppGalNAc-T1, -T2, and -T3 proteins have prototypical ppGalNAc-T structural features (23). They consist of a short N-terminal cytoplasmic tail, a single transmembrane domain, a stem region, a conserved catalytic domain, and a C-terminal ricin-like lectin domain. *T. gondii* ppGalNAc-T4 is unusual in that it lacks the C-terminal ricin-like lectin domain, which is similar to the structure of human ppGalNAc-T20, which lacks catalytic activity in cell-free peptide GalNAc transferase assays (24).

Of the five putative *T. gondii* ppGalNAc-Ts, ppGalNAc-T1, -T2, and -T3 have been previously cloned and expressed in eukaryotic systems (25–27). Purified ppGalNAc-T1 and -T3 were shown to have transferase activity for GalNAc to preglycosylated acceptor peptides (MUC5AC-3,13 and EA2-7) but no activity to nonglycosylated naked acceptor peptides (MUC5AC and EA2) (25–27). A requirement for prior *O-*GalNAc glycosylation on the peptides adjacent to the acceptor residue would not be unique to *T. gondii* ppGalNAc-T1 and -T3, as this type of sequential activity has been documented *in vitro* using human ppGalNAc-T7 and -T10, which have minimal transferase activity on nonglycosylated peptides but highly enhanced transferase activity on preglycosylated acceptor peptides (28, 29). Demonstration of *in vitro* or *in vivo T. gondii* ppGalNAc-T2 transferase activity has not been previously reported (25–27).

It is likely that *T. gondii* ppGalNAc transferases play critical roles in the biology of this pathogenic organism. Previously, our group demonstrated that the mucin-like glycoprotein CST1 forms the cyst wall layer and confers cyst wall structural rigidity as well as facilitating persistence of cysts in mouse brain during chronic infection (9). These functions of CST1 were shown to be dependent on the presence of a C-terminal 260-amino-acid-long mucin-like domain that contains threonine-rich tandem repeats which are probably posttranslationally modified by *O-*GalNAc glycosylation. The lectin DBA, which recognizes the GalNAcα1-3GalNAc linkage (30, 31), binds to this mucin-like domain of the CST1, demonstrating that glycosylation is present on this domain.

The ppGalNAc-Ts that are responsible for glycosylation of this mucin-like domain have not been determined. Given that ppGalNAc-T1, -T2, and -T3 are the only ppGalNAc-Ts that are expressed in the tachyzoite and bradyzoite stages, we hypothesized that T2 is the “initiating transferase” that transfers GalNAc to unglycosylated peptides and that T1 and T3 are the “filling-in transferases” that recognize *O-*GalNAc and transfer additional GalNAc to the adjacent acceptor sites, resulting in a densely glycosylated mucin-like domain. Utilizing ppGalNAc-T-knockout *T. gondii* strains, we demonstrate that hierarchical *O*-GalNAc glycosylation occurs with the initiator ppGalNAc-T2 and filling-in ppGalNAc-T3 glycosylating the mucin-like domain of CST1 and that this hierarchical glycosylation is critical for the ability of CST1 to generate a rigid cyst wall.

## RESULTS

### Generation of GalNAc-T mutants.

Previous studies have demonstrated that *T. gondii* ppGalNAc-T1 and -T3 are catalytically active on synthesized short glycopeptides using an *in vitro* cell-free system (18, 25, 26). To understand the biological functions of these ppGalNAc-Ts, it is critical to investigate their role in the context of the whole organism where the natural target proteins exist. To this end, deletion mutant strains of *T. gondii* were developed for all five transferases, ppGalNAc-T1 to ppGalNAc-T5 (Δ*t1* to Δ*t5*, respectively; see Fig. S2 in the supplemental material). The deletion mutants were generated using double-homologous recombination in the Pru Δ*ku80 T. gondii* strain (32) utilizing previously described techniques (9). All of these deletion mutants were viable *in vitro* under both bradyzoite and tachyzoite culture conditions, indicating that single gene deletions of each ppGalNAc-T are neither lethal to the parasite nor essential for differentiation *in vitro* (data not shown). As phenotypes were seen for both the ppGalNAc-T2 and -T3 gene deletions (see the results below), complementation of the Δ*t3* mutant (Δ*t3*::*t3*) was performed at the uracil phosphoribosyltransferase (UPRT) locus using the 1.5-kb endogenous putative promoter to maintain the native expression pattern of T3. Complementation of the Δ*t2* mutant (Δ*t2*::*t2*) was performed at the endogenous ppGalNAc-T2 locus (using a pyrimethamine-resistant dihydrofolate reductase [DHFR] selectable marker), as complementation of the Δ*t2* mutant at the UPRT locus was not successful.

10.1128/mBio.02048-16.2 FIG S2  Generation of ppGalNAc-T deletion mutants. These are the diagnostic PCRs of *T. gondii* genomic DNA separated by agarose gel electrophoresis demonstrating the successful generation of the various ppGalNAc-T null mutants. The upper left gel illustrates the integration of the *Δt1* fragment into the T1 locus. The upper right is the detection of the T1 gene, indicating that the T1 gene is deleted. The middle left gel illustrates the integration of the *Δt2* fragment into the T2 locus. T2 complementation was done at the T2 locus, resulting in the lack of the *Δt2* fragment in the *Δt2*::*t2* mutant. The middle center gel shows detection of the T2 locus. This primer set is specific to the T2 genomic DNA of T2 and will not amplify the T2 cDNA. The middle right gel demonstrates detection of the T2 cDNA copy, indicating complementation of the *Δt2* strain. The lower left gel illustrates detection of the *Δt3* fragment at the T3 locus. The lower center gel demonstrates detection of T3. The lower right gel demonstrates detection of the T3 cDNA copy in the complemented strain. Download FIG S2, TIF file, 47.2 MB. Copyright © 2017 Tomita et al. 2017 Tomita et al. This content is distributed under the terms of the Creative Commons Attribution 4.0 International license.

### Lectin profiling of ppGalNAc-*Δt1*, -*Δt2*, and -*Δt3* parasites.

All previously described *O-*GalNAc glycans require a ppGalNAc-T for their initial glycosylation step. Therefore, the deletion of any of the identified *T. gondii* ppGalNAc-T genes could potentially disrupt the parasite glycoproteome. However, as described in mammalian studies, ppGalNAc-Ts have significant overlaps of substrate specificity among the multiple isoforms that have been identified, and this has limited the identification of the key ppGalNAc-Ts involved in this initial glycosylation in many eukaryotic systems (33–35). To investigate how the absence of each *T. gondii* ppGalNAc-T gene influences the overall glycosylation of parasite proteins and to confirm that deletion of the various ppGalNAc-T genes altered glycosylation in *T. gondii*, we employed various sugar-binding lectins (see Table S1 in the supplemental material) that bind to specific carbohydrate moieties of glycoproteins. We surveyed the potential substrates of ppGalNAc-Ts by comparing the glycoprotein bands probed with the various lectins between the wild type and deletion mutants. We reasoned that the absence of a specific ppGalNAc-T would result in either disappearance of a band or a reduction in the molecular weight of a band due to the loss of *O-*GalNAc glycosylation.

10.1128/mBio.02048-16.6 TABLE S1  List of lectins used for lectin survey. This is the list of lectins used for the lectin overlay and immunocytochemistry assays. The glycan specificities described in this file are from glycoarray data at the Consortium of Functional Glycomics gateway database (http://www.functionalglycomics.org). Download TABLE S1, DOCX file, 0.01 MB. Copyright © 2017 Tomita et al. 2017 Tomita et al. This content is distributed under the terms of the Creative Commons Attribution 4.0 International license.

**FIG 1:**
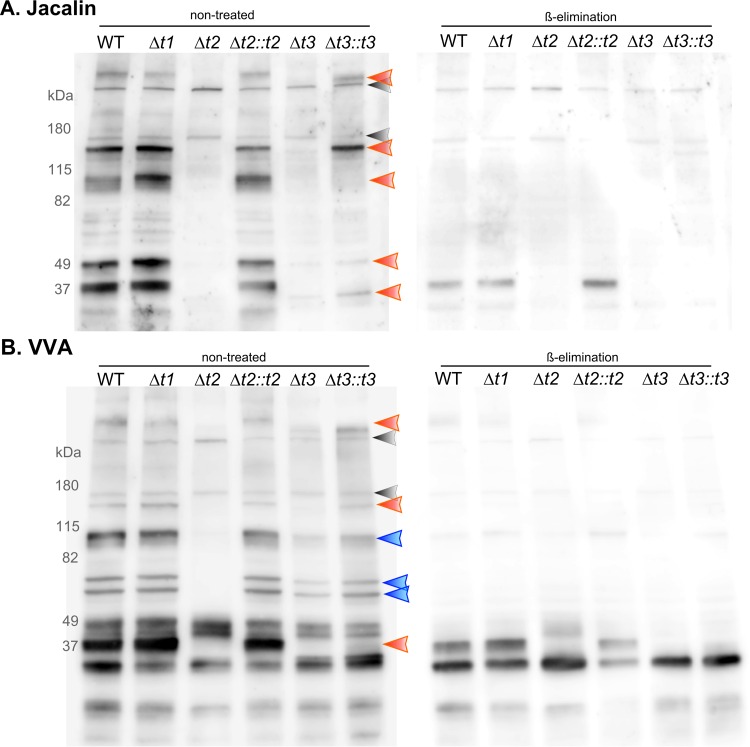
Lectin profiling of ppGalNAc-T-knockout strains. Purified lysates from *T. gondii* strains were separated by SDS-PAGE and transferred onto a PVDF membrane which was then incubated with either biotin-conjugated jacalin (A) or VVA (B). The orange arrowheads indicate the bands missing in both ppGalNAc-Δ*t2* and -Δ*t3* strains. The blue arrowheads indicate the bands missing in ppGalNAc-Δ*t2* but not in -Δ*t3*. The gray arrowheads indicate the nonspecific endogenous biotinylated proteins (streptavidin was used for detection). This blot demonstrates that multiple *T. gondii* glycoproteins require ppGalNAc-T2 and -T3 glycosyltransferase activities. The duplicate blots on the right were subjected to β-elimination and demonstrate that the identified bands are due to O-linked glycosylation. This figure demonstrates *T. gondii* cultivated under tachyzoite (pH 7.1 with 5% CO_2_) culture conditions, and Fig. S3 in the supplemental material demonstrates *T. gondii* cultivated under bradyzoite culture conditions (pH 8.1 with 0.1% CO_2_). WT, wild type.

Parasite lysates from our wild-type and knockout strains were separated by SDS-PAGE, transferred onto a polyvinylidene difluoride (PVDF) membrane, and probed with the following lectins: jacalin (*Artocarpus integrifolia* lectin), VVA (*Vicia villosa* lectin), HPA (*Helix pomatia* lectin), DBA (*Dolichos biflorus* lectin), concanavalin A (ConA) (*Canavalia ensiformis* lectin), s-WGA (succinylated wheat germ lectin), SNA-I (*Sambucus nigra* lectin I), and GSL-1 (*Griffonia simplicifolia* lectin I). Jacalin labeling identified five specific glycoprotein bands at 37, 49, 95, 150, and >180 kDa that are present in the wild type but absent in the ppGalNAc-Δ*t2* and -Δ*t3* parasites (Fig. 1A, orange arrowheads). Complementation of T2 restored all of these lectin-binding bands, and complementation of T3 restored all the bands with the exception of the band at 95 kDa. Similarly, VVA labeling identified specific glycoprotein bands at 37, 150, and >180 kDa (Fig. 1B) that are absent in both Δ*t2* and Δ*t3* (Fig. 1B, orange arrowheads). However, the 60-, 70-, and 95-kDa bands that are absent in ppGalNAc-*Δt2* are still present in Δ*t3* (blue arrows). Complementation of T2 restored all of these lectin-binding bands, and complementation of T3 restored all of these bands except the 37-kDa band. β-elimination confirmed that the bands recognized by these lectins were most likely *O*-glycosylated proteins (Fig. 1A and B). Overall, these data suggest that the formation of these lectin-binding glycoepitopes requires either ppGalNAc-T2 alone (blue arrowhead) or both T2 and T3 (orange arrowheads).

Jacalin has binding specificity to α-linked GalNAc (GalNAcα1-Ser/Thr) with the C_6_-OH free from substitution such as *O-*GalNAc glycan core 1 (Galβ1-3GalNAcα1-Ser/Thr) and core 3 (GlcNAcβ1-3GalNAcα1-Ser/Thr) (36). VVA binds to both terminal GalNAc and peptide-bound GalNAc, preferably GalNAc on Thr residues (30, 37). The lectin overlay data demonstrated that even though jacalin and VVA have similar specificities toward the GalNAc-containing glycans, jacalin and VVA have distinct band patterns and probably bind to overlapping subsets of *T. gondii* glycoproteins. These data also indicated that ppGalNAc-T2 and -T3 are functional glycosyltransferases in *T. gondii*, similarly to what is known about other catalytically verified eukaryotic ppGalNAc-Ts which have been clearly demonstrated to transfer GalNAc to (glyco-)polypeptides. Other lectins that bind N-linked glycans, such as ConA, did not display any visible differential glycoprotein band patterns on examination of lectin blots of wild type and transferase deletion mutants (data not shown), consistent with a role for ppGalNAc-Ts in the posttranslational modification of *O-*GalNAc glycosylation but not N-glycosylation.

Using this lectin overlay approach, we could not appreciate a difference in the binding patterns of parasites purified from cultures grown under either tachyzoite or bradyzoite conditions (see Fig. S3A and B in the supplemental material). One caveat is that parasites were purified by filtration with a 3-µm-pore membrane after the mechanical lysis of host cells, and thus, this experiment does not address changes in proteins found in the matrix of the parasitophorous vacuole, those on the parasitophorous vacuole membrane (including the majority of cyst wall glycoprotein CST1, which is known to bind to DBA lectin), and those parasite proteins that are injected into the host. Purification of the parasites was, however, necessary to reduce the overwhelming background of host cell glycoproteins that bind to lectins that would result from probing infected host cell lysates rather than purified parasites.

10.1128/mBio.02048-16.3 FIG S3  Comparison of jacalin and VVA binding proteins between tachyzoite stage and bradyzoite stage. Purified lysates from *T. gondii* strains cultivated under bradyzoite (pH 8.1 with 0.1% CO_2_) or tachyzoite (pH 7.1 with 5% CO_2_) culture conditions were separated by SDS-PAGE and then transferred onto nitrocellulose membranes. The membrane was then incubated with either biotin-conjugated jacalin (A) or VVA (B). This blot demonstrates that multiple *T. gondii* glycoproteins are similarly expressed between the bradyzoite and tachyzoite stages of this parasite. Download FIG S3, TIF file, 10.4 MB. Copyright © 2017 Tomita et al. 2017 Tomita et al. This content is distributed under the terms of the Creative Commons Attribution 4.0 International license.

**FIG 2:**
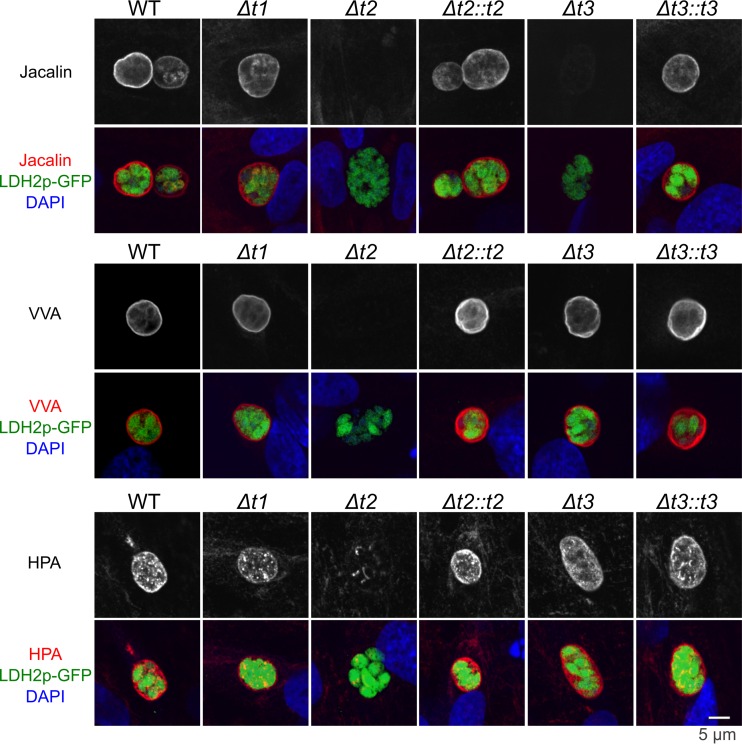
Binding of lectins to the cyst wall of ppGalNAc-T-knockout strains. Human foreskin fibroblasts (HFF) infected with wild-type and ppGalNAc-T deletion mutant *T. gondii* were cultured in pH 8.1 medium, facilitating the formation of *in vitro* cysts. Infected HFF cells were stained with jacalin (red, upper row), VVA (red, middle row), or HPA (red, bottom row) as well as the green fluorescent protein (green) driven by bradyzoite-specific promoter LDH2 and DAPI (blue). The Δ*t2* parasite lacks jacalin, VVA, and HPA cyst wall staining, while the *Δt3* parasite lacks jacalin staining only. This suggests that the various lectin-binding glycoproteins on the cyst wall are posttranslationally modified by ppGalNAc-T2 and -T3 glycosyltransferases.

Since the parasite purification step removed the majority of parasitophorous vacuole-localized proteins, we assessed the glycosylation status of the parasitophorous vacuole of the ppGalNAc-T mutants by staining *in vitro* cysts with the above mentioned lectins. Figure 2 illustrates human foreskin fibroblasts (HFF; obtained from ATCC) infected with *T. gondii* probed with jacalin, VVA, and HPA lectins. Jacalin cyst wall staining is absent in Δ*t2* and Δ*t3* parasites. In contrast, VVA cyst wall staining is absent only in Δ*t2* parasites. While HPA cyst wall staining is absent in Δ*t2* parasites, the punctate cytosolic HPA staining remained intact. These defects in lectin staining of the cyst wall in the ppGalNAc-T deletion mutants indicate that there are various cyst wall-associated glycoproteins that require ppGalNAc-T2 and -T3 activity for posttranslational *O*-GalNAc glycosylation.

To identify some of the potential glycoproteins which were likely to have been modified by ppGalNAc-T2, we employed the two lectins that had differential binding patterns in lectin overlay, namely, jacalin and VVA, for glycoprotein affinity purification. Comparison of lectin overlay and immunofluorescence assay (IFA) suggested that ppGalNAc-T2 activity was required for ppGalNAc-T3 activity: more bands were absent in ppGalNAc-Δ*t2* lectin overlay and the major bands absent in ppGalNAc-Δ*t3* were absent in ppGalNAc-Δ*t2* (Fig. 1A and B). Therefore, we decided to use ppGalNAc-Δ*t2* parasites for the identification of candidate substrates. Briefly, proteins from wild-type and *Δt2* parasite lysates were affinity purified with either jacalin- or VVA-conjugated beads and eluted proteins were separated by SDS-PAGE and analyzed by mass spectrometry. In the jacalin-purified material, we identified 602 *T. gondii* proteins (false discovery rate [FDR], 1.0%). Figure S4A in the supplemental material is a Venn diagram representing the binary overlaps of 602 jacalin-isolated proteins from wild-type, *Δt2*, and *Δt2*::*t2* parasites. Surprisingly, 51% of proteins fell into an intersection between all three genotypes. This could be due to the relatively low affinity of lectin-binding (*K*_*d*_ [dissociation constant], ~10^−5^ M for jacalin [38]), resulting in the inclusion of nonspecific abundant proteins. In this case, the use of binary exclusion criteria generates many false negatives (i.e., ppGalNAc-T2 substrate proteins that are still present in the ppGalNAc-Δ*t2* sample due to abundance). Therefore, to identify highly probable ppGalNAc-T2 substrate candidates, the semiquantitative criteria that proteins have more than 5 peptides mapped in wild type and more than a 2.5-fold-increased detection in wild type over the *Δt2* mutant were used to select potential ppGalNAc-T2 substrates. In addition, nuclear and nonsecreted proteins (which do not transverse the endoplasmic reticulum [ER]/Golgi complex) were removed from these lists. Table 1 provides the list of proteins identified with jacalin pulldown that meet these criteria. In the jacalin pulldown assay, the protein with the highest number of peptides meeting these criteria was CST1. These data strongly suggest that ppGalNAc-T2 modifies CST1 with GalNAc and are consistent with previous data showing that CST1 is a glycoprotein (8, 9). The jacalin lectin overlay experiment demonstrated five prominent glycoprotein bands at 37, 49, 95, 150, and >180 kDa (Fig. 1A) that were absent in *Δt2* parasite lysate. Proteomic analysis of the corresponding gel band at 37 kDa showed that it contained dense granular proteins GRA3 and GRA7. The band at 49 kDa contained a lysine decarboxylase family protein (TGME49_236570). The 95-kDa band contained the perforin-like protein 1 (PLP1). The 150-kDa band contained a hypothetical protein that is rich in serine (TGME49_230940). The high-molecular-weight band contained CST1, vacuolar protein sorting 13 (VPS13)-like protein (TGME49_291180), Sec7 domain-containing protein (TGME49_232190), and a large hypothetical protein with numerous serine-rich tandem repeats (TGME49_287480).

10.1128/mBio.02048-16.4 FIG S4  Predicted *O-*GalNAc sites in ppGalNAc-T2 substrate candidates compared with those found in the entire *T. gondii* proteome. Venn diagrams of binary overlaps for proteins identified with each genotype that were affinity purified with either jacalin (A) or VVA (B) or both jacalin and VVA (C). Panel D is a kernel density graph of the distribution of the number of predicted *O-*GalNAc glycosylation sites per protein for ppGalNAc-T2-specific substrate candidates identified by capture with jacalin (green) or VVA (blue) or found in the entire *T. gondii* proteome (red). The distribution of the number of the glycosylation sites was statistically significant between jacalin-captured candidates and the entire proteome (*, Kolmogorov-Smirnov test, *P* < 0.05). Download FIG S4, TIF file, 5.5 MB. Copyright © 2017 Tomita et al. 2017 Tomita et al. This content is distributed under the terms of the Creative Commons Attribution 4.0 International license.

**TABLE 1:** List of ppGalNAc-T2 substrate candidate proteins identified from the jacalin affinity purification^a^

Identified protein	Accession no.	Predicted *O*-GalNAc	Peptide count		
			WT	*Δt2*	WT/Δ*t2*
CST1	TGME49_264660	197	96	7	13.71
Hypothetical	TGME49_313270	67	42	17	2.55
Hypothetical	TGME49_230940	265	39	3.5	11.14
MIF4G domain containing	TGME49_269180	153	31	7.5	4.07
Hypothetical	TGME49_228120	117	25	5	4.90
GYF domain containing	TGME49_204160	37	14	5.5	2.55
VSP13	TGME49_291180	239	14	5.5	2.55
GYF domain containing	TGME49_298610	98	12	0	NA
GRA7	TGME49_203310	1	9.5	3	3.17
Kelch repeat containing	TGME49_229000	11	9.5	2	4.75
Sec7 domain containing	TGME49_232190	139	9.5	3	3.17
GRA3	TGME49_227280	4	8.5	3	2.83
Hypothetical	TGME49_313370	74	8	2	4.00
Hypothetical	TGME49_215910	142	7.5	0	NA
Hypothetical	TGME49_288460	53	7	0	NA
Hypothetical	TGME49_287480	251	6.5	0	NA
Perforin-like protein PLP1	TGME49_204130	26	6	1	6.00
LsmAD domain containing	TGME49_231440	66	6	0	NA
Pyrroline-5-carboxylate reductase	TGME49_236070	9	6	2	3.00
Thioredoxin reductase	TGME49_309730	22	6	2	3.00
Hypothetical	TGME49_311210	71	6	0	NA
Lysine decarboxylase family	TGME49_236570	7	5.5	0	NA

a The list is sorted by the number of peptides detected in wild type (WT). Predicted *O-*GalNAc, number of glycosylation sites predicted by NetOGlyc 4.0; WT and ppGalNAc-*Δt2*, normalized peptide count detected in mass spectrometry; WT/*Δt2*, ratio of normalized peptide count between wild type and *Δt2* sample (NA represents no count detected in *Δt2* sample). The list was filtered with the semiquantitative criteria (proteins that have more than 5 peptides mapped in wild type with more than a 2.5-fold-increased detection in wild type over *Δt2* sample).

The same approach was used with the lectin VVA, and mass spectrometric analysis identified a total of 355 *T. gondii* proteins (FDR, 1.0%). Figure S4B is the Venn diagram representing the binary overlaps of VVA-isolated 355 proteins with wild-type, ppGalNAc-*Δt2*, and *Δt2*::*t2* parasites. Similarly to jacalin, VVA also had large overlap between all three genotypes. Therefore, we used the same stringent semiquantitative criteria to select T2 substrate candidates. Figure S4C shows the overlap of all proteins isolated with jacalin and VVA, indicating that a large number of proteins were common between jacalin and VVA analyses. Table 2 lists the potential ppGalNAc-T2 substrate candidate proteins identified with VVA affinity purification. Lectin overlay with VVA identified major bands at 37, 60, 70, 95, 150, and >180 kDa (Fig. 1A). From the gel corresponding to the 37-kDa microneme protein, MIC17A was identified. In the 95-kDa band, a hypothetical protein (TGME49_289540) was identified. In the 150-kDa band, inner membrane complex protein 2A (IMC2A) and a putative sortilin (TGME49_290160) were identified. From the very-high-molecular-mass gel slice (>180 kDa), VPS13-like protein, Sec7 domain-containing protein, and CST1 were detected. We did not find any potential candidates in the 60-kDa band gel. An exhaustive proteome that provides identification and characterization of all of the proteins that are responsible for the differential lectin overlay band patterns that were observed will clearly require substantial additional analysis as well as verification of the exact posttranslational modifications that are present on identified proteins.

**TABLE 2:** List of ppGalNAc-T2 substrate candidate proteins identified from the VVA affinity purification^a^

Identified protein	Accession no.	Predicted *O*-GalNAc	Peptide count		
			WT	*Δt2*	WT/Δ*t2*
IMC2A	TGME49_228170	6	44	0	NA
VSP13	TGME49_291180	239	24	0	NA
Sec7 domain containing	TGME49_232190	139	18	0	NA
Hypothetical	TGME49_212300	10	16	3	5.33
GRA7	TGME49_203310	1	15	0	NA
Hypothetical	TGME49_223070	4	9	0	NA
Cyclophilin precursor	TGME49_205700	6	8	0	NA
SRS34A	TGME49_271050	12	8	3	2.67
CST1	TGME49_264660	197	7	0	NA
Hypothetical	TGME49_289540	26	7	0	NA
Sortilin, putative	TGME49_290160	22	7	0	NA
MIC17A	TGME49_200250	7	6	0	NA
Hypothetical	TGME49_201180	3	6	0	NA

a The list is sorted by the number of peptides detected in wild type (WT). Predicted *O-*GalNAc, number of glycosylation sites predicted by NetOGlyc 4.0; WT and ppGalNAc-*Δt2*, normalized peptide count detected in mass spectrometry; WT/*Δt2*, ratio of normalized peptide count between wild type and *Δt2* sample (NA represents no count detected in *Δt2* sample). The list was filtered with the semiquantitative criteria (proteins that have more than 5 peptides mapped in wild type with more than a 2.5-fold-increased detection in wild type over *Δt2* sample).

To confirm that the identified proteins are reasonable candidates for an *O-*GalNAc posttranslational modification by ppGalNAc-T2, all identified proteins as well as the predicted *T. gondii* proteome (ME49 strain, http://toxoDB.org/toxo/) were used for an *in silico* prediction of *O-*GalNAc modifications using the support vector machine prediction algorithm NetOGlyc 4.0 (39). Figure S4D displays the kernel density plot of the number of predicted *O-*GalNAc glycosylation sites per protein in the jacalin-affinity-purified T2 substrate candidate proteins (*n* = 22), the VVA-affinity-purified T2 substrate candidate proteins (*n* = 13), or the whole *T. gondii* proteome (*n* = 8,314). A substantial fraction of T2-specific proteins have a high number (>150) of predicted glycosylation sites per protein compared with the frequency in the entire *T. gondii* proteome. The predicted average *O-*GalNAc glycosylation sites are 93.1 in the jacalin-affinity-purified ppGalNAc-T2-specific proteins and 51.7 in the VVA-affinity-purified ppGalNAc-T2-specific proteins. The number of potential *O-*GalNAc glycosylation sites is higher in both affinity-purified proteins than the calculated mean of 38.0 sites per protein for all *T. gondii* proteins. The distributions of glycosylation sites per protein (Fig. S4D) were statistically different between the whole proteome (red) and the jacalin-captured T2 candidates (green; Kolmogorov-Smirnov test, *P* < 0.05) but not between the whole proteome and the VVA-captured T2 candidates (blue; *P* > 0.05). In particular, CST1, VPS13-like, and Sec7 domain-containing proteins are present in both the jacalin and VVA eluates in a ppGalNAc-T2-dependent manner and have a large number of predicted serine or threonine potential *O-*GalNAc modification sites (197, 239, and 139 glycosylation sites, respectively). The presence of these proteins with large numbers of predicted *O-*GalNAc glycosylation sites suggests that the assay and analysis worked as expected. These lectin-based surveys in the context of ppGalNAc-Ts revealed a large variety of *T. gondii* proteins that are likely to be glycosylated by ppGalNAc-T2 and -T3 under both bradyzoite and tachyzoite conditions, suggesting that this glycosylation pathway is important in the function of many proteins in this ubiquitous eukaryotic pathogen.

### *O-*GalNAc glycosylation of CST1 mucin-like domain requires ppGalNAc-T2 and -T3.

**FIG 3:**
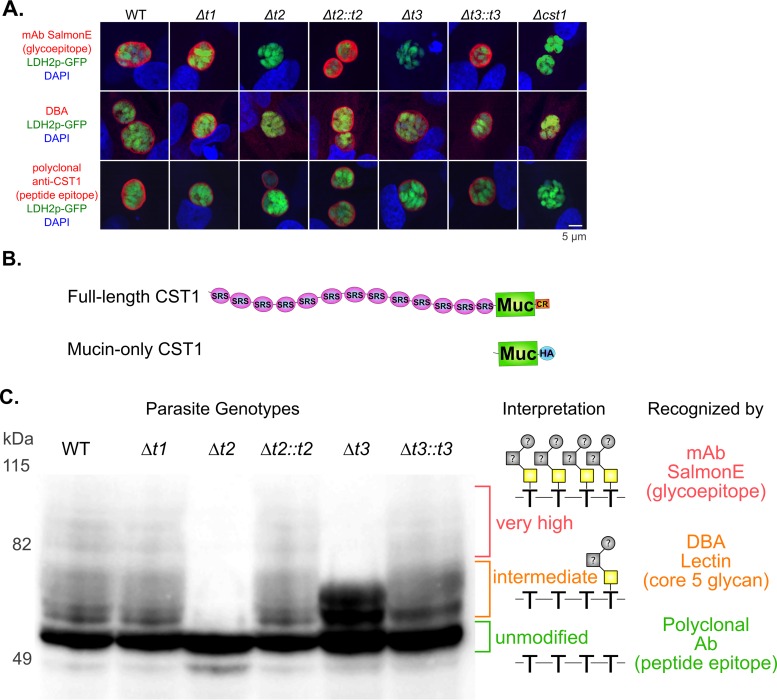
Glycosylation of CST1 mucin-like domain by ppGalNAc-T1, -T2, and -T3. (A) HFF cells were infected with *T. gondii*, cultured under bradyzoite conditions (pH 8.1) for 3 days, and then stained with either monoclonal antibody SalmonE (red), which binds to the glycoepitope of the CST1 mucin-like domain; DBA lectin (red), which also recognizes glycosylated CST1; or polyclonal anti-CST1 antibody (red), which binds to a peptide epitope of CST1. The parasites express green fluorescent protein (green) under the control of the bradyzoite marker LDH2 promoter, and cells were also stained with DAPI (blue). The cyst wall generated by the ppGalNAc-Δ*t2* and -Δ*t3* parasites completely lacks SalmonE staining. The cyst wall generated by the ppGalNAc-*Δt2* parasites also lacks DBA lectin staining; however, DBA straining is still observed in ppGalNAc-*Δt3* parasites. (B) Schematic representation of the mucin-only CST1 protein construct and the full-length CST1 construct. SRS, SAG1-like domain, 100 to 140 amino acids each; Muc, mucin-like domain, 263 amino acids; CR, cysteine-rich domain, 36 amino acids; HA, 3×HA tag, 28 amino acids. (C) *T. gondii* ppGalNAc-T strains were transiently transfected with a CST1 mucin-like domain construct containing a 3×HA tag. At 20 h after transfection, parasites were harvested from HFF cells and probed with anti-HA antibody. The ppGalNAc-Δ*t2 T. gondii* parasite demonstrates only unmodified forms of the CST1 mucin-like domain (green label), and modifications of the CST1 mucin-like domain are restored in the complemented strain (Δ*t2*::*t2*). The ppGalNAc-Δ*t3* strain lacks the very high modified forms of the CS1 mucin-like domain (red label) but has the intermediate modified forms (orange label). These very high modified forms are restored in the complemented strain (Δ*t3*::*t3*). The middle column provides a schematic representation of the *O*-GalNAc glycosylation on the mucin-like domain corresponding to the bands on the blot on the left. The right column shows the probes that recognize these suggested structures.

We previously demonstrated that cyst wall glycoprotein CST1 has a large mucin-like domain that is modified with *O-*GalNAc glycans (9). To monitor the transferase activity of ppGalNAc-Ts in their native environment, we examined the effect of each ppGalNAcT deletion on the glycosylation of the CST1 mucin-like domain. The monoclonal antibody (MAb) SalmonE binds to the glycoepitopes of the mucin-like domain of CST1 (9). The ppGalNAc-Δ*t2* and -Δ*t3* parasites lost MAb SalmonE staining of the CST1 mucin-like domain (Fig. 3A, top row), whereas MAb SalmonE staining of ppGalNAc-Δ*t1* was unchanged. Thus, the glycoepitope detected with the MAb SalmonE is due to *O-*GalNAc glycosylation of the mucin-like domain by ppGalNAc-T2 and ppGalNAc-T3. Surprisingly, only ppGalNAc-Δ*t2* parasites lost DBA lectin staining of the CST1 mucin-like domain, not ppGalNAc-*Δt3* parasites (Fig. 3A, middle row). This indicates that the glycoepitope detected with DBA lectin is modified by ppGalNAc-T2 but not by ppGalNAc-T3. These lectin-binding results are in concordance with previous data that suggested that ppGalNAc-T3 substrates require preglycosylation (25).

To verify that the mucin-like domain of CST1 is glycosylated by ppGalNAc-T2 and ppGalNAc-T3, a truncated CST1 construct that consisted of a mucin-like domain with a C-terminal 3× hemagglutinin (HA) tag driven by a strong constitutive GRA1 promoter was created (Fig. 3B). This mucin-like domain construct was transiently expressed in the various ppGalNAc-T deletion mutants. We reasoned that if the mucin-like domain is *O-*GalNAc glycosylated by ppGalNAc-T2 and ppGalNAc-T3, the molecular mass of the mucin-like domain would change in the parasites that lack a ppGalNAc-T that modifies this domain. Figure 3C demonstrates a mobility shift in the molecular mass of this mucin-only CST1 protein due to the changes in these posttranslational modifications. The unmodified mucin-only CST1 protein is predicted to have a molecular mass of 46 kDa. Consistent with this prediction, the wild-type parasites had a major SDS-PAGE band of ~50 kDa (green) with smears of high-molecular-mass proteins (yellow and red) typical for the heterogeneous nature of a multiglycosylated protein. The 4-kDa discrepancy between the major band protein mobility observed in SDS-PAGE and the predicted molecular mass of this protein could be due to an unknown posttranslational modification or to aberrant SDS-PAGE migration secondary to structural considerations related to the amino acid composition of the mucin-only CST1 protein construct. Deletion of ppGalNAc-T1 (*Δt1*) did not change the presence of these high-molecular-mass proteins. The ppGalNAc-*Δt2* parasites display the ~50-kDa major band (green), but the higher-molecular-mass glycoproteins (yellow and red) are absent. As expected, complementation of the ppGalNAc-T2 strain (*Δt2*::*t2*) restored the high-molecular-mass proteins, consistent with the high-molecular-mass proteins having multiple complex *O-*GalNAc glycan modifications that are dependent on the presence of functional ppGalNAc-T2. Interestingly, the ppGalNAc-*Δt3* parasites produced the intermediate-molecular-mass bands (60 to 80 kDa; yellow) but not the very-high-molecular-mass (80 to 100 kDa; red) bands. This lack of very-high-molecular-mass bands is rescued by the complementation of ppGalNAc-T3 (*Δt3*::*t3*). This suggests that the very-high-molecular-mass bands (80 to 100 kDa; red) are due to *O-*GalNAc modifications by ppGalNAc-T3 but that these modifications require the initial ppGalNAc-T2 activity. The ppGalNAc-*Δt3* mutant displays accumulation of the 60- to 80-kDa bands (yellow), which are likely to be products of the ppGalNAc-T2 reaction that cannot be further modified by ppGalNAc-T3. Collectively, these data strongly support the hypothesis that ppGalNAc-T2 and -T3 transfer GalNAc to their substrates in a hierarchical fashion where ppGalNAc-T2 initiates *O-*GalNAc glycosylation on a threonine residue of an unglycosylated mucin-like domain. Subsequently, T3 recognizes this initial *O*-GalNAc and glycosylates the adjacent acceptor threonine sites, filling in the rest of the glycosylation sites on the threonine-rich tandem repeat (Fig. 3C).

### Lack of *O-*GalNAc glycosylation by ppGalNAc-T2 and -T3 on CST1 mucin-like domain results in the formation of fragile brain cysts.

**FIG 4:**
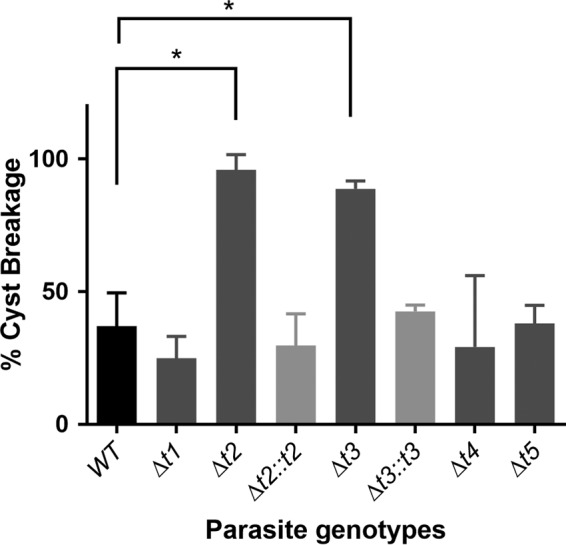
Brain cyst rigidity is dependent on the presence of ppGalNAc-T2 and -T3. BALB/c^dm2−/−^ mice were infected with *T. gondii* strains as indicated in Materials and Methods. Three weeks postinfection, the brains of infected mice were harvested and homogenized with or without paraformaldehyde fixation to determine the fragility of brain cysts that developed *in vivo*. The difference between wild-type (WT) and ppGalNAc-Δ*t2 T. gondii* as well as that between WT and ppGalNAc-Δ*t3 T. gondii* was statistically significant (asterisks; one-way ANOVA and Dunnett *post hoc* test; *P* < 0.05), demonstrating that glycosylation is necessary for cyst stability. Wild-type *T. gondii*, black bars; ppGalNAc deletion *T. gondii* mutants, dark gray bars; ppGalNAc-complemented *T. gondii* strains, light gray bars.

Our previous study demonstrated that lack of the mucin-like domain of CST1 resulted in the formation of fragile brain cysts in a chronic *T. gondii* infection mouse model (9). To investigate whether glycosylation on the mucin-like domain of CST1 by ppGalNAc-T2 and ppGalNAc-T3 is required for brain cyst rigidity, we quantified the mouse brain cyst breakage rate in the ppGalNAc-T deletion mutants. Briefly, brain cysts were harvested at 3 weeks postinfection. One half of each brain was fixed overnight with 4% paraformaldehyde to keep the brain cysts intact and then homogenized to determine total number of brain cysts. The other half of each brain was homogenized without fixation to determine how many cysts could withstand the mechanical stress. The breakage rates of ppGalNAc-Δ*t2* and -Δ*t3* brain cysts were significantly higher than that of the wild type (Fig. 4) (*P* < 0.05, one-way analysis of variance [ANOVA] and Dunnett *post hoc* test). Cysts from complemented strains that had a cDNA copy of ppGalNAc-Ts (Δ*t2*::*t2* and Δ*t3*::*t3*) resulted in brain cysts with rigidity similar to that of the wild type. This suggests that the rigidity of the brain cyst is dependent on the presence of both ppGalNAc-T2 and -T3. Although both ppGalNAc-T2 and -T3 glycosylate the mucin-like domain of CST1 and other parasite proteins, the cyst wall rigidity phenotype is most likely due to the reduced glycosylation of the CST1 mucin-like domain, as our previous data revealed that deletion of the mucin-like domain from CST1 was sufficient in itself to cause the fragile cyst phenotype (9).

## DISCUSSION

This paper demonstrates that *T. gondii* ppGalNAc-T2 transfers GalNAc to proteins that subsequently become *O-*GalNAc glycosylated by T3 in a hierarchical manner (summarized in Fig. S5 in the supplemental material). Previous cell-free catalytic studies have documented that there is a group of ppGalNAc-Ts that require *O-*GalNAc preglycosylation on the acceptor peptide (e.g., *T. gondii* T1 and T3 [18, 25, 26] and human T7 and T10 [29, 40]). It has been hypothesized that there are initiating ppGalNAc-Ts that transfer GalNAc to the unmodified peptide and filling-in ppGalNAc-Ts that subsequently transfer GalNAc to neighboring acceptor sites sequentially (41). Mass spectrometric analysis of heterogeneously glycosylated proteins from organisms having a hierarchical pattern of *O-*GalNAc occupancy supports this model (42, 43).

10.1128/mBio.02048-16.5 FIG S5  A model of hierarchical *O-*GalNAc glycosylation of the CST1 mucin-like domain. Represented is a single unit of the threonine-tandem repeat of CST1 mucin-like domain. One unit has 5 to 11 threonine residues, and the mucin domain has 20 repeats of the unit. The process proceeds in four stages. (1) Initiation: the furthest C-terminal end threonine gets *O-*GalNAc glycosylated by *T. gondii* ppGalNAc-T2. (2) Filling-in: following initiation, ppGalNAc-T3 binds to the *O-*GalNAc on the peptide and transfers another *O-*GalNAc to the threonine at the −1 position. (3) Sequential glycosylation: ppGalNAc-T3 then binds to the newly added *O-*GalNAc and sequentially glycosylates adjacent threonine residues in the N-terminal direction of the peptide until the end of the repeat is reached. (4) Extension: once the *O-*GalNAc is in position, other glycosyltransferases add sugars to form core 5 structure (yellow) and fully extended glycan modifications. Download FIG S5, TIF file, 1.1 MB. Copyright © 2017 Tomita et al. 2017 Tomita et al. This content is distributed under the terms of the Creative Commons Attribution 4.0 International license.

Our study demonstrates that hierarchical *O-*GalNAc glycosylation of the CST1 mucin-like domain by sequential ppGalNAc-T glycosylation occurs in an intact organism and that this modification is biologically significant. The highly redundant nature of ppGalNAc-Ts (e.g., in humans there are 20 homologous genes [reviewed in reference 34]) renders the mammalian system difficult for investigation of the function of ppGalNAc-Ts in their native environment within cells. In contrast, *T. gondii* has only three isoforms constitutively expressed under our *in vitro* culture conditions, which enabled us to evaluate individual ppGalNAc-T functions. To this end, *T. gondii* is an ideal model organism to specifically distinguish otherwise overlapping ppGalNAc-T activities and study in depth the functions of various ppGalNAc-T isoforms.

A critical question that remains to be answered is the exact structure of the glycans that are present on the mucin-like domain of CST1. We have demonstrated that DBA lectin binds to the CST1 mucin-like domain even in the absence of T3 activity. This suggests that after T2 transfers GalNAc to the threonine of the mucin-like domain, forming Tn antigen (GalNAcα1-Thr), an as-yet-unknown glycosyltransferase adds additional GalNAc to the Tn antigen to form the core 5 structure (GalNAcα1,3GalNAcα1-Thr) that is detected by the DBA lectin in *T. gondii* (Consortium of Functional Glycomics Glycan Array Database [44]). Very little is known about the biological importance of this core 5 structure. Core 5 is rare in the mammalian systems (45), being detected only in the human meconium (46), and its sialylated forms are found in human adenocarcinoma (47) and bovine submaxillary mucin (48). However, in other eukaryotes, core 5 and its extended glycans (with GalNAc, Al, Fuc, and Neu5Ac) are the dominant glycans found; for example, core 5 is abundant in the skin and gastrointestinal tract of both Atlantic salmon (49) and rainbow trout (50). The biological significance of the core 5 structure that is present on the mucin-like domain of CST1 (which is similar to what is described in fish mucus) merits further investigation.

A previous enzymatic study in a cell-free system revealed that recombinant T3 requires pre-*O-*GalNAc glycosylation on an adjacent threonine at the +1 position relative to the acceptor threonine residue of peptide substrates (25). CST1 has 20 threonine-rich tandem repeats of T_5–11_[R/I]K_2_P. Based on our data, it is probable that the furthest C-terminal threonine gets glycosylated by T2 (Fig. 3C and S5), resulting in the observed increase of about 8 kDa (0.2 kDa [GalNAc] × 2 [for core 5] × 20 repeats = 8 kDa) on SDS-PAGE (Fig. 3C, ppGalNAc-*Δt2* and -Δ*t3* parasites). Based on the size of the bands observed in our experiments, there are likely additional extensions of that glycan by two to seven more sugars to form the higher-molecular-mass bands that are 16 to 28 kDa larger than the unglycosylated CST1 mucin-like domain. It is probable that ppGalNAc-T3 transfers GalNAc to threonine on the −1 position relative to the initial *O-*linked GalNAc modified by ppGalNAc-T2 (Fig. S5) and then sequentially ppGalNAc-T3 fills in the rest of the threonine residues in each repeat from the −1 position toward the N terminus until the end of a particular repeat is reached. Our current working model of the hierarchical *O-*GalNAc glycosylation of CST1 mucin-like domain by ppGalNAc-T2 and -T3 is summarized in Fig. S5.

Our initial observation of the CST1 role in cyst wall structural rigidity demonstrated using deletion mutants of CST1 domains that the mucin-like domain of CST1 is necessary for the *in vivo* phenotype (9). The work presented in this paper demonstrates that the presence of the mucin-like domain and the proper glycosylation of that mucin-like domain by both ppGalNAc-T2 and -T3 are necessary for the function of this critically important cyst wall structural protein. The exact molecular mechanism(s) of how CST1 and its posttranslational glycosylation result in structural integrity of the cyst wall and the assembly of this structure remain to be elucidated.

## MATERIALS AND METHODS

### Parasite strains and generation of ppGalNAc-T mutants.

Parasites were maintained in human foreskin fibroblasts (HFF; ATCC-CRL-2522) in Dulbecco modified Eagle medium (DMEM) (Gibco Life Technologies) with 10% fetal bovine serum (FBS: HyClone/GE Healthcare Life Science) and penicillin-streptomycin (Gibco Life Technologies) at 5% CO_2_. For the generation of ppGalNAc-T mutants, we used the type II Prugniaud (Pru) *Δku80 Δhxgprt T. gondii* strain as the parental strain (32) (kindly provided by David Bzik, Dartmouth College). This strain is capable of differentiating *in vitro* and amenable to the targeted deletion due to the lack of nonhomologous recombination. All of the deletion plasmids (ppGalNAc-pΔt1 to -pΔt5) were developed using the pUC19 vector and In-Fusion system (TaKaRa Clontech) in which the following vector fragments were assembled into the plasmid: a 1-kb 5′ upstream homologous arm region fragment, a 2-kb hypoxanthine-xanthine-guanine phosphoribosyltransferase (*HXGPRT*) cassette fragment, and a 1-kb 3′ downstream homologous arm region fragment with a 15-nucleotide overlap. The fragments were amplified using Phusion DNA polymerase (Thermo Fisher) according to the manufacturer’s protocol. The homologous arms were designed to completely remove each targeted ppGalNAc-T gene from its respective start codon to its stop codon. Transfections were performed in Cytomix, and parasites were selected using 25 µg/ml mycophenolic acid and 50 µg/ml xanthine as previously described (9). Complementation of the gene knockouts was performed for the Δ*t2* and Δ*t3* parasite strains. A cDNA copy of ppGalNAc-T2 was inserted into the T2 endogenous locus using a DHFR selectable marker. A cDNA copy of ppGalNAc-T3 was inserted into the UPRT locus along with its 1.5-kb endogenous promoter. Verification of deletion and complementation was performed with diagnostic PCR (Fig. S2) and sequencing of all amplicons used for vector constructions (data not shown). All the plasmids used in the deletion and complementation procedure are provided in Text S1 as GenBank format sequence files.

10.1128/mBio.02048-16.7 TEXT S1  Annotated sequences of the plasmids used in this study. The zip file contains annotated sequences of plasmids in GenBank format. Primers used for the cloning are annotated in these files. The plasmids used for ppGalNAc-T1, -T2, -T3, -T4, and -T5 deletion mutant establishment are pUC19-T1KO, -T2KO, -T3KO, -T4KO, and -T5KO vectors, respectively. The plasmids used for the complementation of ppGalNAc-T2 and -T3 are pT2CompDHFRv2_correctP5RT70 and pT3Comp, respectively. For the CST1 mucin-like domain expression, the pSMARTBAC2-UPRT-DHFR-GRA1p-TwStrp-MucOnly-3HA plasmid was used. Download TEXT S1, PDF file, 0.2 MB. Copyright © 2017 Tomita et al. 2017 Tomita et al. This content is distributed under the terms of the Creative Commons Attribution 4.0 International license.

### Lectin overlay.

The parasites were cultured in DMEM with 10% bovine growth serum (BGS; HyClone) for 3 days in 5% CO_2_. Harvested parasites were passed through a 27-gauge needle three times and then filtered through a 5-µm polycarbonate membrane to remove host cells. Parasites were lysed in 2% NP-40 in phosphate-buffered saline (PBS) containing cOmplete EDTA-free protease inhibitor (Thermo Fisher), and the lysates were cleared by centrifugation. The supernatants were boiled with sample buffer and separated by 4 to 15% SDS-PAGE (15 µg/lane), and the separated proteins were transferred to either a nitrocellulose or a PVDF membrane. The membrane was incubated with each lectin (at 10 µg/ml) for 1 h at room temperature. After washing three times with PBS, the membranes were incubated with streptavidin-horseradish peroxidase (HRP) at 1:20,000, and subsequently, the signal was detected using SuperSignal West Femto maximum-sensitivity substrate (Thermo Fisher). Biotin-conjugated jacalin, ConA, DBA, s-WGA, SNA-I, GS-I, VVA, and HPA (EY Laboratories) were evaluated for their ability to bind to the SDS-PAGE-resolved protein bands. β-elimination of duplicate membranes was performed, to confirm *O*-glycosylation, using the previously described methods (51): e.g., the membrane was incubated with 0.1 N NaOH for 16 h at 40°C in a rotating hybridizer chamber, rinsed with PBS three times, blocked with bovine serum albumin (BSA), and then incubated with the lectins as described above.

### Lectin-binding cytochemistry.

HFF cells cultured on cover glasses were infected with *T. gondii* in pH 8.1 DMEM containing 1% FBS and 50 mM HEPES at atmospheric CO_2_ (0.1%) for 3 days. The cells were fixed with 4% paraformaldehyde in PBS for 30 min and then permeabilized with 0.2% Triton X-100 in 3% BSA in PBS for 20 min. The cells were washed with PBS three times and blocked with 3% BSA in PBS at 4°C overnight. Cover glasses with cultured, fixed, permeabilized cells were then incubated with different biotin-conjugated lectins (DBA, SNA-I, ConA, jacalin, GST, WGA, HPA, and VVA) at 10 µg/ml in PBS for 90 min at 37°C and washed with PBS three times. Afterward, a rabbit antibiotin antibody at a 1:500 dilution was incubated with the cover glasses for 90 min at 37°C and they were washed with PBS three times. Anti-rabbit Alexa Fluor 488 at a 1:1,000 dilution was incubated with cover glasses for 90 min at 37°C, and they were washed with PBS three times. Following this washing procedure, each cover glass was mounted on a slide using ProLong Gold antifade reagent containing 4′,6-diamidino-2-phenylindole (DAPI) (Thermo Fisher). Each cover glass was examined using a confocal fluorescence microscope (SP5; Leica).

### Jacalin and VVA affinity chromatography.

Parasites were cultured under tachyzoite conditions, harvested, and filter purified as described above. Purified *T. gondii* (2 × 10^10^ parasites) was lysed in 1 ml PBS (containing 0.5% NP-40, 500 mM NaCl, and cOmplete protease inhibitor cocktail) and then incubated for 2 h on ice. The samples were centrifuged at 13,600 × *g* at 4°C for 16 min, and the collected supernatants were applied to either a jacalin- or a VVA-agarose bead affinity column (EY Laboratories). The columns were washed with 300 μl of phosphate buffer three times and incubated for 15 min with 200 μl of elution buffer, and the eluate was collected. The eluates were separated by SDS-PAGE on a 4 to 20% gradient gel and visualized with Coomassie blue staining. Each lane was cut into 24 bands, and the bands were processed for proteomic analysis as described previously (52). Briefly, the gel bands underwent trypsin digestion followed by liquid chromatography-tandem mass spectrometry (LC-MS/MS) on a Rapid Separation LC3000 system (Dionex Corporation, Sunnyvale, CA) and a linear trap quadrupole (LTQ) linear ion trap mass spectrometer (Thermo, San Jose, CA). The LTQ was operated in the data-dependent ion tree depth mode with a maximum breadth and depth of 2 and 3, respectively. With these settings, the top two abundant ions (with charge states of 2 and above) determined from a 300.0 to 1,800.0 *m/z* survey scan will undergo MS/MS (or MS2) and the top two product ions from the MS2 scan will undergo MS3 fragmentation. MS*n* was performed using an isolation width of 2 *m/z*, normalized collision energy of 35%, and activation time of 30 ms. Protein identification was performed with the Mascot protein search engine (Matrix Science) using these parameters: fragment ion mass tolerance of 0.80 Da and parent ion tolerance of 2.0 Da. Carbamidomethyl of cysteine was specified in Mascot as a fixed modification. Glu→pyro-Glu of the N terminus, Gln→pyro-Glu of the N terminus, deamidated of asparagine and glutamine, and oxidation of methionine were specified in Mascot as variable modifications. Scaffold (Proteome Software) was used for protein peptide validation at 99% and 95% minimum protein and peptide, respectively, with a minimum number of 2 peptides. A decoy database was employed to obtain false discovery rates which were 1% and 0.06% at the protein and peptide levels, respectively. Kernel density plot Venn diagrams were generated using ggplot2 and venneuler packages, respectively. Kolmogorov-Smirnov statistical analysis was performed using R with default parameters.

### Mucin-like domain CST1 expression.

A mucin-like domain-containing construct in a pSMART-BAC2 bacmid (Lucigen) was generated using the HiFi DNA assembly kit (New England BioLabs [NEB]). From the 5′ direction, this vector contains a GRA1 promoter, CST1 signal peptide sequence, tandem strep-II tag, CST1 mucin-like domain, and then a 3×HA tag sequence (annotated sequence is presented in Text S1). The mucin-like domain was amplified from a CST1 cDNA-containing bacmid (9) derived from the type II *T. gondii* ME49 strain. After the sequence was verified, PCR fragments spanning from the GRA1 promoter to the 3′ untranslated region (UTR) were generated using Q5 DNA polymerase (NEB). Five micrograms of the PCR fragments was transfected to the ppGalNAc-T deletion mutants using the Amaxa Nucleofector system with basic parasite kit 1 (Lonza) and the U-33 program. Parasites, cultured under the tachyzoite condition, were harvested 20 h after transfection by scraping, including host HFF cells. Cells were pelleted, lysed in 100 µl of SDS sample buffer, and boiled for 10 min. For each lane, 40 µl of sample was run on an SDS-PAGE gel. After transfer to the PVDF membrane, the membrane was blocked with 5% milk in PBS and incubated with HRP-conjugated rat anti-HA antibody at 1:1,000 overnight. Signal was detected using the Odyssey Fc system (Li-Cor).

### Brain cyst fragility assay.

The brain cyst fragility assay was done as described previously (9). Briefly, BALB/c^dm2^ mice having a deletion in the *L*^*d*^ gene at the *H-2L* locus, which produce more brain cysts than the wild type (53), were infected with 1,000 parasites of each strain intraperitoneally. Three weeks after infection, brains were harvested. One half of each brain was fixed in 4% paraformaldehyde in PBS for 1 day at 4°C. The raw and fixed halves were then ground with a glass tissue homogenizer (size A, 0.1- to 0.15-mm clearance; Thomas Scientific) to assess the fragility of the brain cysts. Brain cyst fragility rates were calculated as (number of cysts observed in raw brain halves/number of cysts observed in fixed brain halves) × 100. To test statistical significance, one-way ANOVA and Dunnett *post hoc* multiple comparison tests were performed using GraphPad Prism 6 software (GraphPad Software).

### Ethics statement.

All animal experiments were conducted in accordance with the Public Health Service Policy on Humane Care and Use of Laboratory Animals (Office of Laboratory Animal Welfare, National Institutes of Health) and the National Research Council *Guide for the Care and Use of Laboratory Animals* (54). Animals were maintained in an AAALAC-approved facility, and all protocols were approved by the Institutional Care Committee of the Albert Einstein College of Medicine, Bronx, NY (Animal Protocol 20150908; Animal Welfare Assurance number A3312-01). No human samples were used in these experiments. Human foreskin fibroblasts for tissue culture were obtained from ATCC (ATCC-CRL-2522).
