# Supplementary figures and images for: Making Home Sweet and Sturdy: Toxoplasma gondii ppGalNAc-Ts Glycosylate in Hierarchical Order and Confer Cyst Wall Rigidity
Source: mBio. 2017 Jan 10;8(1):e02048-16. doi: 10.1128/mBio.02048-16 (PMC5225312; doi:10.1128/mBio.02048-16)

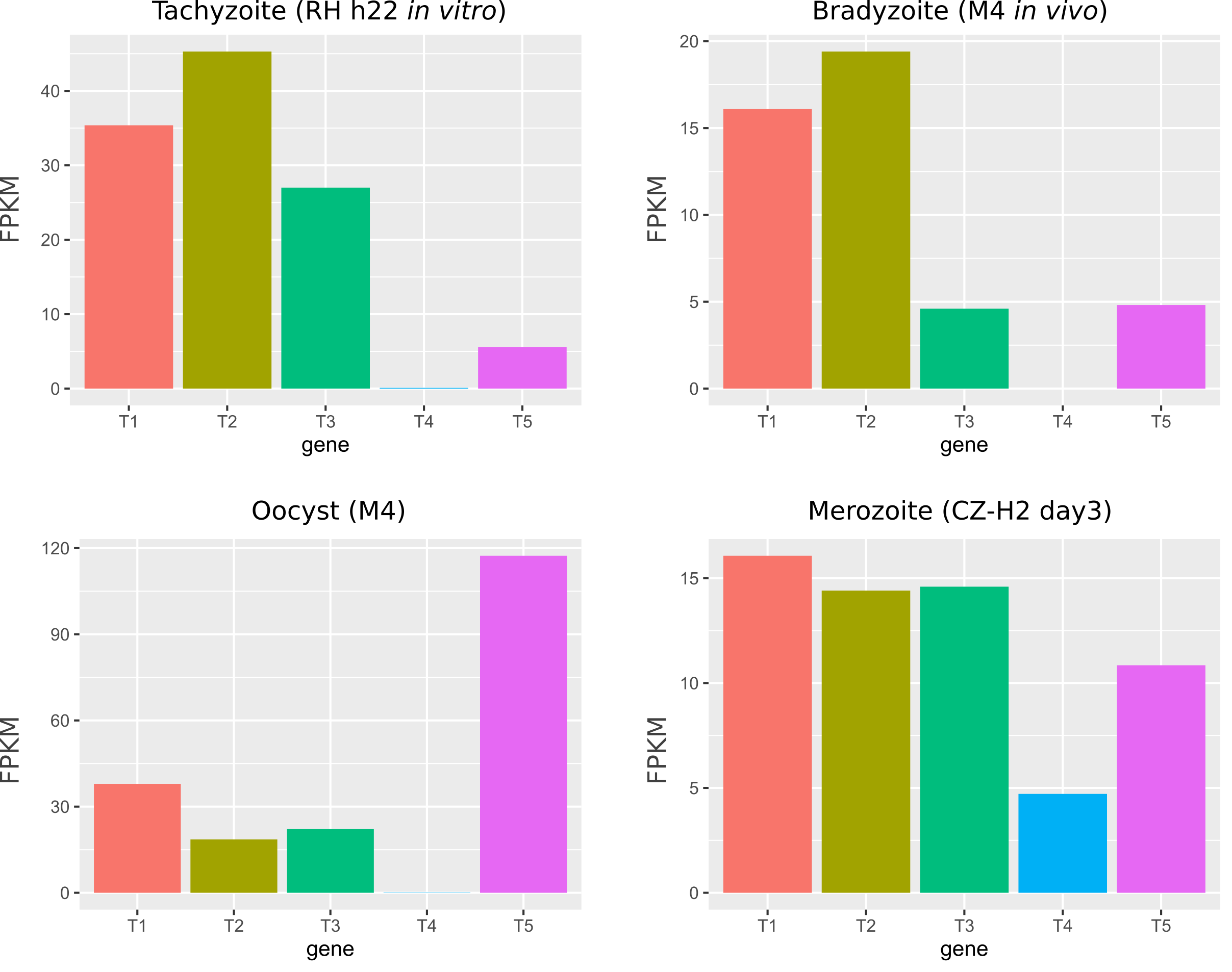

Supplement: FIG S1 [file mbo001163142sf1.tif]

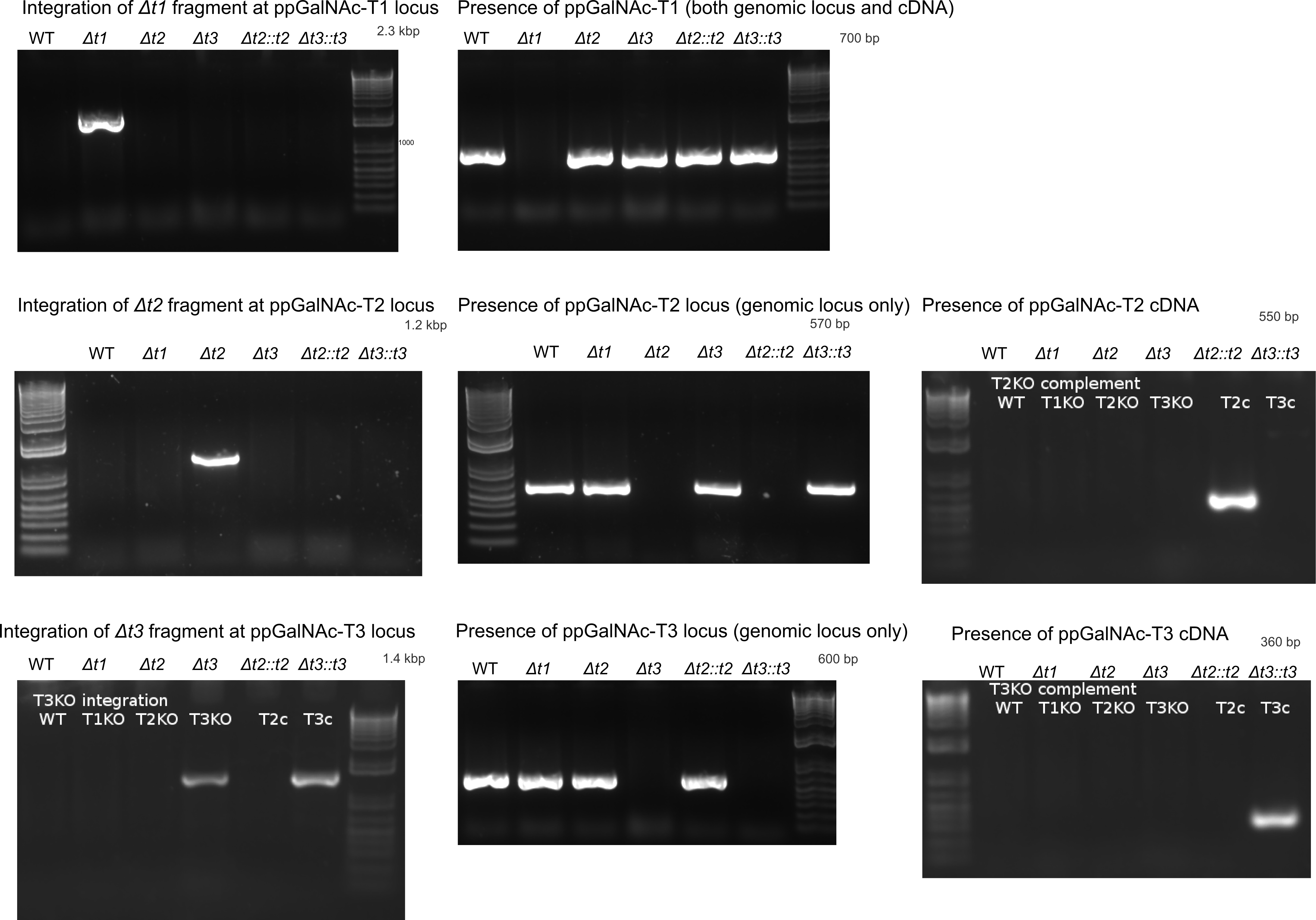

Supplement: FIG S2 [file mbo001163142sf2.tif]

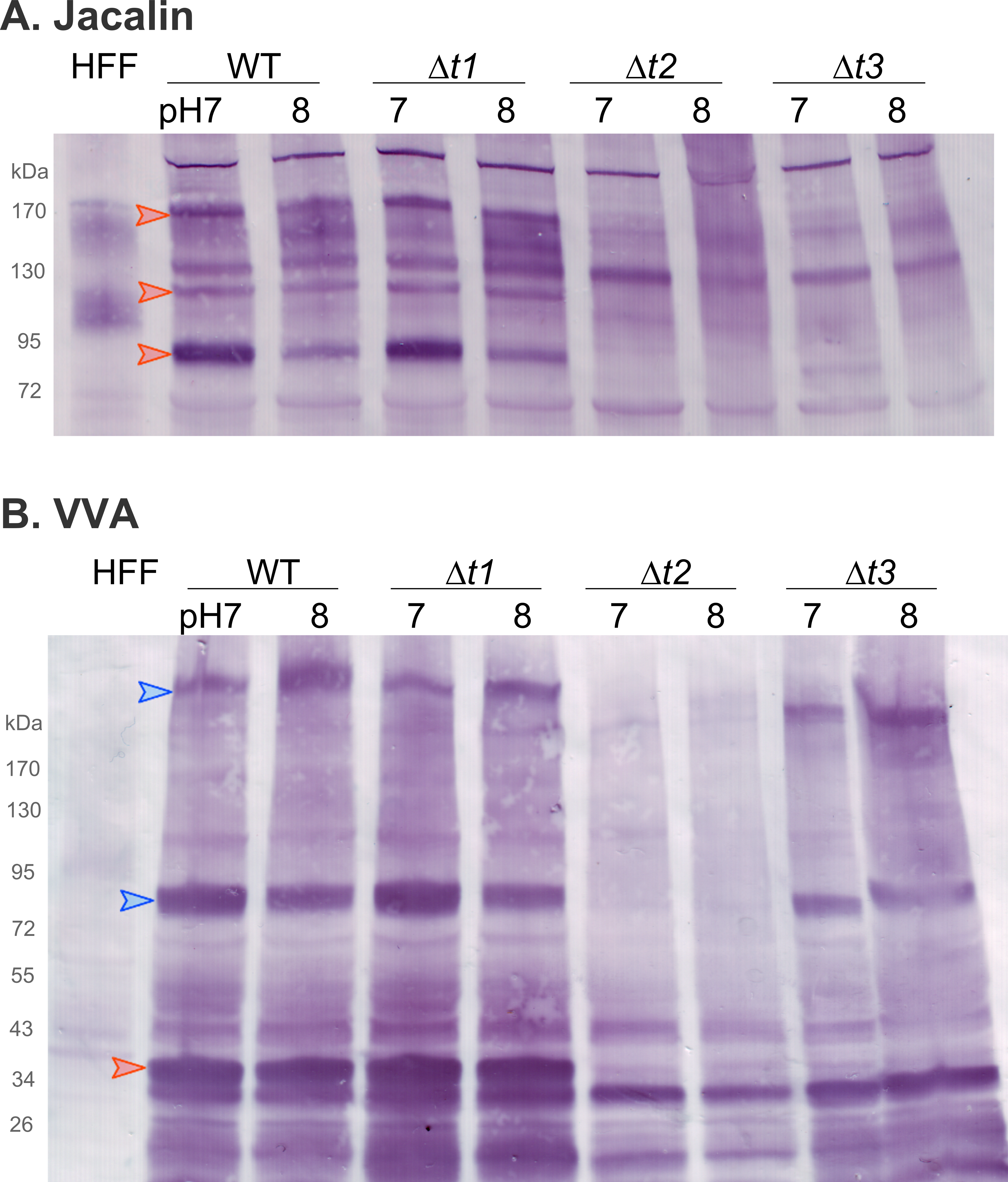

Supplement: FIG S3 [file mbo001163142sf3.tif]

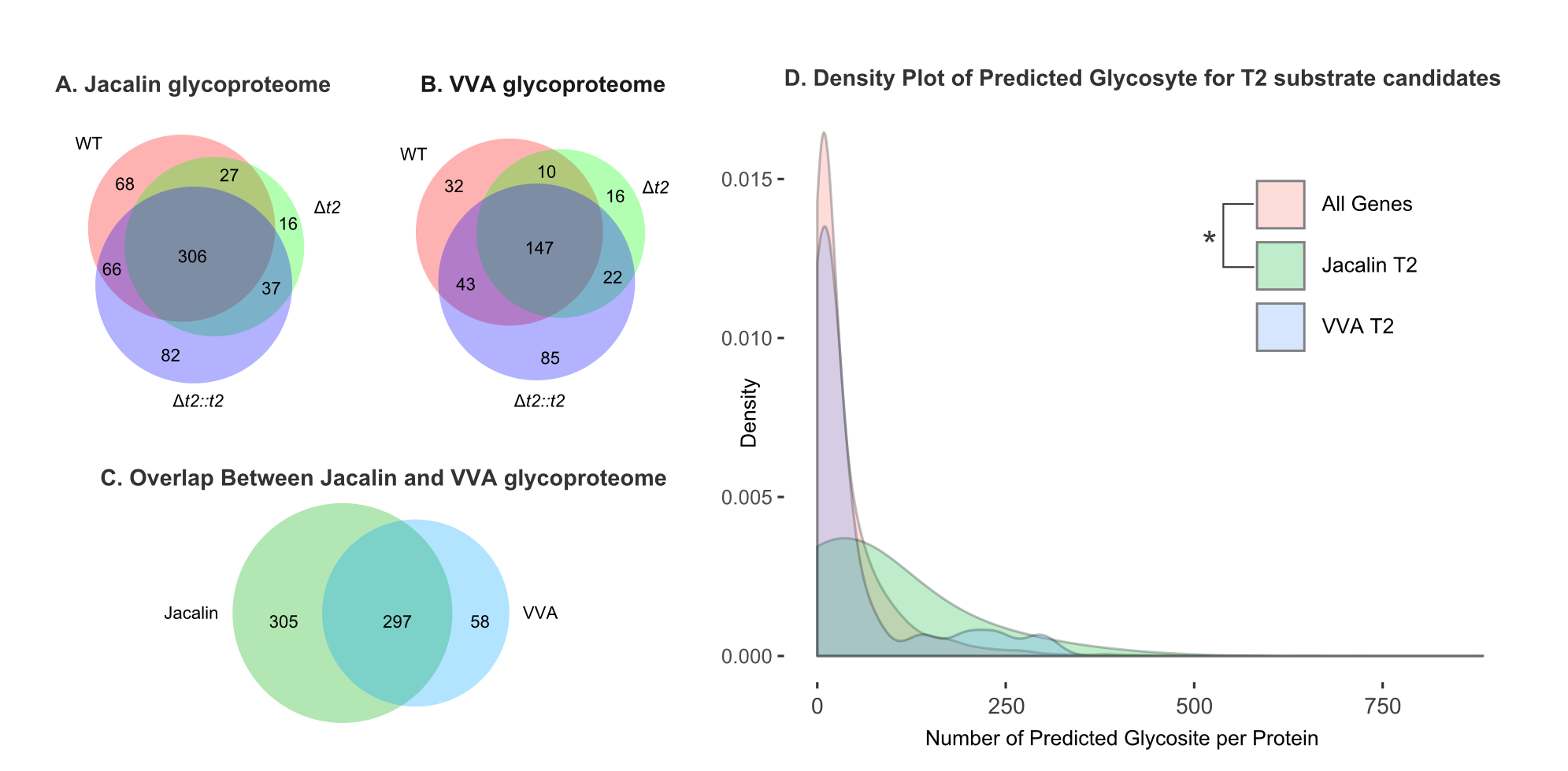

Supplement: FIG S4 [file mbo001163142sf4.tif]

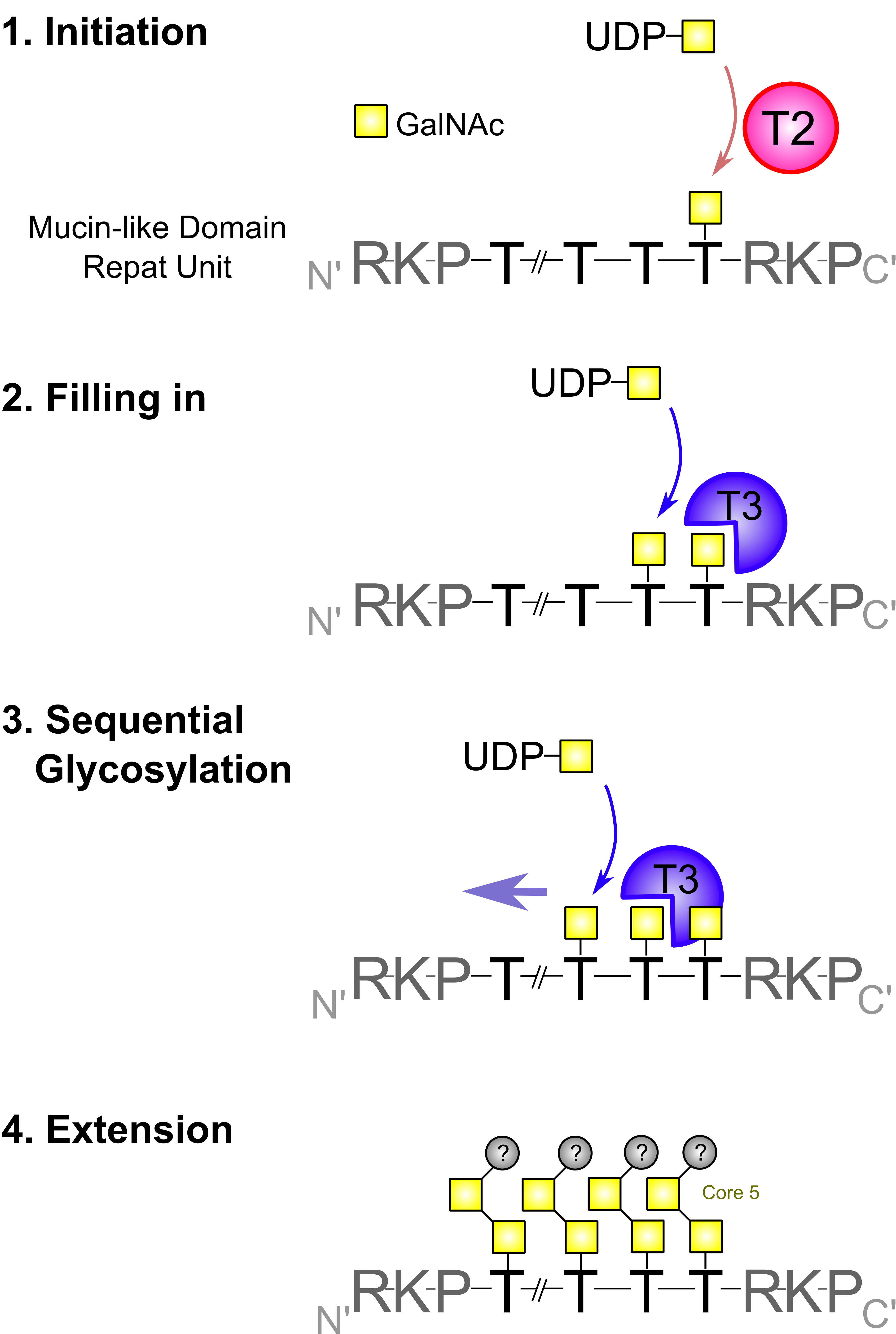

Supplement: FIG S5 [file mbo001163142sf5.tif]
