# Supplementary material for: Making Home Sweet and Sturdy: Toxoplasma gondii ppGalNAc-Ts Glycosylate in Hierarchical Order and Confer Cyst Wall Rigidity
Source: mBio. 2017 Jan 10;8(1):e02048-16. doi: 10.1128/mBio.02048-16 (PMC5225312; doi:10.1128/mBio.02048-16)
Supplement: TABLE S1 [file mbo001163142st1.docx]

**Table S1. List of lectins used for lectin survey**

| Name | Specificity |
| --- | --- |
| Jacalin | GalNAcα1-R with free C6-OH (Core1 / Core3) |
| VVA | GalNAcα1-R preferably Thr, or terminal GalNAc |
| HPA | GalNAcα1-R preferably Ser, or terminal GalNAc |
| DBA | GalNAcα1-3GalNAc-R or terminal |
| Con A | Terminal Manα1-6(Manα1-3)Man- (N-lined glycans) |
| s-WGA | Terminal GlucNAc |
| SNA-1 | NeuAcα2-6Gal-R or terminal |
| GSL-1 | Gal1α-3Gal-R or terminal |
