# Supplementary material for: Making Home Sweet and Sturdy: Toxoplasma gondii ppGalNAc-Ts Glycosylate in Hierarchical Order and Confer Cyst Wall Rigidity
Source: mBio. 2017 Jan 10;8(1):e02048-16. doi: 10.1128/mBio.02048-16 (PMC5225312; doi:10.1128/mBio.02048-16)
Supplement: TEXT S1 [file mbo001163142s1.pdf]

```
LOCUS       pUC19_T1KO_vecto        7001 bp    T1KO    DNA
    vecto 27-APR-2014
DEFINITION  Concatenation of 7 sequences.
ACCESSION   urn.local...1317843251376.24
VERSION     urn.local...1317843251376.24
KEYWORDS    .
SOURCE
  ORGANISM
            .
UNIMARK     pUC19 T1KO vecto features
            pUC19 T1KO vecto



ORIGIN
Features
     source          1..415
     /organism="Cloning vector pUC19"
     /mol_type="other DNA"
     /label="source Cloning vector pUC19"
```

```
     misc_feature    1..415
     /label="pUC19 5' (concatenated sequence 1)"
     /note="Geneious type: Concatenated sequence"
```

```
     misc_feature    1..7001
     /5'term=""
     /3'term=""
     /5'overhang=""
     /3'overhang=""
     /5'strand="direct"
     /3'strand="direct"
     /5'type="blunt"
     /3'type="blunt"
     /fragment_source="pUC19 T1KO vecto"
     /ugene_name="Fragment (1-7001)"
     /ugene_group="fragments"
```

```
     CDS             complement(146..415)
     /gene="lacZalpha"
     /codon_start=1
     /product="beta-galactosidase alpha fragment"
     /translation="MTMITPSLHACRSTLEDPRVPSSNSLAVVLQRRDWENPGVTQLNRLAAHPPFASWRNSEEARTDRPSQQLRSLNGEWRLMRYFLLTHLCGISHR[illegible]
     STICSDAA"
     /label="lacZalpha CDS"
```

```
     gene            complement(146..415)
     /gene="lacZalpha"
     /label="lacZalpha gene"
```

```
     misc_feature    183..188
     /Recognition_pattern="CA^TATG"
     /annotation_group="CATATG"
     /label="NdeI"
     /note="Geneious type: restriction site"
```

```
     misc_feature    245..255
     /Recognition_pattern="GCCNNNN^NGGC"
```

```
     /label="lacZalpha gene"
misc_feature        183..188
     /Recognition_pattern="CA^TATG"
     /annotation_group="CATATG"
     /label="NdeI"
     /note="Geneious type: restriction site"
misc_feature        245..255
     /Recognition_pattern="GCCNNNN^NGGC"
     /annotation_group="GCCNNNNNGGC"
     /label="BglI"
     /note="Geneious type: restriction site"
primer_bind         353..374
     /created_by="User"
     /modified_by="User"
     /label="M13F"
misc_feature        complement(391..415)
     /created_by="User"
     /label="pUC19productionR-Smi"
     /note="Geneious type: primer_bind_reverse"
misc_feature        396..415
     /gene="lacZalpha"
     /note="multiple cloning site (EcoRI-HindIII)"
     /label="lacZalpha misc"
misc_feature        396..401
     /Recognition_pattern="G^AATTC"
     /annotation_group="GAATTC"
     /label="EcoRI"
     /note="Geneious type: restriction site"
primer_bind         401..439
     /created_by="User"
     /label="T1ko-5'F /adp"
misc_feature        402..407
     /Recognition_pattern="GAGCT^C"
     /annotation_group="GAGCTC"
     /label="SacI"
     /note="Geneious type: restriction site"
misc_feature        412..415
     /created_by="User"
     /modified_by="User"
     /label="SmiI5"
     /note="Geneious type: restriction_site"
source              416..419
```

```
/organism="Cloning vector pUC19"
/mol_type="other DNA"
/label="source Cloning vector pUC19"
misc_feature        416..419
/created_by="User"
/modified_by="User"
/label="SmiI3"
/note="Geneious type: restriction_site"
misc_feature        416..419
/label="SmiI3 add first (concatenated sequence 2)"
/note="Geneious type: Concatenated sequence"
misc_recomb         420..1502
/created_by="User"
/label="5'Recomb"
misc_feature        420..1502
/label="T1ko - 5'Recomb (concatenated sequence 3)"
/note="Geneious type: Concatenated sequence"
primer_bind         420..439
/created_by="User"
/modified_by="User"
/label="T1ko-5'F"
CDS                 609..1061
/Source="annotation"
/isDeprecated=0
/label="CDS"
gene                609..1061
/Source="annotation"
/Note="hypothetical protein, conserved"
/soTerm="protein_coding"
/product="hypothetical protein, conserved"
/taxon="Toxoplasma gondii ME49"
/isDeprecated=0
/isPseudo=0
/label="TGME49_059540"
misc_feature        complement(1483..1517)
/created_by="User"
/label="T1ko-5'R w/adp"
/note="Geneious type: primer_bind_reverse"
primer_bind         complement(1483..1502)
/created_by="User"
/modified_by="User"
/label="T1ko-5'R"
```

```
misc_feature        1503..1508
/created_by="User"
/label="KpnI"
/note="Geneious type: restriction_site"
```

```
misc_feature        1503..3442
/label="HXGPRT w/ KpnI (concatenated sequence 4)"
/note="Geneious type: Concatenated sequence"
```

```
primer_bind         1503..1529
/created_by="User"
/label="HXGPRT-KpnIF"
```

```
gene                1509..3438
/created_by="User"
/label="DHFR-HXGPRT"
```

```
misc_feature        complement(1722..1744)
/created_by="User"
/label="5ex2R"
/note="Geneious type: primer_bind_reverse"
```

```
misc_feature        1971..1973
/Original_Base=""
/labe=""
/note="Geneious type: Editing History Insertion"
```

```
misc_feature        1977..2669
/label="HXGPRT"
/ugene_name="Selectable_Mark"
```

```
primer              1984..2003
/note="Oligo predicted by CLC primer designer"
/note="Oligo type: primer"
/note="Melting temp.: 56.07"
/label="HXGPRT detection"
```

```
primer              complement(2699..2720)
/note="Oligo predicted by CLC primer designer"
/note="Oligo type: primer"
/note="Melting temp.: 54.02"
/label="HXGPRT detection"
```

```
misc_feature        complement(3422..3442)
/created_by="User"
/label="HXGPRT-KpnIR"
/note="Geneious type: primer_bind_reverse"
```

```
primer_bind         3428..3463
/created_by="User"
/label="T1ko-3'F w/adp"
```

```
misc_feature        3437..3442
```

```
     /created_by="User"
     /modified_by="User"
     /label="KpnI"
     /note="Geneious type: restriction_site"
---
     misc_recomb         3443..4723
     /created_by="User"
     /label="3'Recomb"
---
     misc_feature        3443..4723
     /label="T1ko - 3'Recomb (concatenated sequence 5)"
     /note="Geneious type: Concatenated sequence"
---
     primer_bind         3443..3463
     /created_by="User"
     /label="T1ko-3'F"
---
     misc_feature        3443..4284
     /Source="annotation"
     /isDeprecated=0
     /label="UTR"
     /ugene_name="UTR"
---
     gene                3443..4284
     /Source="annotation"
     /Note="UDP-N-acetyl-D-galactosamine:polypeptide
     N-acetylgalactosaminyltransferase T1, putative"
     /soTerm="protein_coding"
     /product="UDP-N-acetyl-D-galactosamine:polypeptide
     N-acetylgalactosaminyltransferase T1, putative"
     /taxon="Toxoplasma gondii ME49"
     /isDeprecated=0
     /isPseudo=0
     /label="TGME49_059530"
---
     misc_feature        complement(4703..4742)
     /created_by="User"
     /label="T1ko-3'R w/adp"
     /note="Geneious type: primer_bind_reverse"
---
     primer_bind         complement(4703..4723)
     /created_by="User"
     /label="T1ko-3'R"
---
     source              4724..4727
     /organism="Cloning vector pUC19"
     /mol_type="other DNA"
     /label="source Cloning vector pUC19"
---
     misc_feature        4724..4727
     /created_by="User"
```

```
/modified_by="User"
/label="SmiI5"
/note="Geneious type: restriction_site"
```

```
misc_feature        4724..4727
/label="SmiI5 add second (concatenated sequence 6)"
/note="Geneious type: Concatenated sequence"
```

```
misc_feature        4728..4767
/gene="lacZalpha"
/note="multiple cloning site (EcoRI-HindIII)"
/label="lacZalpha misc"
```

```
CDS                 complement(4728..4784)
/gene="lacZalpha"
/codon_start=1
/product="beta-galactosidase alpha fragment"
/translation="MTMITPSLHACRSTLEDPRVPSSNSLAVVLQRRDWENPGVTQLNRLAAHPPFASWRNSEEARTD
RPSQQLRSLNGEWRLMRYFLLTHLCGISHRIWCTLSTICSDAA"
/label="lacZalpha CDS"
```

```
gene                complement(4728..4784)
/gene="lacZalpha"
/label="lacZalpha gene"
```

```
source              4728..7001
/organism="Cloning vector pUC19"
/mol_type="other DNA"
/label="source Cloning vector pUC19"
```

```
misc_feature        4728..4731
/created_by="User"
/modified_by="User"
/label="SmiI3"
/note="Geneious type: restriction_site"
```

```
misc_feature        4728..7001
/label="pUC19 3' (concatenated sequence 7)"
/note="Geneious type: Concatenated sequence"
```

```
primer_bind         4728..4753
/created_by="User"
/label="pUC19productionF-Smi"
```

```
misc_feature        4732..4737
/Recognition_pattern="G^GATCC"
/annotation_group="GGATCC"
/label="BamHI"
/note="Geneious type: restriction site"
```

```
misc_feature        4738..4743
/Recognition_pattern="T^CTAGA"
```

```
     /annotation_group="TCTAGA"
     /label="XbaI"
     /note="Geneious type: restriction site"
misc_feature        4744..4749
     /Recognition_pattern="G^TCGAC"
     /annotation_group="GTCGAC"
     /label="SalI"
     /note="Geneious type: restriction site"
misc_feature        4750..4755
     /Recognition_pattern="CTGCA^G"
     /annotation_group="CTGCAG"
     /label="PstI"
     /note="Geneious type: restriction site"
misc_feature        4762..4767
     /Recognition_pattern="A^AGCTT"
     /annotation_group="AAGCTT"
     /label="HindIII"
     /note="Geneious type: restriction site"
misc_feature        complement(4793..4816)
     /created_by="User"
     /label="M13R"
     /note="Geneious type: primer_bind_reverse"
-10_signal          complement(4829..4834)
     /note="Plac promoter (drives lacZalpha;
     counter-clockwise); TATGTT"
     /label="-10 signal"
-35_signal          complement(4853..4858)
     /note="Plac promoter (drives lacZalpha;
     counter-clockwise); TTTACA"
     /label="-35 signal"
protein_bind        complement(4878..4890)
     /note="CAP protein binding site"
     /bound_moiety="catabolite activator protein (CAP)"
     /label="CAP protein binding site protein bind"
rep_origin          complement(5182..5770)
     /note="pMB1 origin of replication (counter-clockwise)
     (RNAII -35 to RNA/DNA switch point)"
     /label="rep origin"
misc_RNA            complement(5182..5734)
     /note="RNAII transcript (complementary strand)"
     /label="RNA"
-35_signal          5588..5593
```

/note="RNAI promoter (clockwise); TTGAAG"
/label="RNAI promoter (clockwise); TTGAAG -35 signal"

-10_signal 5610..5615
/note="RNAI promoter (clockwise); GCTACA"
/label="RNAI promoter (clockwise); GCTACA -10 signal"

misc_RNA 5624..5731
/note="RNAI transcript"
/label="RNAI transcript"

-10_signal complement(5744..5749)
/note="RNAII promoter (counter-clockwise); CGTAAT"
/label="-10 signal"

-35_signal complement(5765..5770)
/note="RNAII promoter (counter-clockwise); TTGAGA"
/label="-35 signal"

CDS complement(5941..6801)
/gene="bla"
/note="ampR (confers resistance to ampicillin)"
/codon_start=1
/product="beta-lactamase"
/translation="MSIQHFRVALIPFFAAFCLPVFAHPETLVKVKDAEDQLGARVGYIELDLNSGKILESFRPEERF
PMMSTFKVLLCGAVLSRIDAGQEQLGRRIHYSQNDLVEYSPVTEKHLTDGMTVRELCSAAITMSDNTAANLLLTTIGG
PKELTAFLHNMGDHVTRLDRWEPELNEAIPNDERDTTMPVAMATTLRKLLTGELLTLASRQQLIDWMEADKVAGPLL
RSALPAGWFIADKSGAGERGSRGIIAALGPDGKPSRIVVIYTTGSQATMDERNRQIAEIGASLIKHW"
/label="bla CDS"

gene complement(5941..6801)
/gene="bla"
/label="bla gene"

misc_feature 6128..6138
/Recognition_pattern="GCCNNNN^NGGC"
/annotation_group="GCCNNNNNGGC"
/label="BglI"
/note="Geneious type: restriction site"

sig_peptide complement(6733..6801)
/gene="bla"
/note="required for secretion to the periplasm; cleaved
off to form the mature beta-lactamase protein."
/label="bla signal peptide"

-10_signal complement(6845..6850)
/note="bla promoter (counter-clockwise); GAGACA"
/label="-10 signal"

-35_signal complement(6866..6871)
/note="bla promoter (counter-clockwise); TTCAAA"

/label="-35 signal"

```
        10        20        30        40
TCGCGCGTTTCGGTGATGACGGTGAAAACCTCTGACACAT 40
GCAGCTCCCGGAGACGGTCACAGCTTGTCTGTAAGCGGAT 80
GCCGGGAGCAGACAAGCCCGTCAGGGCGCGTCAGCGGGTG 120
TTGGCGGGTGTCGGGGCTGGCTTAACTATGCGGCATCAGA 160
GCAGATTGTACTGAGAGTGCACCATATGCGGTGTGAAATA 200
       210       220       230       240
CCGCACAGATGCGTAAGGAGAAAATACCGCATCAGGCGCC 240
ATTCGCCATTCAGGCTGCGCAACTGTTGGGAAGGGCGATC 280
GGTGCGGGCCTCTTCGCTATTACGCCAGCTGGCGAAAGGG 320
GGATGTGCTGCAAGGCGATTAAGTTGGGTAACGCCAGGGT 360
TTTCCCAGTCACGACGTTGTAAAACGACGGCCAGTGAATT 400
       410       420       430       440
CGAGCTCGGTAATTTAAATGCGGATTGCCGCGGTCTTATA 440
AATTTGAATAACTCGCAGCGGTGGGGACAGCGTTTGTGCG 480
TCGTTTGTATAAAGGGAGCGTTTGGTCTCGGAAAACAGCC 520
TCATTGTCATGAGCAGACCGGACTTTTGCTCGACGCTCTG 560
ACACGGGAACGCTGGAAAGCGAACTCAGTGATGCACTGCA 600
       610       620       630       640
TGTAACAAATGCTCGCTTTCGGGCTGGTGCTAATTCTCGT 640
TAGATGCATCTGGTCCCTGCAGCTGTGTGCTGTAAAATGT 680
GCGCATACAGACATCGCGGCGTATCCAGTAGACTTCAGTA 720
TTTCCCTCGAGTCGTGCGGGGTTTTGTGTGAGCGTCGATC 760
GAGGCCATTGTTTCATAGCACTGGGGTTTCCCGAGGTTTT 800
       810       820       830       840
CTGGATTCCCATCTGTTTTTTTTAGATTTGTGGCATGTAT 840
GTGCTCTAAACGTCTGCGGATTTGTAAACCTTCAGTGTGG 880
ACTCGTGAGTTATTTCCTTTTGCTCGAGTCCCCTGCTCGA 920
TTAACCCATTGGACATTGTCCCCCGTAAGTTCGAATCGTC 960
CGCAGTTTTTTTCTCGAAAAGTCTGTGCAAGTCGCTCTAT 1000
      1010      1020      1030      1040
TCTGCCTCAGTTGAACACACGTGAAGCGGTTCCCTATCTG 1040
CTCAGACTTACTAATTTTTGAAGAGCAAGAGATAAAATGT 1080
GAATCTATGGCTTCCTGGTTTCTGCACTTCCCTGTCGCTA 1120
CCCTTTTTCGAGTTCCCTTTGTTATTTGCCAATGAAATAA 1160
TTCGTTCACTGTGTCATATACGTTCTACGAATCGTGCCGG 1200
      1210      1220      1230      1240
GGGAGTGCGCGTAAACTTACCCCTTTCTGGGAGGACACAC 1240
AAAAAAGAACCCCACGCCTTTGTTGTATGGACTCCCTCAT 1280
ACGGTCTTGCTAGCATGTCCTCAACAGTAAGGAATCTGGT 1320
AAATTGTTGTCCTTCCTCCTGTCTTTTTTCCACGAAAAGT 1360
TCATCATCTGCGGTTGGCCTTGCAATTTCCCCTCTGGCTT 1400
```

```
        1410      1420      1430      1440
TGCTCCCCGGATGAATCCCCGCATGATGGCATAGTCGAG 1440
ACACCCGAAACAGTCGTTCTGAAGTGTCATTTCTTCTTTT 1480
GTCGCCACATGCGTCTCTGCTTGGTACCTTGATCAGCACG 1520
AAACCTTGCATTCAAACCCGCCCGCGGAAGATCCGATCTT 1560
GCTGCTGTTCGCAGTCCCAGTAGCGTCCTGTCGGCCGCGC 1600
        1610      1620      1630      1640
CGTCTCTGTTGGTGGGCAGCCGCTACACCTGTTATCTGAC 1640
TGCCGTGCGCGAAAATGACGCCATTTTTGGGAAAATCGGG 1680
GAACTTCATTCTTTAAAAGTATGCGGAGGTTTCCTTTTTC 1720
TTCTGTTCGTTTCTTTTTCTCGGGTTTGATAACCGTGTTC 1760
GATGTAAGCACTTTCCGTCTCTCCTCCGTGCTTTGTTCGA 1800
        1810      1820      1830      1840
CATCGAGACCAGGTGTGCAGATCCTTCGCTTGTCGATCCG 1840
GAGACGCGTGTCTCGTAGAACCTTTTCATTTTACCACACG 1880
GCAGTGCGGAGCACTGCTCTGAGTGCAGCAGGGACGGGTG 1920
AAGTTTCGCTTTAGTAGTGCGTTTCTGCTCTACGGGGCGT 1960
TGTCAGATCCAGCAAAATGGCGTCCAAACCCATTGAAGAC 2000
        2010      2020      2030      2040
TACGGCAAGGGCAAGGGCCGTATTGAGCCCATGTATATCC 2040
CCGACAACACCTTCTACAACGCTGATGACTTTCTTGTGCC 2080
CCCCCACTGCAAGCCCTACATTGACAAAATCCTCCTCCCT 2120
GGTGGATTGGTCAAGGACAGAGTTGAGAAGTTGGCGTATG 2160
ACATCCACAGAACTTACTTCGGCGAGGAGTTGCACATCAT 2200
        2210      2220      2230      2240
TTGCATCCTGAAAGGCTCTCGCGGCTTCTTCAACCTTCTG 2240
ATCGACTACCTTGCCACCATACAGAAGTACAGTGGTCGTG 2280
AGTCCAGCGTGCCCCCCTTCTTCGAGCACTATGTCCGCCT 2320
GAAGTCCTACCAGAACGACAACAGCACAGGCCAGCTCACC 2360
GTCTTGAGCGACGACTTGTCAATCTTTCGCGACAAGCACG 2400
        2410      2420      2430      2440
TTCTGATTGTTGAGGACATCGTCGACACCGGTTTCACCCT 2440
CACCGAGTTCGGTGAGCGCCTGAAAGCCGTCGGTCCCAAG 2480
TCGATGAGAATCGCCACCCTCGTCGAGAAGCGCACAGATC 2520
GCTCCAACAGCTTGAAGGGCGACTTCGTCGGCTTCAGCAT 2560
TGAAGACGTCTGGATCGTTGGTTGCTGCTACGACTTCAAC 2600
        2610      2620      2630      2640
GAGATGTTCCGCGACTTCGACCACGTCGCCGTCCTGAGCG 2640
ACGCCGCTCGCAAAAAGTTCGAGAAGTAAACCCTGCATAG 2680
CCCTACAGAAGCTGCCCGTCTCTCGTTTTCCTCTCTTTTC 2720
GGAGGGATCAGGGAGAGTGCCTCGGGTCGGAGAGAGCTGA 2760
CGAGGGGGTGCCAGAGACCCCTGTGTCCTTTATCGAAGAA 2800
```

```
        2810      2820      2830      2840
AAGGGATGACTCTTCATGTGGCATTTCACACAGTCTCACC 2840
TCGCCTTGTTTTCTTTTTGTCAATCAGAACGAAAGCGAGT 2880
TGCGGGTGACGCAGATGTGCGTGTATCCACTCGTGAATGC 2920
GTTATCGTTCTGTATGCCGCTAGAGTGCTGGACTGTTGCT 2960
GTCTGCCCACGACAGCAGACAACTTTCCTTCTATGCACTT 3000
        3010      3020      3030      3040
GCAGGATGGTGCAGCGCAAACGACGGAGAGAAAGGAGCAC 3040
CCTCTCAGTTTCCCTACGATGTGCTGTCAGTTTCGACTCT 3080
TCACCGCGAACGATTGGCGATACGTCTCTGTTGACTTGTT 3120
AGGCTCCGACCACGAAGCTCCCTTAACTAAATAAGCCGCG 3160
ACACCTAAGTGTACACCATTTGCAGATCGATAATCTGCGA 3200
        3210      3220      3230      3240
CCGCTGAATCCGTCCAGATCAGGAAAACCGCACCACCTAA 3240
GTGTAAACCTTGTTTAGGTCGATAAAATGCTACCAACCCC 3280
CACCCACAATCGAGCCTTGAGCGTTTCTGCGCACGCGTTG 3320
GCCTACGTGACTTGCTGATGCCTGCCTCTGGCCATTCATG 3360
CCAGTCAGTGCGCATAAAAATGTGGACACAGTCGGTTGAC 3400
        3410      3420      3430      3440
AAGTGTTCTGGCAGGCTACAGTGACACCGCGGTGGAGGTA 3440
CCGCAAGACCGCACACAGGAACTGTGAGACGGAACAAAAC 3480
GGATTTTTGACGACACGTCTCGGTGTCCCCTACCCACTTC 3520
AGTCTACTTTCAGTTTCGTCTGCTCTCCCACGCAATTCCT 3560
CGCGCGCTTGGGATCTGGGGAATGTGTTTCTTCCGATGCC 3600
        3610      3620      3630      3640
CATAGGCGAAACGTCTTCTTCCGAGTACAGAGGCCACAAG 3640
GGTAGTGCCGTTTTTAGAAACAGTATTCACATGCTAGAGG 3680
GTTTGCGTCTTTTGCTGTGCCTGACGCAATGGAAAGGGAA 3720
ATAAATCTACGGTAAACGAGGAGTCGCATCTGCTAGTTGG 3760
ATCCGGCTGGCATCCACAGCGGCGGGGGCGTGGGTCCGTG 3800
        3810      3820      3830      3840
GAAACAGGCAAAATCCGAGTGAATATAATACCGATGCGTG 3840
GGCGGAAATGTGAGGGCCTTGCGGGGAGGACTGGGACAAC 3880
GTTCGGCTGAACTGCGCTTGAGAGAAAGGAGTGGGTGAAG 3920
CTCTGACGGGGAGATGCCTCGCGTTTTCGTCCGTTTGAGG 3960
GCACCGCAGATTGGGGTCCCAGGCGACCCGTCGATACACC 4000
        4010      4020      4030      4040
CACAGAATGTTTTCGGTAAAACCATCGTTCAGGAAGGATT 4040
CGCTTTTGCAAAACCGTGAAAGACATCAACCTGGTCTTTC 4080
TTTGGAGGGCAGAACCAAAGCGAGGCACGAATGGTTTGTT 4120
TCTGGGCCACACTGATAGTTAAATTCTCTTCCCCATACTT 4160
TCGCATTCTGTTTCCCATTTGGACTGCAAGCTCAGAGATA 4200
```

```
        4210      4220      4230      4240
GCTTAGAACCGTACCGATTGGGATTGCAGTATTAACGGGA 4240
GAAAAGTATTTTCGGCATCAAGCTACTTTCCTCCCCGTCT 4280
TTACTCGAGGAGAAAAGGCGCAGTGTTGGTTCATACGTAT 4320
ATATACGCGCTATCGCTGTTGTGGAAAAAGGGGTCAGCAG 4360
AGAGATTGTGGTGTACGCAGGAGTAACCATAAGGTAACTG 4400
        4410      4420      4430      4440
CCATGTTGGGCTCTTATGGTACTCAGTGCATCCCGCGGCT 4440
GGGGGCTGAAATACAAAGAAATGCTGTTCCCTCCTGCACA 4480
AGACGCATCTGAATACTAACCGCCAGACATGAATATACTG 4520
CTTCTGTGAATGCCAAAGGTCTCGCGTGTAGAATTTCTAC 4560
GCGGGGCAGTCGCCTAGCACCTGCTCACTTTTAGTCAGAT 4600
        4610      4620      4630      4640
TCATCGCAGCGGGAGAAACGCACAGTGCAGCTCGTTTGAA 4640
AGCGGGATAATATGCGAGGCCCGCAAACTATTTCTCACTT 4680
CAAGCGAACGTCGACAGTACAGGCGTGTTTGGCTTTGAGC 4720
ACGATTTAAATGGATCCTCTAGAGTCGACCTGCAGGCATG 4760
CAAGCTTGGCGTAATCATGGTCATAGCTGTTTCCTGTGTG 4800
        4810      4820      4830      4840
AAATTGTTATCCGCTCACAATTCCACACAACATACGAGCC 4840
GGAAGCATAAAGTGTAAAGCCTGGGGTGCCTAATGAGTGA 4880
GCTAACTCACATTAATTGCGTTGCGCTCACTGCCCGCTTT 4920
CCAGTCGGGAAACCTGTCGTGCCAGCTGCATTAATGAATC 4960
GGCCAACGCGCGGGGAGAGGCGGTTTGCGTATTGGGCGCT 5000
        5010      5020      5030      5040
CTTCCGCTTCCTCGCTCACTGACTCGCTGCGCTCGGTCGT 5040
TCGGCTGCGGCGAGCGGTATCAGCTCACTCAAAGGCGGTA 5080
ATACGGTTATCCACAGAATCAGGGGATAACGCAGGAAAGA 5120
ACATGTGAGCAAAAGGCCAGCAAAAGGCCAGGAACCGTAA 5160
AAAGGCCGCGTTGCTGGCGTTTTTCCATAGGCTCCGCCCC 5200
        5210      5220      5230      5240
CCTGACGAGCATCACAAAAATCGACGCTCAAGTCAGAGGT 5240
GGCGAAACCCGACAGGACTATAAAGATACCAGGCGTTTCC 5280
CCCTGGAAGCTCCCTCGTGCGCTCTCCTGTTCCGACCCTG 5320
CCGCTTACCGGATACCTGTCCGCCTTTCTCCCTTCGGGAA 5360
GCGTGGCGCTTTCTCATAGCTCACGCTGTAGGTATCTCAG 5400
        5410      5420      5430      5440
TTCGGTGTAGGTCGTTCGCTCCAAGCTGGGCTGTGTGCAC 5440
GAACCCCCCGTTCAGCCCGACCGCTGCGCCTTATCCGGTA 5480
ACTATCGTCTTGAGTCCAACCCGGTAAGACACGACTTATC 5520
GCCACTGGCAGCAGCCACTGGTAACAGGATTAGCAGAGCG 5560
AGGTATGTAGGCGGTGCTACAGAGTTCTTGAAGTGGTGGC 5600
```

5610 5620 5630 5640

CTAACTACGGCTACACTAGAAGAACAGTATTTGGTATCTG 5640
CGCTCTGCTGAAGCCAGTTACCTTCGGAAAAAGAGTTGGT 5680
AGCTCTTGATCCGGCAAACAAACCACCGCTGGTAGCGGTG 5720
GTTTTTTTGTTTGCAAGCAGCAGATTACGCGCAGAAAAAA 5760
AGGATCTCAAGAAGATCCTTTGATCTTTTCTACGGGGTCT 5800

5810 5820 5830 5840

GACGCTCAGTGGAACGAAAACTCACGTTAAGGGATTTTGG 5840
TCATGAGATTATCAAAAAGGATCTTCACCTAGATCCTTTT 5880
AAATTAAAAATGAAGTTTTAAATCAATCTAAAGTATATAT 5920
GAGTAAACTTGGTCTGACAGTTACCAATGCTTAATCAGTG 5960
AGGCACCTATCTCAGCGATCTGTCTATTTCGTTCATCCAT 6000

6010 6020 6030 6040

AGTTGCCTGACTCCCCGTCGTGTAGATAACTACGATACGG 6040
GAGGGCTTACCATCTGGCCCCAGTGCTGCAATGATACCGC 6080
GAGACCCACGCTCACCGGCTCCAGATTTATCAGCAATAAA 6120
CCAGCCAGCCGGAAGGGCCGAGCGCAGAAGTGGTCCTGCA 6160
ACTTTATCCGCCTCCATCCAGTCTATTAATTGTTGCCGGG 6200

6210 6220 6230 6240

AAGCTAGAGTAAGTAGTTCGCCAGTTAATAGTTTGCGCAA 6240
CGTTGTTGCCATTGCTACAGGCATCGTGGTGTCACGCTCG 6280
TCGTTTGGTATGGCTTCATTCAGCTCCGGTTCCCAACGAT 6320
CAAGGCGAGTTACATGATCCCCCATGTTGTGCAAAAAAGC 6360
GGTTAGCTCCTTCGGTCCTCCGATCGTTGTCAGAAGTAAG 6400

6410 6420 6430 6440

TTGGCCGCAGTGTTATCACTCATGGTTATGGCAGCACTGC 6440
ATAATTCTCTTACTGTCATGCCATCCGTAAGATGCTTTTC 6480
TGTGACTGGTGAGTACTCAACCAAGTCATTCTGAGAATAG 6520
TGTATGCGGCGACCGAGTTGCTCTTGCCCGGCGTCAATAC 6560
GGGATAATACCGCGCCACATAGCAGAACTTTAAAAGTGCT 6600

6610 6620 6630 6640

CATCATTGGAAAACGTTCTTCGGGGCGAAAACTCTCAAGG 6640
ATCTTACCGCTGTTGAGATCCAGTTCGATGTAACCCACTC 6680
GTGCACCCAACTGATCTTCAGCATCTTTTACTTTCACCAG 6720
CGTTTCTGGGTGAGCAAAAACAGGAAGGCAAAATGCCGCA 6760
AAAAAGGGAATAAGGGCGACACGGAAATGTTGAATACTCA 6800

6810 6820 6830 6840

TACTCTTCCTTTTTCAATATTATTGAAGCATTTATCAGGG 6840
TTATTGTCTCATGAGCGGATACATATTTGAATGTATTTAG 6880
AAAAATAAACAAATAGGGGTTCCGCGCACATTTCCCCGAA 6920
AAGTGCCACCTGACGTCTAAGAAACCATTATTATCATGAC 6960
ATTAACCTATAAAAATAGGCGTATCACGAGGCCCTTTCGT 7000

7010 7020 7030 7040

C 7001

```
LOCUS       pUC19_T2KO_vecto        6691 bp    DNA
 circular UNA 17-DEC-2012
DEFINITION  Concatenation of 5 sequences.
ACCESSION   urn.local...1317875701146.8
VERSION     urn.local...1317875701146.8
KEYWORDS    .
SOURCE
  ORGANISM
            .



ORIGIN
Features
     source             1..415
     /organism="Cloning vector pUC19"
     /mol_type="other DNA"
     /label="source Cloning vector pUC19"
     CDS                complement(146..415)
     /gene="lacZalpha"
     /codon_start=1
     /product="beta-galactosidase alpha fragment"
     /translation="MTMITPSLHACRSTLEDPRVPSSNSLAVVLQRRDWENPGVTQLNRLAAHPPFASWRNSEEARTDRPSQQLRSLNGEWRLMRYFLLTHLCGISHR
     STICSDAA"
     /label="lacZalpha CDS"
     gene               complement(146..415)
     /gene="lacZalpha"
     /label="lacZalpha gene"
     misc_feature       183..188
     /Recognition_pattern="CA^TATG"
     /annotation_group="CATATG"
     /label="NdeI"
     /note="Geneious type: restriction site"
     misc_feature       245..255
     /Recognition_pattern="GCCNNNN^NGGC"
     /annotation_group="GCCNNNNNGGC"
     /label="BglI"
     /note="Geneious type: restriction site"
     misc_feature       complement(391..415)
     /created_by="User"
     /label="pUC19productionR-Smi"
     /note="Geneious type: primer_bind_reverse"
     misc_feature       396..415
     /gene="lacZalpha"
     /note="multiple cloning site (EcoRI-HindIII)"
     /label="lacZalpha misc"
     misc_feature       396..401
     /Recognition_pattern="G^AATTC"
     /annotation_group="GAATTC"
     /label="EcoRI"
     /note="Geneious type: restriction site"
     primer_bind        401..440
```

[illegible]
/note="multiple cloning site (EcoRI-HindIII)"
/label="lacZalpha misc"

---

misc_feature 396..401
/Recognition_pattern="G^AATTC"
/annotation_group="GAATTC"
/label="EcoRI"
/note="Geneious type: restriction site"

---

primer_bind 401..440
/created_by="User"
/label="T2ko-5'F w/adp"

---

misc_feature 402..407
/Recognition_pattern="GAGCT^C"
/annotation_group="GAGCTC"
/label="SacI"
/note="Geneious type: restriction site"

---

misc_feature 412..415
/created_by="User"
/modified_by="User"
/label="SmiI5"
/note="Geneious type: restriction_site"

---

misc_feature 416..419
/created_by="User"
/label="Smi3"
/note="Geneious type: restriction_site"

---

misc_recomb 420..1336
/created_by="User"
/label="5'Recomb"

---

primer_bind 420..440
/created_by="User"
/label="T2ko-5'F"

---

misc_feature complement(1315..1351)
/created_by="User"
/modified_by="User"
/label="T2ko-5'R w/adp"
/note="Geneious type: primer_bind_reverse"

---

primer_bind complement(1315..1336)
/created_by="User"
/label="T2ko-5'R"

---

misc_feature 1337..1342
/created_by="User"
/label="KpnI"
/note="Geneious type: restriction_site"

```
primer_bind         1337..1363
/created_by="User"
/label="HXGPRT-KpnIF"
```

```
gene                1343..3272
/created_by="User"
/label="DHFR-HXGPRT"
```

```
misc_feature        complement(1556..1578)
/created_by="User"
/label="5ex2R"
/note="Geneious type: primer_bind_reverse"
```

```
misc_feature        1805..1807
/Original_Bases
/label
/note="Geneious type: Editing History Insertion"
```

```
Selectable_Mark     1811..2503
/label="HXGPRT"
```

```
primer              1818..1837
/note="Oligo predicted by CLC primer designer"
/note="Oligo type: primer"
/note="Melting temp.: 56.07"
/label="HXGPRT detection"
```

```
primer              complement(2533..2554)
/note="Oligo predicted by CLC primer designer"
/note="Oligo type: primer"
/note="Melting temp.: 54.02"
/label="HXGPRT detection"
```

```
misc_feature        complement(3256..3276)
/created_by="User"
/label="HXGPRT-KpnIR"
/note="Geneious type: primer_bind_reverse"
```

```
primer_bind         3262..3297
/created_by="User"
/label="T2ko-3'F w/adp"
```

```
misc_feature        3271..3276
/created_by="User"
/modified_by="User"
/label="KpnI"
/note="Geneious type: restriction_site"
```

```
misc_recomb         3277..4413
/created_by="User"
/modified_by="User"
/label="3'Recomb"
```

```
primer_bind          3277..3297
/created_by="User"
/label="T2ko-3'F"
```

```
gene                 3277..3840
/Source="annotation"
/Note="UDP-N-acetyl-D-galactosamine:polypeptide
N-acetylgalactosaminyltransferase T2"
/soTerm="protein_coding"
/product="UDP-N-acetyl-D-galactosamine:polypeptide
N-acetylgalactosaminyltransferase T2"
/taxon="Toxoplasma gondii ME49"
/isDeprecated=0
/isPseudo=0
/label="TGME49_058770"
```

```
UTR                  3277..3840
/Source="annotation"
/isDeprecated=0
/label="UTR"
```

```
misc_feature         complement(4393..4432)
/created_by="User"
/label="T2ko-3'R w/adpt"
/note="Geneious type: primer_bind_reverse"
```

```
primer_bind          complement(4393..4413)
/created_by="User"
/label="T2ko-3'R"
```

```
misc_feature         4414..4417
/created_by="User"
/label="Smi5"
/note="Geneious type: restriction_site"
```

```
misc_feature         4418..4457
/gene="lacZalpha"
/note="multiple cloning site (EcoRI-HindIII)"
/label="lacZalpha misc"
```

```
source               4418..6691
/organism="Cloning vector pUC19"
/mol_type="other DNA"
/label="source Cloning vector pUC19"
```

```
CDS                  complement(4418..4474)
/gene="lacZalpha"
/codon_start=1
/product="beta-galactosidase alpha fragment"
/translation="MTMITPSLHACRSTLEDPRVPSSNSLAVVLQRRDWENPGVTQLNRLAAHPPFASWRNSEEARTD
```

```
RPSQQLRSLNGEWRLMRYFLLTHLCGISHRIWCTLSTICSDAA"
/label="lacZalpha CDS"
gene                complement(4418..4474)
/gene="lacZalpha"
/label="lacZalpha gene"
misc_feature        4418..4421
/created_by="User"
/modified_by="User"
/label="SmiI3"
/note="Geneious type: restriction_site"
primer_bind         4418..4443
/created_by="User"
/label="pUC19productionF-Smi"
misc_feature        4422..4427
/Recognition_pattern="G^GATCC"
/annotation_group="GGATCC"
/label="BamHI"
/note="Geneious type: restriction site"
misc_feature        4428..4433
/Recognition_pattern="T^CTAGA"
/annotation_group="TCTAGA"
/label="XbaI"
/note="Geneious type: restriction site"
misc_feature        4434..4439
/Recognition_pattern="G^TCGAC"
/annotation_group="GTCGAC"
/label="SalI"
/note="Geneious type: restriction site"
misc_feature        4440..4445
/Recognition_pattern="CTGCA^G"
/annotation_group="CTGCAG"
/label="PstI"
/note="Geneious type: restriction site"
misc_feature        4452..4457
/Recognition_pattern="A^AGCTT"
/annotation_group="AAGCTT"
/label="HindIII"
/note="Geneious type: restriction site"
-10_signal          complement(4519..4524)
/note="Plac promoter (drives lacZalpha;
counter-clockwise); TATGTT"
/label="-10 signal"
```

```
-35_signal          complement(4543..4548)
/note="Plac promoter (drives lacZalpha;
counter-clockwise); TTTACA"
/label="-35 signal"
```

```
protein_bind        complement(4568..4580)
/note="CAP protein binding site"
/bound_moiety="catabolite activator protein (CAP)"
/label="CAP protein binding site protein bind"
```

```
rep_origin          complement(4872..5460)
/note="pMB1 origin of replication (counter-clockwise)
(RNAII -35 to RNA/DNA switch point)"
/label="rep origin"
```

```
misc_RNA            complement(4872..5424)
/note="RNAII transcript (complementary strand)"
/label="RNA"
```

```
-35_signal          5278..5283
/note="RNAI promoter (clockwise); TTGAAG"
/label="RNAI promoter (clockwise); TTGAAG -35 signal"
```

```
-10_signal          5300..5305
/note="RNAI promoter (clockwise); GCTACA"
/label="RNAI promoter (clockwise); GCTACA -10 signal"
```

```
misc_RNA            5314..5421
/note="RNAI transcript"
/label="RNAI transcript"
```

```
-10_signal          complement(5434..5439)
/note="RNAII promoter (counter-clockwise); CGTAAT"
/label="-10 signal"
```

```
-35_signal          complement(5455..5460)
/note="RNAII promoter (counter-clockwise); TTGAGA"
/label="-35 signal"
```

```
CDS                 complement(5631..6491)
/gene="bla"
/note="ampR (confers resistance to ampicillin)"
/codon_start=1
/product="beta-lactamase"
/translation="MSIQHFRVALIPFFAAFCLPVFAHPETLVKVKDAEDQLGARVGYIELDLNSGKILESFRPEERF
PMMSTFKVLLCGAVLSRIDAGQEQLGRRIHYSQNDLVEYSPVTEKHLTDGMTVRELCSAAITMSDNTAANLLLTTIGG
PKELTAFLHNMGDHVTRLDRWEPELNEAIPNDERDTTMPVAMATTLRKLLTGELLTLASRQQLIDWMEADKVAGPLL
RSALPAGWFIADKSGAGERGSRGIIAALGPDGKPSRIVVIYTTGSQATMDERNRQIAEIGASLIKHW"
/label="bla CDS"
```

```
gene                complement(5631..6491)
/gene="bla"
```

```
     /label="bla gene"
     misc_feature        5818..5828
     /Recognition_pattern="GCCNNNN^NGGC"
     /annotation_group="GCCNNNNNGGC"
     /label="BglI"
     /note="Geneious type: restriction site"
     sig_peptide         complement(6423..6491)
     /gene="bla"
     /note="required for secretion to the periplasm; cleaved
     off to form the mature beta-lactamase protein."
     /label="bla signal peptide"
     -10_signal          complement(6535..6540)
     /note="bla promoter (counter-clockwise); GAGACA"
     /label="-10 signal"
     -35_signal          complement(6556..6561)
     /note="bla promoter (counter-clockwise); TTCAAA"
     /label="-35 signal"

        10        20        30        40
tcgcgcgtttcggtgatgacggtgaaaacctctgacacat 40
gcagctcccggagacggtcacagcttgtctgtaagcggat 80
gccgggagcagacaagcccgtcagggcgcgtcagcgggtg 120
ttggcgggtgtcggggctggcttaactatgcggcatcaga 160
gcagattgtactgagagtgcaccatatgcggtgtgaaata 200
       210       220       230       240
ccgcacagatgcgtaaggagaaaataccgcatcaggcgcc 240
attcgccattcaggctgcgcaactgttgggaagggcgatc 280
ggtgcgggcctcttcgctattacgccagctggcgaaaggg 320
ggatgtgctgcaaggcgattaagttgggtaacgccagggt 360
tttcccagtcacgacgttgtaaaacgacggccagtgaatt 400
       410       420       430       440
cgagctcggtaatttaaatactctcctcgctgcggttctt 440
tccttcttaagagcatccgttgtcgcgtcctgaccctctc 480
ccccccaccctccgcgagaacggggaaaacctgaaccct 520
ctgagaaatgaagcgcctctcgtcagacgcggcgactcgc 560
cacggactttccagaaaagtcaacgcgtggaactgagggc 600
       610       620       630       640
cgctctcgaaggctctctgccctgcaccttgagcccagat 640
gtattgcgaaggaaggcctgccgcactgcctcgtctcgac 680
aaggttgtctcctgcgccgccgggactcgactaagaagaa 720
tcttcgcaaacgcttcacactcctcatgtgctcgagttcg 760
gtcctcgttcccaggctcctcgttcctctcttcccctgtc 800
```

```
        810       820       830       840
tccgcgggctcttcgcgtccaccgaaaagcggtcttcctc 840
ctcaagaaaacggatcgcctttctgtggggaacatccgtg 880
ttctcccgctcggcgtgtgaactaggaagctcgcagcgtc 920
tcaacttcttaaatctatttacattttaagtgtaatcata 960
tatgcatatttgtgtttaaataaggtaaacataactgtat 1000
       1010      1020      1030      1040
acagatttatactataaccataagcataaatacacatata 1040
gatatatgtatacgtatatgtctcagcgccgttgaatccc 1080
cttcctggtgtccgtcctgggaactgtcgaacttctcttg 1120
gtgggggattgtgggtcggcggcgcctacgctctcaactc 1160
gcttctgtcctcttctcggttcttctggagttctgatggt 1200
       1210      1220      1230      1240
tccacaaggactccccgttaccatttcgtccgctctttga 1240
cttcgttcacttcctctctgctgggcaagcgatacgtgaa 1280
ggccctggcctaggctgccattgttctctctcctcgttcg 1320
ttcccgaggagccttaggtaccttgatcagcacgaaacct 1360
tgcattcaaacccgcccgcggaagatccgatcttgctgct 1400
       1410      1420      1430      1440
gttcgcagtcccagtagcgtcctgtcggccgcgccgtctc 1440
tgttggtgggcagccgctacacctgttatctgactgccgt 1480
gcgcgaaaatgacgccattttgggaaaatcggggaactt 1520
cattctttaaaagtatgcggaggtttcctttttcttctgt 1560
tcgtttctttttctcgggtttgataaccgtgttcgatgta 1600
       1610      1620      1630      1640
agcactttccgtctctcctccgtgctttgttcgacatcga 1640
gaccaggtgtgcagatccttcgcttgtcgatccggagacg 1680
cgtgtctcgtagaacctttcatttaccacacggcagtg 1720
cggagcactgctctgagtgcagcagggacgggtgaagttt 1760
cgctttagtagtgcgtttctgctctacggggcgttgtcag 1800
       1810      1820      1830      1840
atccagcaaaatggcgtccaaacccattgaagactacggc 1840
aagggcaagggccgtattgagcccatgtatatccccgaca 1880
acaccttctacaacgctgatgactttcttgtgcccccca 1920
ctgcaagccctacattgacaaaatcctcctccctggtgga 1960
ttggtcaaggacagagttgagaagttggcgtatgacatcc 2000
       2010      2020      2030      2040
acagaacttacttcggcgaggagttgcacatcatttgcat 2040
cctgaaaggctctcgcggcttcttcaaccttctgatcgac 2080
taccttgccaccatacagaagtacagtggtcgtgagtcca 2120
gcgtgcccccttcttcgagcactatgtccgcctgaagtc 2160
ctaccagaacgacaacagcacaggccagctcaccgtcttg 2200
```

```
        2210      2220      2230      2240
....|....|....|....|....|....|....|....|
agcgacgacttgtcaatctttcgcgacaagcacgttctga 2240
ttgttgaggacatcgtcgacaccggtttcaccctcaccga 2280
gttcggtgagcgcctgaaagccgtcggtcccaagtcgatg 2320
agaatcgccaccctcgtcgagaagcgcacagatcgctcca 2360
acagcttgaagggcgacttcgtcggcttcagcattgaaga 2400
        2410      2420      2430      2440
....|....|....|....|....|....|....|....|
cgtctggatcgttggttgctgctacgacttcaacgagatg 2440
ttccgcgacttcgaccacgtcgccgtcctgagcgacgccg 2480
ctcgcaaaaagttcgagaagtaaaccctgcatagccctac 2520
agaagctgcccgtctctcgtttcctctcttttcggaggg 2560
atcagggagagtgcctcgggtcggagagagctgacgaggg 2600
        2610      2620      2630      2640
....|....|....|....|....|....|....|....|
ggtgccagagacccctgtgtcctttatcgaagaaaaggga 2640
tgactcttcatgtggcatttcacacagtctcacctcgcct 2680
tgttttctttttgtcaatcagaacgaaagcgagttgcggg 2720
tgacgcagatgtgcgtgtatccactcgtgaatgcgttatc 2760
gttctgtatgccgctagagtgctggactgttgctgtctgc 2800
        2810      2820      2830      2840
....|....|....|....|....|....|....|....|
ccacgacagcagacaactttccttctatgcacttgcagga 2840
tggtgcagcgcaaacgacggagagaaaggagcaccctctc 2880
agtttccctacgatgtgctgtcagtttcgactcttcaccg 2920
cgaacgattggcgatacgtctctgttgacttgttaggctc 2960
cgaccacgaagctcccttaactaaataagccgcgacacct 3000
        3010      3020      3030      3040
....|....|....|....|....|....|....|....|
aagtgtacaccatttgcagatcgataatctgcgaccgctg 3040
aatccgtccagatcaggaaaaccgcaccacctaagtgtaa 3080
accttgtttaggtcgataaaatgctaccaacccccaccca 3120
caatcgagccttgagcgtttctgcgcacgcgttggcctac 3160
gtgacttgctgatgcctgcctctggccattcatgccagtc 3200
        3210      3220      3230      3240
....|....|....|....|....|....|....|....|
agtgcgcataaaaatgtggacacagtcggttgacaagtgt 3240
tctggcaggctacagtgacaccgcggtggaggtaccagcg 3280
taagcgagaaacggcacacaaggcggccgtttcagttctc 3320
ccggcgttcgcaacgaaacccccccaaacggggaaaacggc 3360
gacacccaggaggcagaacggctatacgcgacgggggaga 3400
        3410      3420      3430      3440
....|....|....|....|....|....|....|....|
aggcgatccacggaagagcacacccgtcgccgaatgcaga 3440
ctgcaaagagctgtacaaacgaacccggcgccacaaaaac 3480
gagcgtgtagataaacgaaaaaaagcagaatcgcgaagtt 3520
ggctggcgctccaactggcacgagaaatttaggtgtacat 3560
acatatggtccgatgtacagacaccggaggaactccgagt 3600
```

```
        3610      3620      3630      3640
gtctctcggtgtaaattccgcgtcttggtttctcctcgac 3640
tcagaggggaaatagtgtacactgactttttctttcacat 3680
cgaggctcagcgcgcagaacgtctctttttttccgtttgaa 3720
gacacagtcgtcggaagtctcttcttttggtgtagcgata 3760
ctgctttcgctggtgctggcgaagcgccgagccgggttcc 3800
        3810      3820      3830      3840
atcaacagccgtttaaaacagattttgaactaagagaca 3840
cagagccactgcattttgacgatcaagctgcattacatgt 3880
agactttacactggctgtttcagtttccagcgaagcttcc 3920
ttcgtctttttatttgcatacttgttttcgcaacgcgatg 3960
ccgctcagcagcaggcagagcctctctcctggaggcgcag 4000
        4010      4020      4030      4040
tgtttcttttttcgtacactggatcgaccagccgttgcg 4040
gccgcgagtctctttcggagcagttccgcttctttgcccg 4080
tacggggtctctgtgggggcagactcttaggcgaaagcag 4120
cgagtgcggcgactctgtgtagtcgggagacgcgtggaaa 4160
aagcgcggcgcgaaatggatttctggatgcggagaaagag 4200
        4210      4220      4230      4240
ttgaggttgtcgatctttcgcaactggagtgattctggcc 4240
tgcctgttctcacaaaaaacaaatgagcatctatgccggc 4280
ttttgcgacttggagaagtgtactgacatctgttcgaggt 4320
gttggagcgtaaactgttccttctgatctttcggtggagt 4360
ctttcaagctctcagtttctcgctctttgtcgacctttg 4400
        4410      4420      4430      4440
cggaagcgtttgcatttaaatggatcctctagagtcgacc 4440
tgcaggcatgcaagcttggcgtaatcatggtcatagctgt 4480
ttcctgtgtgaaattgttatccgctcacaattccacacaa 4520
catacgagccggaagcataaagtgtaaagcctggggtgcc 4560
taatgagtgagctaactcacattaattgcgttgcgctcac 4600
        4610      4620      4630      4640
tgcccgctttccagtcgggaaacctgtcgtgccagctgca 4640
ttaatgaatcggccaacgcgcggggagaggcggtttgcgt 4680
attgggcgctcttccgcttcctcgctcactgactcgctgc 4720
gctcggtcgttcggctgcggcgagcggtatcagctcactc 4760
aaaggcggtaatacggttatccacagaatcaggggataac 4800
        4810      4820      4830      4840
gcaggaaagaacatgtgagcaaaaggccagcaaaaggcca 4840
ggaaccgtaaaaaggccgcgttgctggcgtttttccatag 4880
gctccgcccccctgacgagcatcacaaaaatcgacgctca 4920
agtcagaggtggcgaaacccgacaggactataaagatacc 4960
aggcgtttccccctggaagctccctcgtgcgctctcctgt 5000
```

```
        5010      5020      5030      5040
tccgaccctgccgcttaccggatacctgtccgcctttctc 5040
ccttcgggaagcgtggcgctttctcatagctcacgctgta 5080
ggtatctcagttcggtgtaggtcgttcgctccaagctggg 5120
ctgtgtgcacgaaccccccgttcagcccgaccgctgcgcc 5160
ttatccggtaactatcgtcttgagtccaacccggtaagac 5200
        5210      5220      5230      5240
acgacttatcgccactggcagcagccactggtaacaggat 5240
tagcagagcgaggtatgtaggcggtgctacagagttcttg 5280
aagtggtggcctaactacggctacactagaagaacagtat 5320
ttggtatctgcgctctgctgaagccagttaccttcggaaa 5360
aagagttggtagctcttgatccggcaaacaaaccaccgct 5400
        5410      5420      5430      5440
ggtagcggtggtttttttgtttgcaagcagcagattacgc 5440
gcagaaaaaaaggatctcaagaagatcctttgatcttttc 5480
tacggggtctgacgctcagtggaacgaaaactcacgttaa 5520
gggattttggtcatgagattatcaaaaaggatcttcacct 5560
agatccttttaaattaaaaatgaagttttaaatcaatcta 5600
        5610      5620      5630      5640
aagtatatatgagtaaacttggtctgacagttaccaatgc 5640
ttaatcagtgaggcacctatctcagcgatctgtctatttc 5680
gttcatccatagttgcctgactccccgtcgtgtagataac 5720
tacgatacgggagggcttaccatctggccccagtgctgca 5760
atgataccgcgagacccacgctcaccggctccagatttat 5800
        5810      5820      5830      5840
cagcaataaaccagccagccggaagggccgagcgcagaag 5840
tggtcctgcaactttatccgcctccatccagtctattaat 5880
tgttgccgggaagctagagtaagtagttcgccagttaata 5920
gtttgcgcaacgttgttgccattgctacaggcatcgtggt 5960
gtcacgctcgtcgtttggtatggcttcattcagctccggt 6000
        6010      6020      6030      6040
tcccaacgatcaaggcgagttacatgatcccccatgttgt 6040
gcaaaaaagcggttagctccttcggtcctccgatcgttgt 6080
cagaagtaagttggccgcagtgttatcactcatggttatg 6120
gcagcactgcataattctcttactgtcatgccatccgtaa 6160
gatgcttttctgtgactggtgagtactcaaccaagtcatt 6200
        6210      6220      6230      6240
ctgagaatagtgtatgcggcgaccgagttgctcttgcccg 6240
gcgtcaatacgggataataccgcgccacatagcagaactt 6280
taaaagtgctcatcattggaaaacgttcttcggggcgaaa 6320
actctcaaggatcttaccgctgttgagatccagttcgatg 6360
taacccactcgtgcacccaactgatcttcagcatctttta 6400
```

```
        6410      6420      6430      6440
ctttcaccagcgtttctgggtgagcaaaaacaggaaggca 6440
aaatgccgcaaaaaagggaataagggcgacacggaaatgt 6480
tgaatactcatactcttcctttttcaatattattgaagca 6520
tttatcagggttattgtctcatgagcggatacatatttga 6560
atgtatttagaaaaataaacaaataggggttccgcgcaca 6600
        6610      6620      6630      6640
tttccccgaaaagtgccacctgacgtctaagaaaccatta 6640
ttatcatgacattaacctataaaaataggcgtatcacgag 6680
gccctttcgtc 6691
```

LOCUS pUC19_T3KO_vecto 6864 bp DNA
circular UNA 17-DEC-2012
DEFINITION Concatenation of 5 sequences.
ACCESSION urn.local...1317877093660.24
VERSION urn.local...1317877093660.24
KEYWORDS .
SOURCE
ORGANISM
.

ORIGIN
Features

source 1..415
/organism="Cloning vector pUC19"
/mol_type="other DNA"
/label="source Cloning vector pUC19"

misc_feature 1..419
/label="pUC19 5' (concatenated sequence 1)"
/note="Geneious type: Concatenated sequence"

CDS complement(146..415)
/gene="lacZalpha"
/codon_start=1
/product="beta-galactosidase alpha fragment"
/translation="MTMITPSLHACRSTLEDPRVPSSNSLAVVLQRRDWENPGVTQLNRLAAHPPFASWRNSEEARTDRPSQQLRSLNGEWRLMRYFLLTHLCGISHR
STICSDAA"
/label="lacZalpha CDS"

gene complement(146..415)
/gene="lacZalpha"
/label="lacZalpha gene"

misc_feature 183..188
/Recognition_pattern="CA^TATG"
/annotation_group="CATATG"
/label="NdeI"
/note="Geneious type: restriction site"

misc_feature 245..255
/Recognition_pattern="GCCNNNN^NGGC"
/annotation_group="GCCNNNNNGGC"
/label="BglI"
/note="Geneious type: restriction site"

misc_feature complement(391..415)
/created_by="User"
/label="pUC19productionR-Smi"
/note="Geneious type: primer_bind_reverse"

misc_feature 396..415
/gene="lacZalpha"
/note="multiple cloning site (EcoRI-HindIII)"
/label="lacZalpha misc"

misc_feature 396..401
/Recognition_pattern="G^AATTC"
/annotation_group="GAATTC"

```
[illegible]
/note="Geneious type: primer_bind_reverse"
misc_feature        396..415
/gene="lacZalpha"
/note="multiple cloning site (EcoRI-HindIII)"
/label="lacZalpha misc"
misc_feature        396..401
/Recognition_pattern="G^AATTC"
/annotation_group="GAATTC"
/label="EcoRI"
/note="Geneious type: restriction site"
primer_bind         401..440
/created_by="User"
/label="T3ko-5'F w/adp"
misc_feature        402..407
/Recognition_pattern="GAGCT^C"
/annotation_group="GAGCTC"
/label="SacI"
/note="Geneious type: restriction site"
misc_feature        412..415
/created_by="User"
/modified_by="User"
/label="SmiI5"
/note="Geneious type: restriction_site"
misc_feature        416..419
/Original_Bases
/label
/note="Geneious type: Editing History Insertion"
misc_feature        416..419
/created_by="User"
/label="Smi3"
/note="Geneious type: restriction_site"
misc_recomb         420..1465
/created_by="User"
/label="5'Recomb"
misc_feature        420..1465
/label="T3ko - 5'Recomb (concatenated sequence 2)"
/note="Geneious type: Concatenated sequence"
primer_bind         420..440
/created_by="User"
/label="T3ko-5'F"
misc_feature        complement(1445..1480)
/created_by="User"
```

```
     /label="T3ko-5'R w/adp"
     /note="Geneious type: primer_bind_reverse"
     misc_feature        complement(1445..1465)
     /created_by="User"
     /label="T3ko-5'R"
     /note="Geneious type: primer_bind_reverse"
     misc_feature        1466..3405
     /label="HXGPRT w/ KpnI (concatenated sequence 3)"
     /note="Geneious type: Concatenated sequence"
     primer_bind         1466..1492
     /created_by="User"
     /label="HXGPRT-KpnIF"
     misc_feature        1466..1471
     /created_by="User"
     /label="KpnI"
     /note="Geneious type: restriction_site"
     gene                1472..3401
     /created_by="User"
     /label="DHFR-HXGPRT"
     misc_feature        complement(1685..1707)
     /created_by="User"
     /label="5ex2R"
     /note="Geneious type: primer_bind_reverse"
     misc_feature        1934..1936
     /Original_Bases
     /label
     /note="Geneious type: Editing History Insertion"
     Selectable_Mark     1940..2632
     /label="HXGPRT"
     primer              1947..1966
     /note="Oligo predicted by CLC primer designer"
     /note="Oligo type: primer"
     /note="Melting temp.: 56.07"
     /label="HXGPRT detection"
     primer              complement(2662..2683)
     /note="Oligo predicted by CLC primer designer"
     /note="Oligo type: primer"
     /note="Melting temp.: 54.02"
     /label="HXGPRT detection"
     misc_feature        complement(3385..3405)
     /created_by="User"
     /label="HXGPRT-KpnIR"
```

/note="Geneious type: primer_bind_reverse"

primer_bind 3391..3426
/created_by="User"
/modified_by="User"
/label="T3ko-3'F w/adp"

misc_feature 3400..3405
/created_by="User"
/modified_by="User"
/label="KpnI"
/note="Geneious type: restriction_site"

misc_recomb 3406..4586
/created_by="User"
/label="3'Recomb"

misc_feature 3406..4586
/label="T3ko - 3'Recomb (concatenated sequence 4)"
/note="Geneious type: Concatenated sequence"

primer_bind 3406..3426
/created_by="User"
/modified_by="User"
/label="T3ko-3'F"

gene 3406..3859
/Source="annotation"
/Note="UDP-N-acetyl-D-galactosamine:polypeptide
N-acetylgalactosaminyltransferase T3, putative"
/soTerm="protein_coding"
/product="UDP-N-acetyl-D-galactosamine:polypeptide
N-acetylgalactosaminyltransferase T3, putative"
/taxon="Toxoplasma gondii ME49"
/isDeprecated=0
/isPseudo=0
/label="TGME49_118730"

UTR 3406..3859
/Source="annotation"
/isDeprecated=0
/label="UTR"

misc_feature complement(4566..4605)
/created_by="User"
/label="T3ko-3'R w/adp"
/note="Geneious type: primer_bind_reverse"

misc_feature complement(4566..4586)
/created_by="User"
/label="T3ko-3'R"

/note="Geneious type: primer_bind_reverse"

misc_feature 4587..4590
/Original_Bases
/label
/note="Geneious type: Editing History Insertion"

misc_feature 4587..6864
/label="pUC19 3' (concatenated sequence 5)"
/note="Geneious type: Concatenated sequence"

misc_feature 4587..4590
/created_by="User"
/label="Smi5"
/note="Geneious type: restriction_site"

misc_feature 4591..4630
/gene="lacZalpha"
/note="multiple cloning site (EcoRI-HindIII)"
/label="lacZalpha misc"

source 4591..6864
/organism="Cloning vector pUC19"
/mol_type="other DNA"
/label="source Cloning vector pUC19"

CDS complement(4591..4647)
/gene="lacZalpha"
/codon_start=1
/product="beta-galactosidase alpha fragment"
/translation="MTMITPSLHACRSTLEDPRVPSSNSLAVVLQRRDWENPGVTQLNRLAAHPPFASWRNSEEARTD
RPSQQLRSLNGEWRLMRYFLLTHLCGISHRIWCTLSTICSDAA"
/label="lacZalpha CDS"

gene complement(4591..4647)
/gene="lacZalpha"
/label="lacZalpha gene"

primer_bind 4591..4616
/created_by="User"
/label="pUC19productionF-Smi"

misc_feature 4591..4594
/created_by="User"
/modified_by="User"
/label="SmiI3"
/note="Geneious type: restriction_site"

misc_feature 4595..4600
/Recognition_pattern="G^GATCC"
/annotation_group="GGATCC"
/label="BamHI"

```
     /note="Geneious type: restriction site"
     misc_feature        4601..4606
     /Recognition_pattern="T^CTAGA"
     /annotation_group="TCTAGA"
     /label="XbaI"
     /note="Geneious type: restriction site"
     misc_feature        4607..4612
     /Recognition_pattern="G^TCGAC"
     /annotation_group="GTCGAC"
     /label="SalI"
     /note="Geneious type: restriction site"
     misc_feature        4613..4618
     /Recognition_pattern="CTGCA^G"
     /annotation_group="CTGCAG"
     /label="PstI"
     /note="Geneious type: restriction site"
     misc_feature        4625..4630
     /Recognition_pattern="A^AGCTT"
     /annotation_group="AAGCTT"
     /label="HindIII"
     /note="Geneious type: restriction site"
     -10_signal          complement(4692..4697)
     /note="Plac promoter (drives lacZalpha;
     counter-clockwise); TATGTT"
     /label="-10 signal"
     -35_signal          complement(4716..4721)
     /note="Plac promoter (drives lacZalpha;
     counter-clockwise); TTTACA"
     /label="-35 signal"
     protein_bind        complement(4741..4753)
     /note="CAP protein binding site"
     /bound_moiety="catabolite activator protein (CAP)"
     /label="CAP protein binding site protein bind"
     rep_origin          complement(5045..5633)
     /note="pMB1 origin of replication (counter-clockwise)
     (RNAII -35 to RNA/DNA switch point)"
     /label="rep origin"
     misc_RNA            complement(5045..5597)
     /note="RNAII transcript (complementary strand)"
     /label="RNA"
     -35_signal          5451..5456
     /note="RNAI promoter (clockwise); TTGAAG"
```

```
/label="RNAI promoter (clockwise); TTGAAG -35 signal"
-10_signal          5473..5478
/note="RNAI promoter (clockwise); GCTACA"
/label="RNAI promoter (clockwise); GCTACA -10 signal"
misc_RNA            5487..5594
/note="RNAI transcript"
/label="RNAI transcript"
-10_signal          complement(5607..5612)
/note="RNAII promoter (counter-clockwise); CGTAAT"
/label="-10 signal"
-35_signal          complement(5628..5633)
/note="RNAII promoter (counter-clockwise); TTGAGA"
/label="-35 signal"
CDS                 complement(5804..6664)
/gene="bla"
/note="ampR (confers resistance to ampicillin)"
/codon_start=1
/product="beta-lactamase"
/translation="MSIQHFRVALIPFFAAFCLPVFAHPETLVKVKDAEDQLGARVGYIELDLNSGKILESFRPEERF
PMMSTFKVLLCGAVLSRIDAGQEQLGRRIHYSQNDLVEYSPVTEKHLTDGMTVRELCSAAITMSDNTAANLLLTTIGG
PKELTAFLHNMGDHVTRLDRWEPELNEAIPNDERDTTMPVAMATTLRKLLTGELLTLASRQQLIDWMEADKVAGPLL
RSALPAGWFIADKSGAGERGSRGIIAALGPDGKPSRIVVIYTTGSQATMDERNRQIAEIGASLIKHW"
/label="bla CDS"
gene                complement(5804..6664)
/gene="bla"
/label="bla gene"
misc_feature        5991..6001
/Recognition_pattern="GCCNNNN^NGGC"
/annotation_group="GCCNNNNNGGC"
/label="BglI"
/note="Geneious type: restriction site"
sig_peptide         complement(6596..6664)
/gene="bla"
/note="required for secretion to the periplasm; cleaved
off to form the mature beta-lactamase protein."
/label="bla signal peptide"
-10_signal          complement(6708..6713)
/note="bla promoter (counter-clockwise); GAGACA"
/label="-10 signal"
-35_signal          complement(6729..6734)
/note="bla promoter (counter-clockwise); TTCAAA"
/label="-35 signal"
```

```
         10        20        30        40
tcgcgcgtttcggtgatgacggtgaaaacctctgacacat 40
gcagctcccggagacggtcacagcttgtctgtaagcggat 80
gccgggagcagacaagcccgtcagggcgcgtcagcgggtg 120
ttggcgggtgtcggggctggcttaactatgcggcatcaga 160
gcagattgtactgagagtgcaccatatgcggtgtgaaata 200
        210       220       230       240
ccgcacagatgcgtaaggagaaaataccgcatcaggcgcc 240
attcgccattcaggctgcgcaactgttgggaagggcgatc 280
ggtgcgggcctcttcgctattacgccagctggcgaaaggg 320
ggatgtgctgcaaggcgattaagttgggtaacgccagggt 360
tttcccagtcacgacgttgtaaaacgacggccagtgaatt 400
        410       420       430       440
cgagctcggtaatttaaataaagatgcgccgccgatcaaa 440
cagcgagaactttaagacctcaagcgcgccttagggcgcg 480
aacagcagagagaaaagctgcgtgctagaggaaacgaaac 520
ccagtcaacatttgtaaaaccgccatgttttccggggtta 560
tttattttatacgagaacccgcaggacacagctggagag 600
        610       620       630       640
ctgttggggaaaaaagcctttgtgtgtgcgagtgcatgtg 640
caagggaggtaaactggctccgccgctcccctgcgctggc 680
aatttgccattcttacagctctcgtttccttccccttcct 720
gtcgctatacgcttttcgtttcaggggtgctttgtttctg 760
cgagaccagtgccttgccccttctcgtatgtcaccccgtc 800
        810       820       830       840
tgtgttccgggatgctgctgcgtgcaccgagtctggctcg 840
cttcgaggaaaacgaacgagtgtgagtttgactcacttct 880
gctcgaaaagtcaaatgcgtgtttttctccactggtagga 920
caaatgtacttattttggccatcaacagacgcctgagccc 960
acactgctgcacatccgcccctgttagacaggtcggcatt 1000
       1010      1020      1030      1040
gctttgctcgtgtgtgcgcggcatctgcgtgagacacaac 1040
tatccagtgtgcacatgcttgttagtaactatgcgaaaga 1080
gccacaaaatccgctgatcatacatctatgcatgcttgat 1120
catgcgtgattcagtgtcagcaagtgcgagaaactgccgg 1160
tttgccggcaagagtcgctttttctagctgcatcaccaag 1200
       1210      1220      1230      1240
agaccaactagaggaagtacatcgtgttcgtcgtcccgac 1240
atctccatttcctgtgtgtctgcgtgctttgcttttctct 1280
caaccttccgttttcgtttctgttcgcgaaatgtcgatc 1320
gaacagtgcaattctctgttgtccctcactgcgtttgcca 1360
atgccataagacagttacgtcctgtcacacaaatcgccat 1400
```

```
        1410      1420      1430      1440
....|....|....|....|....|....|....|....|
cattcccctgtctcttccacgtttctgcacaagcacctc 1440
acgtggtcggcggtgctcatttgaaggtaccttgatcagc 1480
acgaaaccttgcattcaaacccgcccgcggaagatccgat 1520
cttgctgctgttcgcagtcccagtagcgtcctgtcggccg 1560
cgccgtctctgttggtgggcagccgctacacctgttatct 1600
        1610      1620      1630      1640
....|....|....|....|....|....|....|....|
gactgccgtgcgcgaaaatgacgccattttgggaaaatc 1640
ggggaacttcattctttaaaagtatgcggaggtttccttt 1680
ttcttctgttcgtttctttttctcgggtttgataaccgtg 1720
ttcgatgtaagcactttccgtctctcctccgtgctttgtt 1760
cgacatcgagaccaggtgtgcagatccttcgcttgtcgat 1800
        1810      1820      1830      1840
....|....|....|....|....|....|....|....|
ccggagacgcgtgtctcgtagaacctttcatttaccac 1840
acggcagtgcggagcactgctctgagtgcagcagggacgg 1880
gtgaagtttcgctttagtagtgcgtttctgctctacgggg 1920
cgttgtcagatccagcaaaatggcgtccaaacccattgaa 1960
gactacggcaagggcaagggccgtattgagcccatgtata 2000
        2010      2020      2030      2040
....|....|....|....|....|....|....|....|
tccccgacaacaccttctacaacgctgatgactttcttgt 2040
gcccccccactgcaagccctacattgacaaaatcctcctc 2080
cctggtggattggtcaaggacagagttgagaagttggcgt 2120
atgacatccacagaacttacttcggcgaggagttgcacat 2160
catttgcatcctgaaaggctctcgcggcttcttcaacctt 2200
        2210      2220      2230      2240
....|....|....|....|....|....|....|....|
ctgatcgactaccttgccaccatacagaagtacagtggtc 2240
gtgagtccagcgtgcccccttcttcgagcactatgtccg 2280
cctgaagtcctaccagaacgacaacagcacaggccagctc 2320
accgtcttgagcgacgacttgtcaatctttcgcgacaagc 2360
acgttctgattgttgaggacatcgtcgacaccggtttcac 2400
        2410      2420      2430      2440
....|....|....|....|....|....|....|....|
cctcaccgagttcggtgagcgcctgaaagccgtcggtccc 2440
aagtcgatgagaatcgccaccctcgtcgagaagcgcacag 2480
atcgctccaacagcttgaagggcgacttcgtcggcttcag 2520
cattgaagacgtctggatcgttggttgctgctacgacttc 2560
aacgagatgttccgcgacttcgaccacgtcgccgtcctga 2600
        2610      2620      2630      2640
....|....|....|....|....|....|....|....|
gcgacgccgctcgcaaaaagttcgagaagtaaaccctgca 2640
tagccctacagaagctgcccgtctctcgttttcctctctt 2680
ttcggagggatcagggagagtgcctcgggtcggagagagc 2720
tgacgaggggggtgccagagacccctgtgtcctttatcgaa 2760
gaaaagggatgactcttcatgtggcatttcacacagtctc 2800
```

```
        2810      2820      2830      2840

acctcgccttgtttcttttgtcaatcagaacgaaagcg 2840
agttgcgggtgacgcagatgtgcgtgtatccactcgtgaa 2880
tgcgttatcgttctgtatgccgctagagtgctggactgtt 2920
gctgtctgcccacgacagcagacaactttccttctatgca 2960
cttgcaggatggtgcagcgcaaacgacggagagaaaggag 3000

        3010      3020      3030      3040

caccctctcagtttccctacgatgtgctgtcagtttcgac 3040
tcttcaccgcgaacgattggcgatacgtctctgttgactt 3080
gttaggctccgaccacgaagctcccttaactaaataagcc 3120
gcgacacctaagtgtacaccatttgcagatcgataatctg 3160
cgaccgctgaatccgtccagatcaggaaaaccgcaccacc 3200

        3210      3220      3230      3240

taagtgtaaaccttgtttaggtcgataaaatgctaccaac 3240
ccccacccacaatcgagccttgagcgtttctgcgcacgcg 3280
ttggcctacgtgacttgctgatgcctgcctctggccattc 3320
atgccagtcagtgcgcataaaaatgtggacacagtcggtt 3360
gacaagtgttctggcaggctacagtgacaccgcggtggag 3400

        3410      3420      3430      3440

gtacctgctgcggctgctgtaaaactggttgttgcagctg 3440
ttgcagctggatccgaggatgttggagatcataaatgctc 3480
cgtttaagtaatgggtggttggcccaagtgccacagagaa 3520
aaatgcgatttgtagaaccgtaacgaagcgactggttcac 3560
ctaattcctgcgtggcaagtgactcccgtgcgggtgtctc 3600

        3610      3620      3630      3640

aacgggagaatggtctcagatttcaatgttgtgctttata 3640
tcatctcgattcactgagcgtcagctcttcaatacattaa 3680
ggcggccgacagttgtgccccagacccatgtataggcact 3720
cgcgcaaagggtcccgtggtcattccttcaggaaagcttg 3760
ttgtgctccccataacttgtcttttcaagctattcgattc 3800

        3810      3820      3830      3840

tctagattccgtgtacgaacccctgaaccgggtcaactca 3840
tccgcttgattcgcatcgtctacacagaacccaatgaact 3880
acaccaatgcacgcagtctagaaaactgtccaataccttc 3920
agaaggtggtgaactcgtgctcgagatacgtaacttcccc 3960
atgcattagtctggttactgggaagagattcgttagttcg 4000

        4010      4020      4030      4040

gcgctacgttgtagtcagaattcttggttgtctgatgaaa 4040
cacaagagcgctcttgacccgcgaagtactgcgggtgagc 4080
atgtagacgcccagcacgagtgtttcattcgaacagggaa 4120
ttcccacgtagctccgcatgacctgacaacttcgggtgtg 4160
tgtgcattctgtatactgattgagctcacttggtaaaacc 4200
```

```
        4210      4220      4230      4240
ttgattctggaatgtccgtggtacacagcaatgtcctcgt 4240
ggacgcgagcgagttattcgccagttcctttcttgtaatt 4280
gtggaccatctgcaggtctcgaaggaagattatcactttt 4320
taaacggcatgtggcgttttcgttaggtcacatttaggtt 4360
tccactcccccggtcagttgccaacagcaacggaaagcac 4400
        4410      4420      4430      4440
aaaggaggaagctggtgagggacttcacggcttttacac 4440
agcgcactatggtgcccagtcatggtatacgcttgcatcc 4480
agtgttcgagcacacaaaggcgaatgtacaccctttgaca 4520
accgaagcgactccggcgatgcctactctctaatggattg 4560
aaaattcgtgcatgcgtccactctttatttaaatggatcc 4600
        4610      4620      4630      4640
tctagagtcgacctgcaggcatgcaagcttggcgtaatca 4640
tggtcatagctgtttcctgtgtgaaattgttatccgctca 4680
caattccacacaacatacgagccggaagcataaagtgtaa 4720
agcctggggtgcctaatgagtgagctaactcacattaatt 4760
gcgttgcgctcactgcccgctttccagtcgggaaacctgt 4800
        4810      4820      4830      4840
cgtgccagctgcattaatgaatcggccaacgcgcggggag 4840
aggcggtttgcgtattgggcgctcttccgcttcctcgctc 4880
actgactcgctgcgctcggtcgttcggctgcggcgagcgg 4920
tatcagctcactcaaaggcggtaatacggttatccacaga 4960
atcaggggataacgcaggaaagaacatgtgagcaaaaggc 5000
        5010      5020      5030      5040
cagcaaaaggccaggaaccgtaaaaaggccgcgttgctgg 5040
cgtttttccataggctccgcccccctgacgagcatcacaa 5080
aaatcgacgctcaagtcagaggtggcgaaacccgacagga 5120
ctataaagataccaggcgtttccccctggaagctccctcg 5160
tgcgctctcctgttccgaccctgccgcttaccggatacct 5200
        5210      5220      5230      5240
gtccgcctttctcccttcgggaagcgtggcgctttctcat 5240
agctcacgctgtaggtatctcagttcggtgtaggtcgttc 5280
gctccaagctgggctgtgtgcacgaaccccccgttcagcc 5320
cgaccgctgcgccttatccggtaactatcgtcttgagtcc 5360
aacccggtaagacacgacttatcgccactggcagcagcca 5400
        5410      5420      5430      5440
ctggtaacaggattagcagagcgaggtatgtaggcggtgc 5440
tacagagttcttgaagtggtggcctaactacggctacact 5480
agaagaacagtatttggtatctgcgctctgctgaagccag 5520
ttaccttcggaaaaagagttggtagctcttgatccggcaa 5560
acaaaccaccgctggtagcggtggtttttttgtttgcaag 5600
```

```
        5610      5620      5630      5640
cagcagattacgcgcagaaaaaaaggatctcaagaagatc 5640
ctttgatcttttctacggggtctgacgctcagtggaacga 5680
aaactcacgttaagggattttggtcatgagattatcaaaa 5720
aggatcttcacctagatccttttaaattaaaaatgaagtt 5760
ttaaatcaatctaaagtatatatgagtaaacttggtctga 5800
        5810      5820      5830      5840
cagttaccaatgcttaatcagtgaggcacctatctcagcg 5840
atctgtctatttcgttcatccatagttgcctgactccccg 5880
tcgtgtagataactacgatacgggagggcttaccatctgg 5920
ccccagtgctgcaatgataccgcgagacccacgctcaccg 5960
gctccagatttatcagcaataaaccagccagccggaaggg 6000
        6010      6020      6030      6040
ccgagcgcagaagtggtcctgcaactttatccgcctccat 6040
ccagtctattaattgttgccgggaagctagagtaagtagt 6080
tcgccagttaatagtttgcgcaacgttgttgccattgcta 6120
caggcatcgtggtgtcacgctcgtcgtttggtatggcttc 6160
attcagctccggttcccaacgatcaaggcgagttacatga 6200
        6210      6220      6230      6240
tcccccatgttgtgcaaaaaagcggttagctccttcggtc 6240
ctccgatcgttgtcagaagtaagttggccgcagtgttatc 6280
actcatggttatggcagcactgcataattctcttactgtc 6320
atgccatccgtaagatgcttttctgtgactggtgagtact 6360
caaccaagtcattctgagaatagtgtatgcggcgaccgag 6400
        6410      6420      6430      6440
ttgctcttgcccggcgtcaatacgggataataccgcgcca 6440
catagcagaactttaaaagtgctcatcattggaaaacgtt 6480
cttcggggcgaaaactctcaaggatcttaccgctgttgag 6520
atccagttcgatgtaacccactcgtgcacccaactgatct 6560
tcagcatcttttactttcaccagcgtttctgggtgagcaa 6600
        6610      6620      6630      6640
aaacaggaaggcaaaatgccgcaaaaaagggaataagggc 6640
gacacggaaatgttgaatactcatactcttcctttttcaa 6680
tattattgaagcatttatcagggttattgtctcatgagcg 6720
gatacatatttgaatgtatttagaaaaataaacaaatagg 6760
ggttccgcgcacatttccccgaaaagtgccacctgacgtc 6800
        6810      6820      6830      6840
taagaaaccattattatcatgacattaacctataaaaata 6840
ggcgtatcacgaggccctttcgtc 6864
```

```
LOCUS       pUC19_T4KO_vecto        7070 bp    DNA
 circular UNA 17-DEC-2012
DEFINITION  Concatenation of 5 sequences.
ACCESSION   urn.local...1317877761435.35
VERSION     urn.local...1317877761435.35
KEYWORDS    .
SOURCE
  ORGANISM
            .

ORIGIN
Features
     source          1..415
     /organism="Cloning vector pUC19"
     /mol_type="other DNA"
     /label="source Cloning vector pUC19"
     misc_feature    1..419
     /label="pUC19 5' (concatenated sequence 1)"
     /note="Geneious type: Concatenated sequence"
     CDS             complement(146..415)
     /gene="lacZalpha"
     /codon_start=1
     /product="beta-galactosidase alpha fragment"
     /translation="MTMITPSLHACRSTLEDPRVPSSNSLAVVLQRRDWENPGVTQLNRLAAHPPFASWRNSEEARTDRPSQQLRSLNGEWRLMRYFLLTHLCGISHR
     STICSDAA"
     /label="lacZalpha CDS"
     gene            complement(146..415)
     /gene="lacZalpha"
     /label="lacZalpha gene"
     misc_feature    183..188
     /Recognition_pattern="CA^TATG"
     /annotation_group="CATATG"
     /label="NdeI"
     /note="Geneious type: restriction site"
     misc_feature    245..255
     /Recognition_pattern="GCCNNNN^NGGC"
     /annotation_group="GCCNNNNNGGC"
     /label="BglI"
     /note="Geneious type: restriction site"
     misc_feature    complement(391..415)
     /created_by="User"
     /label="pUC19productionR-Smi"
     /note="Geneious type: primer_bind_reverse"
     misc_feature    396..415
     /gene="lacZalpha"
     /note="multiple cloning site (EcoRI-HindIII)"
     /label="lacZalpha misc"
     misc_feature    396..401
     /Recognition_pattern="G^AATTC"
     /annotation_group="GAATTC"
```

```
/label=[illegible]
/note="Geneious type: primer_bind_reverse"
misc_feature        396..415
/gene="lacZalpha"
/note="multiple cloning site (EcoRI-HindIII)"
/label="lacZalpha misc"
misc_feature        396..401
/Recognition_pattern="G^AATTC"
/annotation_group="GAATTC"
/label="EcoRI"
/note="Geneious type: restriction site"
primer_bind         401..440
/created_by="User"
/label="T4ko-5'F w/adp"
misc_feature        402..407
/Recognition_pattern="GAGCT^C"
/annotation_group="GAGCTC"
/label="SacI"
/note="Geneious type: restriction site"
misc_feature        412..415
/created_by="User"
/modified_by="User"
/label="SmiI5"
/note="Geneious type: restriction_site"
misc_feature        416..419
/Original_Bases
/label
/note="Geneious type: Editing History Insertion"
misc_feature        416..419
/created_by="User"
/label="Smi3"
/note="Geneious type: restriction_site"
misc_recomb         420..1687
/created_by="User"
/label="5'Recomb"
misc_feature        420..1687
/label="T4ko - 5'Recomb (concatenated sequence 2)"
/note="Geneious type: Concatenated sequence"
primer_bind         420..440
/created_by="User"
/label="T4ko-5'F"
misc_feature        complement(1667..1702)
/created_by="User"
```

```
     /label="T4ko-5'R w/adp"
     /note="Geneious type: primer_bind_reverse"
     misc_feature        complement(1667..1687)
     /created_by="User"
     /label="T4ko-5'R"
     /note="Geneious type: primer_bind_reverse"
     misc_feature        1688..3627
     /label="HXGPRT w/ KpnI (concatenated sequence 3)"
     /note="Geneious type: Concatenated sequence"
     primer_bind         1688..1714
     /created_by="User"
     /label="HXGPRT-KpnIF"
     misc_feature        1688..1693
     /created_by="User"
     /label="KpnI"
     /note="Geneious type: restriction_site"
     gene                1694..3623
     /created_by="User"
     /label="DHFR-HXGPRT"
     misc_feature        complement(1907..1929)
     /created_by="User"
     /label="5ex2R"
     /note="Geneious type: primer_bind_reverse"
     misc_feature        2156..2158
     /Original_Bases
     /label
     /note="Geneious type: Editing History Insertion"
     Selectable_Mark     2162..2854
     /label="HXGPRT"
     primer              2169..2188
     /note="Oligo predicted by CLC primer designer"
     /note="Oligo type: primer"
     /note="Melting temp.: 56.07"
     /label="HXGPRT detection"
     primer              complement(2884..2905)
     /note="Oligo predicted by CLC primer designer"
     /note="Oligo type: primer"
     /note="Melting temp.: 54.02"
     /label="HXGPRT detection"
     misc_feature        complement(3607..3627)
     /created_by="User"
     /label="HXGPRT-KpnIR"
```

```
      /note="Geneious type: primer_bind_reverse"
      primer_bind         3613..3648
      /created_by="User"
      /label="T4ko-3'F w/adp"
      misc_feature        3622..3627
      /created_by="User"
      /modified_by="User"
      /label="KpnI"
      /note="Geneious type: restriction_site"
      misc_recomb         3628..4792
      /created_by="User"
      /label="3'Recomb"
      misc_feature        3628..4792
      /label="T4ko - 3'Recomb (concatenated sequence 4)"
      /note="Geneious type: Concatenated sequence"
      primer_bind         3628..3648
      /created_by="User"
      /label="T4ko-3'F"
      misc_feature        complement(4772..4811)
      /created_by="User"
      /label="T4ko-3'R w/adp"
      /note="Geneious type: primer_bind_reverse"
      misc_feature        complement(4772..4792)
      /created_by="User"
      /modified_by="User"
      /label="T4ko-3'R"
      /note="Geneious type: primer_bind_reverse"
      misc_feature        4793..4796
      /Original_Bases
      /label
      /note="Geneious type: Editing History Insertion"
      misc_feature        4793..7070
      /label="pUC19 3' (concatenated sequence 5)"
      /note="Geneious type: Concatenated sequence"
      misc_feature        4793..4796
      /created_by="User"
      /label="Smi5"
      /note="Geneious type: restriction_site"
      misc_feature        4797..4836
      /gene="lacZalpha"
      /note="multiple cloning site (EcoRI-HindIII)"
      /label="lacZalpha misc"
```

```
source                4797..7070
/organism="Cloning vector pUC19"
/mol_type="other DNA"
/label="source Cloning vector pUC19"
```

```
CDS                   complement(4797..4853)
/gene="lacZalpha"
/codon_start=1
/product="beta-galactosidase alpha fragment"
/translation="MTMITPSLHACRSTLEDPRVPSSNSLAVVLQRRDWENPGVTQLNRLAAHPPFASWRNSEEARTD
RPSQQLRSLNGEWRLMRYFLLTHLCGISHRIWCTLSTICSDAA"
/label="lacZalpha CDS"
```

```
gene                  complement(4797..4853)
/gene="lacZalpha"
/label="lacZalpha gene"
```

```
primer_bind           4797..4822
/created_by="User"
/label="pUC19productionF-Smi"
```

```
misc_feature          4797..4800
/created_by="User"
/modified_by="User"
/label="SmiI3"
/note="Geneious type: restriction_site"
```

```
misc_feature          4801..4806
/Recognition_pattern="G^GATCC"
/annotation_group="GGATCC"
/label="BamHI"
/note="Geneious type: restriction site"
```

```
misc_feature          4807..4812
/Recognition_pattern="T^CTAGA"
/annotation_group="TCTAGA"
/label="XbaI"
/note="Geneious type: restriction site"
```

```
misc_feature          4813..4818
/Recognition_pattern="G^TCGAC"
/annotation_group="GTCGAC"
/label="SalI"
/note="Geneious type: restriction site"
```

```
misc_feature          4819..4824
/Recognition_pattern="CTGCA^G"
/annotation_group="CTGCAG"
/label="PstI"
/note="Geneious type: restriction site"
```

```
misc_feature        4831..4836
/Recognition_pattern="A^AGCTT"
/annotation_group="AAGCTT"
/label="HindIII"
/note="Geneious type: restriction site"
```

```
-10_signal          complement(4898..4903)
/note="Plac promoter (drives lacZalpha;
counter-clockwise); TATGTT"
/label="-10 signal"
```

```
-35_signal          complement(4922..4927)
/note="Plac promoter (drives lacZalpha;
counter-clockwise); TTTACA"
/label="-35 signal"
```

```
protein_bind        complement(4947..4959)
/note="CAP protein binding site"
/bound_moiety="catabolite activator protein (CAP)"
/label="CAP protein binding site protein bind"
```

```
rep_origin          complement(5251..5839)
/note="pMB1 origin of replication (counter-clockwise)
(RNAII -35 to RNA/DNA switch point)"
/label="rep origin"
```

```
misc_RNA            complement(5251..5803)
/note="RNAII transcript (complementary strand)"
/label="RNA"
```

```
-35_signal          5657..5662
/note="RNAI promoter (clockwise); TTGAAG"
/label="RNAI promoter (clockwise); TTGAAG -35 signal"
```

```
-10_signal          5679..5684
/note="RNAI promoter (clockwise); GCTACA"
/label="RNAI promoter (clockwise); GCTACA -10 signal"
```

```
misc_RNA            5693..5800
/note="RNAI transcript"
/label="RNAI transcript"
```

```
-10_signal          complement(5813..5818)
/note="RNAII promoter (counter-clockwise); CGTAAT"
/label="-10 signal"
```

```
-35_signal          complement(5834..5839)
/note="RNAII promoter (counter-clockwise); TTGAGA"
/label="-35 signal"
```

```
CDS                 complement(6010..6870)
/gene="bla"
/note="ampR (confers resistance to ampicillin)"
```

/codon_start=1
/product="beta-lactamase"
/translation="MSIQHFRVALIPFFAAFCLPVFAHPETLVKVKDAEDQLGARVGYIELDLNSGKILESFRPEERF
PMMSTFKVLLCGAVLSRIDAGQEQLGRRIHYSQNDLVEYSPVTEKHLTDGMTVRELCSAAITMSDNTAANLLLTTIGG
PKELTAFLHNMGDHVTRLDRWEPELNEAIPNDERDTTMPVAMATTLRKLLTGELLTLASRQQLIDWMEADKVAGPLL
RSALPAGWFIADKSGAGERGSRGIIAALGPDGKPSRIVVIYTTGSQATMDERNRQIAEIGASLIKHW"
/label="bla CDS"

---

gene complement(6010..6870)
/gene="bla"
/label="bla gene"

---

misc_feature 6197..6207
/Recognition_pattern="GCCNNNN^NGGC"
/annotation_group="GCCNNNNNGGC"
/label="BglI"
/note="Geneious type: restriction site"

---

sig_peptide complement(6802..6870)
/gene="bla"
/note="required for secretion to the periplasm; cleaved
off to form the mature beta-lactamase protein."
/label="bla signal peptide"

-10_signal complement(6914..6919)
/note="bla promoter (counter-clockwise); GAGACA"
/label="-10 signal"

-35_signal complement(6935..6940)
/note="bla promoter (counter-clockwise); TTCAAA"
/label="-35 signal"

```
        10        20        30        40
tcgcgcgtttcggtgatgacggtgaaaacctctgacacat 40
gcagctcccggagacggtcacagcttgtctgtaagcggat 80
gccgggagcagacaagcccgtcagggcgcgtcagcgggtg 120
ttggcgggtgtcggggctggcttaactatgcggcatcaga 160
gcagattgtactgagagtgcaccatatgcggtgtgaaata 200
       210       220       230       240
ccgcacagatgcgtaaggagaaaataccgcatcaggcgcc 240
attcgccattcaggctgcgcaactgttgggaagggcgatc 280
ggtgcgggcctcttcgctattacgccagctggcgaaaggg 320
ggatgtgctgcaaggcgattaagttgggtaacgccagggt 360
tttcccagtcacgacgttgtaaaacgacggccagtgaatt 400
```

```
        410       420       430       440
----|----|----|----|----|----|----|----|
cgagctcggtaatttaaattttcaggcgccacaccatcg 440
tgaaggtgtggcgctgaagagcagggaagaaggccgccat 480
cctcagtagacgggaacggagagacgcgacgaggcgcaga 520
aagtaaagttgcagcactttcccattgatttgaaaccgct 560
ccggaagttgataagcaaggtaaagtagagtttccgcctt 600
        610       620       630       640
----|----|----|----|----|----|----|----|
ctccgaagccatttccttgcatttgactcacgatatgtg 640
aggcaagacgagacagactggtcgaacgcgattgtcgcgc 680
ttcaagaggcgactacgcgttcacgggcttcacacagcca 720
tggaggagagggaagtgcctggtgaagcagaaacagaaga 760
gatctcacaggttcaaaagatgtgagaaatatttgacgaa 800
        810       820       830       840
----|----|----|----|----|----|----|----|
actaaaactagctgggagaggtggcaactctggactggag 840
ggaggaggggaggaaacgacttggaggccggggtggtccg 880
acaagacagtgtgtgccaagtaacggcagtttctgaaaag 920
aacaacagtttgctccgccactgccacgagagtccaccct 960
acgagatttgccgtggcaacacaacaccagcacgggctat 1000
       1010      1020      1030      1040
----|----|----|----|----|----|----|----|
caccaccaactgttctgtgtggagcttcagcttccaccga 1040
caaagtgaggaaacagatccggtagccgcattgtcacccg 1080
tgattcgcatgcaaagacaagatgccactaacaggggttc 1120
aacaaggcggttgctggttcggctgtcctctgttacgcat 1160
tttttggagaacgaagtggctcgaaaacaaagtttcacag 1200
       1210      1220      1230      1240
----|----|----|----|----|----|----|----|
agcatttcaactctgatatgaaagcaaattattcgaattt 1240
gccgggggatattggacgcgttctaacccacgactgcact 1280
atctagtagcccggatcgcgcgcccaacgctgtcaccact 1320
tctactttacggtaaccattagcatgtgtttttcttccg 1360
gtccctgaacgagaaagcaactcgaaggaaagttactgag 1400
       1410      1420      1430      1440
----|----|----|----|----|----|----|----|
cctaagaaccttctgggatacgaacttagcagcaggagag 1440
acaagtttttgctttgcctcggaagttcctttacaaaaag 1480
gacgaacatcttttggctcagatgaggcactgccttcagc 1520
ggaaccgcgataaaaatagttgttgctacacgttgagcgc 1560
actttccttccaaacatttcaactcaggaatccagtcgtg 1600
       1610      1620      1630      1640
----|----|----|----|----|----|----|----|
tttcctggaggcacactagacctgtagtagcttttgtct 1640
cgcgcttaatgggaaattcttccccagtggcgaattcctg 1680
tgccagtggtaccttgatcagcacgaaaccttgcattcaa 1720
acccgcccgcggaagatccgatcttgctgctgttcgcagt 1760
cccagtagcgtcctgtcggccgcgccgtctctgttggtgg 1800
```

```
        1810      1820      1830      1840
gcagccgctacacctgttatctgactgccgtgcgcgaaaa 1840
tgacgccatttttgggaaaatcggggaacttcattcttta 1880
aaagtatgcggaggtttcctttttcttctgttcgtttctt 1920
tttctcgggtttgataaccgtgttcgatgtaagcactttc 1960
cgtctctcctccgtgctttgttcgacatcgagaccaggtg 2000
        2010      2020      2030      2040
tgcagatccttcgcttgtcgatccggagacgcgtgtctcg 2040
tagaaccttttcattttaccacacggcagtgcggagcact 2080
gctctgagtgcagcagggacgggtgaagtttcgctttagt 2120
agtgcgtttctgctctacggggcgttgtcagatccagcaa 2160
aatggcgtccaaacccattgaagactacggcaagggcaag 2200
        2210      2220      2230      2240
ggccgtattgagcccatgtatatccccgacaacaccttct 2240
acaacgctgatgactttcttgtgcccccccactgcaagcc 2280
ctacattgacaaaatcctcctccctggtggattggtcaag 2320
gacagagttgagaagttggcgtatgacatccacagaactt 2360
acttcggcgaggagttgcacatcatttgcatcctgaaagg 2400
        2410      2420      2430      2440
ctctcgcggcttcttcaaccttctgatcgactaccttgcc 2440
accatacagaagtacagtggtcgtgagtccagcgtgcccc 2480
ccttcttcgagcactatgtccgcctgaagtcctaccagaa 2520
cgacaacagcacaggccagctcaccgtcttgagcgacgac 2560
ttgtcaatctttcgcgacaagcacgttctgattgttgagg 2600
        2610      2620      2630      2640
acatcgtcgacaccggtttcaccctcaccgagttcggtga 2640
gcgcctgaaagccgtcggtcccaagtcgatgagaatcgcc 2680
accctcgtcgagaagcgcacagatcgctccaacagcttga 2720
agggcgacttcgtcggcttcagcattgaagacgtctggat 2760
cgttggttgctgctacgacttcaacgagatgttccgcgac 2800
        2810      2820      2830      2840
ttcgaccacgtcgccgtcctgagcgacgccgctcgcaaaa 2840
agttcgagaagtaaaccctgcatagccctacagaagctgc 2880
ccgtctctcgttttcctctcttttcggagggatcagggag 2920
agtgcctcgggtcggagagagctgacgagggggtgccaga 2960
gacccctgtgtcctttatcgaagaaaagggatgactcttc 3000
        3010      3020      3030      3040
atgtggcatttcacacagtctcacctcgccttgtttttctt 3040
tttgtcaatcagaacgaaagcgagttgcgggtgacgcaga 3080
tgtgcgtgtatccactcgtgaatgcgttatcgttctgtat 3120
gccgctagagtgctggactgttgctgtctgcccacgacag 3160
cagacaactttccttctatgcacttgcaggatggtgcagc 3200
```

```
        3210      3220      3230      3240
gcaaacgacggagagaaaggagcaccctctcagtttccct 3240
acgatgtgctgtcagtttcgactcttcaccgcgaacgatt 3280
ggcgatacgtctctgttgacttgttaggctccgaccacga 3320
agctcccttaactaaataagccgcgacacctaagtgtaca 3360
ccatttgcagatcgataatctgcgaccgctgaatccgtcc 3400
        3410      3420      3430      3440
agatcaggaaaaccgcaccacctaagtgtaaaccttgttt 3440
aggtcgataaaatgctaccaacccccacccacaatcgagc 3480
cttgagcgtttctgcgcacgcgttggcctacgtgacttgc 3520
tgatgcctgcctctggccattcatgccagtcagtgcgcat 3560
aaaaatgtggacacagtcggttgacaagtgttctggcagg 3600
        3610      3620      3630      3640
ctacagtgacaccgcggtggaggtaccaacggggagcgtt 3640
ccttctctgaagggtttctagtcacgccgactgcttgcta 3680
acaccgatcctcattatggcctcctgtgacaatcaggcta 3720
ctgccatccaccttacctttcgcggctggtcttcagcgaa 3760
actaacacaaggacgagagaagacgcgggctcgtcccaca 3800
        3810      3820      3830      3840
ccctcacctgcggctatgaacctgacacggctgcgtcagg 3840
aaaactgcacacgacaccccatgaacactttgtgtactgt 3880
cactatgtcctttattctacataaggagggtcacaggaga 3920
gcctcacgtgtctctcggcgacatcagcgaccgtataaaa 3960
ctgcgagagcggcttgggtgcaaatcatttcaatggtttc 4000
        4010      4020      4030      4040
tggacaacgtttggcccgaatccgatgtccgaagcctccg 4040
tgaggatgtgcctttcatgggtacgacttggaaaaacatg 4080
ggacactggataagtagatgtttccctaggaaggccaagc 4120
atagctacaaggctgcacagacgactaaggtaatgcgaaa 4160
caagcggcgtgtgatttcttttggtttgagatccgtgagc 4200
        4210      4220      4230      4240
atcgtcactgctaccgtgcgcccactttgcttcgtttcaa 4240
caattcgtgcgcagcctccacatattctaggtctatttg 4280
gattctaaaaaacactggcggtgtgacgttgggttcaccg 4320
ttgcagagtacggggttcgggtgtggtttcgtggtactca 4360
gattcctcggcatcggaatccactcctgtgtgagaaacac 4400
        4410      4420      4430      4440
tcaccaggcagccatggagtcatcacgcaacagctcccta 4440
acagtcgtcactggacatgagcaacttcatccctgacgag 4480
aacataattttttccctttagtgtttctttgcaggttcccc 4520
caccaccaggaaatgcttttcgtacgcacacacgaaagac 4560
agaaggcgcagatgatgctctactttcctgtgttgtctag 4600
```

```
        4610      4620      4630      4640
----|----|----|----|----|----|----|----|
ccgtctacttcttcaggcccgctcctcctgcgagagaacg 4640
catttctgtgcctcgacggtggccatcctcatccgggagg 4680
acaaacagcagatattacgaatgcatgcggtcgtgcagga 4720
accctattcatgtattttgttcgaacaatgcaagtcaaga 4760
tggcccgaaacgacgaagcttgcttgaacggcatttaaat 4800
        4810      4820      4830      4840
----|----|----|----|----|----|----|----|
ggatcctctagagtcgacctgcaggcatgcaagcttggcg 4840
taatcatggtcatagctgtttcctgtgtgaaattgttatc 4880
cgctcacaattccacacaacatacgagccggaagcataaa 4920
gtgtaaagcctggggtgcctaatgagtgagctaactcaca 4960
ttaattgcgttgcgctcactgcccgctttccagtcgggaa 5000
        5010      5020      5030      5040
----|----|----|----|----|----|----|----|
acctgtcgtgccagctgcattaatgaatcggccaacgcgc 5040
ggggagaggcggtttgcgtattgggcgctcttccgcttcc 5080
tcgctcactgactcgctgcgctcggtcgttcggctgcggc 5120
gagcggtatcagctcactcaaaggcggtaatacggttatc 5160
cacagaatcaggggataacgcaggaaagaacatgtgagca 5200
        5210      5220      5230      5240
----|----|----|----|----|----|----|----|
aaaggccagcaaaaggccaggaaccgtaaaaaggccgcgt 5240
tgctggcgtttttccataggctccgcccccctgacgagca 5280
tcacaaaaatcgacgctcaagtcagaggtggcgaaacccg 5320
acaggactataaagataccaggcgtttccccctggaagct 5360
ccctcgtgcgctctcctgttccgaccctgccgcttaccgg 5400
        5410      5420      5430      5440
----|----|----|----|----|----|----|----|
atacctgtccgcctttctcccttcgggaagcgtggcgctt 5440
tctcatagctcacgctgtaggtatctcagttcggtgtagg 5480
tcgttcgctccaagctgggctgtgtgcacgaaccccccgt 5520
tcagcccgaccgctgcgccttatccggtaactatcgtctt 5560
gagtccaacccggtaagacacgacttatcgccactggcag 5600
        5610      5620      5630      5640
----|----|----|----|----|----|----|----|
cagccactggtaacaggattagcagagcgaggtatgtagg 5640
cggtgctacagagttcttgaagtggtggcctaactacggc 5680
tacactagaagaacagtatttggtatctgcgctctgctga 5720
agccagttaccttcggaaaaagagttggtagctcttgatc 5760
cggcaaacaaaccaccgctggtagcggtggtttttttgtt 5800
        5810      5820      5830      5840
----|----|----|----|----|----|----|----|
tgcaagcagcagattacgcgcagaaaaaaaggatctcaag 5840
aagatcctttgatcttttctacggggtctgacgctcagtg 5880
gaacgaaaactcacgttaagggattttggtcatgagatta 5920
tcaaaaaggatcttcacctagatccttttaaattaaaaat 5960
gaagttttaaatcaatctaaagtatatatgagtaaacttg 6000
```

```
        6010      6020      6030      6040
gtctgacagttaccaatgcttaatcagtgaggcacctatc 6040
tcagcgatctgtctatttcgttcatccatagttgcctgac 6080
tccccgtcgtgtagataactacgatacgggagggcttacc 6120
atctggccccagtgctgcaatgataccgcgagacccacgc 6160
tcaccggctccagatttatcagcaataaaccagccagccg 6200
        6210      6220      6230      6240
gaagggccgagcgcagaagtggtcctgcaactttatccgc 6240
ctccatccagtctattaattgttgccgggaagctagagta 6280
agtagttcgccagttaatagtttgcgcaacgttgttgcca 6320
ttgctacaggcatcgtggtgtcacgctcgtcgtttggtat 6360
ggcttcattcagctccggttcccaacgatcaaggcgagtt 6400
        6410      6420      6430      6440
acatgatcccccatgttgtgcaaaaaagcggttagctcct 6440
tcggtcctccgatcgttgtcagaagtaagttggccgcagt 6480
gttatcactcatggttatggcagcactgcataattctctt 6520
actgtcatgccatccgtaagatgcttttctgtgactggtg 6560
agtactcaaccaagtcattctgagaatagtgtatgcggcg 6600
        6610      6620      6630      6640
accgagttgctcttgcccggcgtcaatacgggataatacc 6640
gcgccacatagcagaactttaaaagtgctcatcattggaa 6680
aacgttcttcggggcgaaaactctcaaggatcttaccgct 6720
gttgagatccagttcgatgtaacccactcgtgcacccaac 6760
tgatcttcagcatcttttactttcaccagcgtttctgggt 6800
        6810      6820      6830      6840
gagcaaaaacaggaaggcaaaatgccgcaaaaaagggaat 6840
aagggcgacacggaaatgttgaatactcatactcttcctt 6880
tttcaatattattgaagcatttatcagggttattgtctca 6920
tgagcggatacatatttgaatgtatttagaaaaataaaca 6960
aataggggttccgcgcacatttccccgaaaagtgccacct 7000
        7010      7020      7030      7040
gacgtctaagaaaccattattatcatgacattaacctata 7040
aaaataggcgtatcacgaggccctttcgtc 7070
```

```
LOCUS       pUC19_T5KO_vecto        7084 bp    DNA
 circular UNA 17-DEC-2012
DEFINITION  Concatenation of 5 sequences.
ACCESSION   urn.local...1317878330916.46
VERSION     urn.local...1317878330916.46
KEYWORDS    .
SOURCE
  ORGANISM
            .

ORIGIN
Features
     source             1..415
     /organism="Cloning vector pUC19"
     /mol_type="other DNA"
     /label="source Cloning vector pUC19"
     misc_feature       1..419
     /label="pUC19 5' (concatenated sequence 1)"
     /note="Geneious type: Concatenated sequence"
     CDS                complement(146..415)
     /gene="lacZalpha"
     /codon_start=1
     /product="beta-galactosidase alpha fragment"
     /translation="MTMITPSLHACRSTLEDPRVPSSNSLAVVLQRRDWENPGVTQLNRLAAHPPFASWRNSEEARTDRPSQQLRSLNGEWRLMRYFLLTHLCGISH[illegible]
     STICSDAA"
     /label="lacZalpha CDS"
     gene               complement(146..415)
     /gene="lacZalpha"
     /label="lacZalpha gene"
     misc_feature       183..188
     /Recognition_pattern="CA^TATG"
     /annotation_group="CATATG"
     /label="NdeI"
     /note="Geneious type: restriction site"
     misc_feature       245..255
     /Recognition_pattern="GCCNNNN^NGGC"
     /annotation_group="GCCNNNNNGGC"
     /label="BglI"
     /note="Geneious type: restriction site"
     misc_feature       complement(391..415)
     /created_by="User"
     /label="pUC19productionR-Smi"
     /note="Geneious type: primer_bind_reverse"
     misc_feature       396..415
     /gene="lacZalpha"
     /note="multiple cloning site (EcoRI-HindIII)"
     /label="lacZalpha misc"
     misc_feature       396..401
     /Recognition_pattern="G^AATTC"
     /annotation_group="GAATTC"
```

```
/label="[illegible]"
/note="Geneious type: primer_bind_reverse"
misc_feature        396..415
/gene="lacZalpha"
/note="multiple cloning site (EcoRI-HindIII)"
/label="lacZalpha misc"
misc_feature        396..401
/Recognition_pattern="G^AATTC"
/annotation_group="GAATTC"
/label="EcoRI"
/note="Geneious type: restriction site"
primer_bind         401..440
/created_by="User"
/modified_by="User"
/label="T5ko-5'F w/adp"
misc_feature        402..407
/Recognition_pattern="GAGCT^C"
/annotation_group="GAGCTC"
/label="SacI"
/note="Geneious type: restriction site"
misc_feature        412..415
/created_by="User"
/modified_by="User"
/label="SmiI5"
/note="Geneious type: restriction_site"
misc_feature        416..419
/Original_Bases
/label
/note="Geneious type: Editing History Insertion"
misc_feature        416..419
/created_by="User"
/label="Smi3"
/note="Geneious type: restriction_site"
misc_recomb         420..1648
/created_by="User"
/label="5'Recomb"
misc_feature        420..1648
/label="T5ko - 5'Recomb (concatenated sequence 2)"
/note="Geneious type: Concatenated sequence"
primer_bind         420..440
/created_by="User"
/label="T5ko-5'F"
gene                420..805
```

```
/Source="annotation"
/Note="multiprotein bridging factor type 1, putative"
/soTerm="protein_coding"
/product="multiprotein bridging factor type 1, putative"
/taxon="Toxoplasma gondii ME49"
/isDeprecated=0
/isPseudo=0
/label="TGME49_078530"
```

```
UTR                 420..805
/Source="annotation"
/isDeprecated=0
/label="UTR"
```

```
misc_feature        complement(1628..1663)
/created_by="User"
/label="T5ko-5'R w/adp"
/note="Geneious type: primer_bind_reverse"
```

```
misc_feature        complement(1628..1648)
/created_by="User"
/label="T5ko-5'R"
/note="Geneious type: primer_bind_reverse"
```

```
misc_feature        1649..3588
/label="HXGPRT w/ KpnI (concatenated sequence 3)"
/note="Geneious type: Concatenated sequence"
```

```
misc_feature        1649..1654
/created_by="User"
/label="KpnI"
/note="Geneious type: restriction_site"
```

```
primer_bind         1649..1675
/created_by="User"
/label="HXGPRT-KpnIF"
```

```
gene                1655..3584
/created_by="User"
/label="DHFR-HXGPRT"
```

```
misc_feature        complement(1868..1890)
/created_by="User"
/label="5ex2R"
/note="Geneious type: primer_bind_reverse"
```

```
misc_feature        2117..2119
/Original_Bases
/label
/note="Geneious type: Editing History Insertion"
```

```
Selectable_Mark     2123..2815
```

```
     /label="HXGPRT"
     primer               2130..2149
     /note="Oligo predicted by CLC primer designer"
     /note="Oligo type: primer"
     /note="Melting temp.: 56.07"
     /label="HXGPRT detection"
     primer               complement(2845..2866)
     /note="Oligo predicted by CLC primer designer"
     /note="Oligo type: primer"
     /note="Melting temp.: 54.02"
     /label="HXGPRT detection"
     primer_bind          3407..3429
     /created_by="User"
     /label="3exF"
     misc_feature         complement(3568..3588)
     /created_by="User"
     /label="HXGPRT-KpnIR"
     /note="Geneious type: primer_bind_reverse"
     primer_bind          3574..3609
     /created_by="User"
     /label="T5ko-3'F w/adp"
     misc_feature         3583..3588
     /created_by="User"
     /modified_by="User"
     /label="KpnI"
     /note="Geneious type: restriction_site"
     misc_recomb          3589..4806
     /created_by="User"
     /label="3'Recomb"
     misc_feature         3589..4806
     /label="T5ko - 3'Recomb (concatenated sequence 4)"
     /note="Geneious type: Concatenated sequence"
     primer_bind          3589..3609
     /created_by="User"
     /label="T5ko-3'F"
     UTR                  4327..4806
     /Source="annotation"
     /isDeprecated=0
     /label="UTR"
     gene                 4327..4806
     /Source="annotation"
     /Note="protein phosphatase 2C, putative"
```

/soTerm="protein_coding"
/product="protein phosphatase 2C, putative"
/taxon="Toxoplasma gondii ME49"
/isDeprecated=0
/isPseudo=0
/label="TGME49_078510"

---

misc_feature complement(4786..4825)
/created_by="User"
/label="T5ko-3'R w/adp"
/note="Geneious type: primer_bind_reverse"

---

misc_feature complement(4786..4806)
/created_by="User"
/label="T5ko-3'R"
/note="Geneious type: primer_bind_reverse"

---

misc_feature 4807..4810
/Original_Bases
/label
/note="Geneious type: Editing History Insertion"

---

misc_feature 4807..7084
/label="pUC19 3' (concatenated sequence 5)"
/note="Geneious type: Concatenated sequence"

---

misc_feature 4807..4810
/created_by="User"
/label="Smi5"
/note="Geneious type: restriction_site"

---

misc_feature 4811..4850
/gene="lacZalpha"
/note="multiple cloning site (EcoRI-HindIII)"
/label="lacZalpha misc"

---

source 4811..7084
/organism="Cloning vector pUC19"
/mol_type="other DNA"
/label="source Cloning vector pUC19"

CDS complement(4811..4867)
/gene="lacZalpha"
/codon_start=1
/product="beta-galactosidase alpha fragment"
/translation="MTMITPSLHACRSTLEDPRVPSSNSLAVVLQRRDWENPGVTQLNRLAAHPPFASWRNSEEARTD
RPSQQLRSLNGEWRLMRYFLLTHLCGISHRIWCTLSTICSDAA"
/label="lacZalpha CDS"

---

gene complement(4811..4867)
/gene="lacZalpha"

/label="lacZalpha gene"

---

misc_feature 4811..4814
/created_by="User"
/modified_by="User"
/label="SmiI3"
/note="Geneious type: restriction_site"

---

primer_bind 4811..4836
/created_by="User"
/label="pUC19productionF-Smi"

---

misc_feature 4815..4820
/Recognition_pattern="G^GATCC"
/annotation_group="GGATCC"
/label="BamHI"
/note="Geneious type: restriction site"

---

misc_feature 4821..4826
/Recognition_pattern="T^CTAGA"
/annotation_group="TCTAGA"
/label="XbaI"
/note="Geneious type: restriction site"

---

misc_feature 4827..4832
/Recognition_pattern="G^TCGAC"
/annotation_group="GTCGAC"
/label="SalI"
/note="Geneious type: restriction site"

---

misc_feature 4833..4838
/Recognition_pattern="CTGCA^G"
/annotation_group="CTGCAG"
/label="PstI"
/note="Geneious type: restriction site"

---

misc_feature 4845..4850
/Recognition_pattern="A^AGCTT"
/annotation_group="AAGCTT"
/label="HindIII"
/note="Geneious type: restriction site"

-10_signal complement(4912..4917)
/note="Plac promoter (drives lacZalpha;
counter-clockwise); TATGTT"
/label="-10 signal"

-35_signal complement(4936..4941)
/note="Plac promoter (drives lacZalpha;
counter-clockwise); TTTACA"
/label="-35 signal"

```
protein_bind        complement(4961..4973)
/note="CAP protein binding site"
/bound_moiety="catabolite activator protein (CAP)"
/label="CAP protein binding site protein bind"
```

```
rep_origin          complement(5265..5853)
/note="pMB1 origin of replication (counter-clockwise)
(RNAII -35 to RNA/DNA switch point)"
/label="rep origin"
```

```
misc_RNA            complement(5265..5817)
/note="RNAII transcript (complementary strand)"
/label="RNA"
```

```
-35_signal          5671..5676
/note="RNAI promoter (clockwise); TTGAAG"
/label="RNAI promoter (clockwise); TTGAAG -35 signal"
```

```
-10_signal          5693..5698
/note="RNAI promoter (clockwise); GCTACA"
/label="RNAI promoter (clockwise); GCTACA -10 signal"
```

```
misc_RNA            5707..5814
/note="RNAI transcript"
/label="RNAI transcript"
```

```
-10_signal          complement(5827..5832)
/note="RNAII promoter (counter-clockwise); CGTAAT"
/label="-10 signal"
```

```
-35_signal          complement(5848..5853)
/note="RNAII promoter (counter-clockwise); TTGAGA"
/label="-35 signal"
```

```
CDS                 complement(6024..6884)
/gene="bla"
/note="ampR (confers resistance to ampicillin)"
/codon_start=1
/product="beta-lactamase"
/translation="MSIQHFRVALIPFFAAFCLPVFAHPETLVKVKDAEDQLGARVGYIELDLNSGKILESFRPEERF
PMMSTFKVLLCGAVLSRIDAGQEQLGRRIHYSQNDLVEYSPVTEKHLTDGMTVRELCSAAITMSDNTAANLLLTTIGG
PKELTAFLHNMGDHVTRLDRWEPELNEAIPNDERDTTMPVAMATTLRKLLTGELLTLASRQQLIDWMEADKVAGPLL
RSALPAGWFIADKSGAGERGSRGIIAALGPDGKPSRIVVIYTTGSQATMDERNRQIAEIGASLIKHW"
/label="bla CDS"
```

```
gene                complement(6024..6884)
/gene="bla"
/label="bla gene"
```

```
misc_feature        6211..6221
/Recognition_pattern="GCCNNNN^NGGC"
/annotation_group="GCCNNNNNGGC"
```

```
      /label="BglI"
      /note="Geneious type: restriction site"
      sig_peptide         complement(6816..6884)
      /gene="bla"
      /note="required for secretion to the periplasm; cleaved
      off to form the mature beta-lactamase protein."
      /label="bla signal peptide"
      -10_signal          complement(6928..6933)
      /note="bla promoter (counter-clockwise); GAGACA"
      /label="-10 signal"
      -35_signal          complement(6949..6954)
      /note="bla promoter (counter-clockwise); TTCAAA"
      /label="-35 signal"

        10        20        30        40
tcgcgcgtttcggtgatgacggtgaaaacctctgacacat 40
gcagctcccggagacggtcacagcttgtctgtaagcggat 80
gccgggagcagacaagcccgtcagggcgcgtcagcgggtg 120
ttggcgggtgtcggggctggcttaactatgcggcatcaga 160
gcagattgtactgagagtgcaccatatgcggtgtgaaata 200
       210       220       230       240
ccgcacagatgcgtaaggagaaaataccgcatcaggcgcc 240
attcgccattcaggctgcgcaactgttgggaagggcgatc 280
ggtgcgggcctcttcgctattacgccagctggcgaaaggg 320
ggatgtgctgcaaggcgattaagttgggtaacgccagggt 360
tttcccagtcacgacgttgtaaaacgacggccagtgaatt 400
       410       420       430       440
cgagctcggtaatttaaattgcggttcatccacgcagaga 440
caccggctcttgggcggacgttgttttcgccgttttcgct 480
ggtttcccattcatttgtcgacaatgtgtttcgaccgcat 520
tcaccgataccatttgtgcatggagcaaagcgactaggcg 560
cctccgcgtctccagagaaccttgtgtgtagtgatcgtga 600
       610       620       630       640
acacagaggttcggtttccagctgtccgacatctgttggg 640
tgactgacttcatgctgtgcaataatgcattcagcgacat 680
agtgttggttcgctggtaacatctaagtccttcattgtgg 720
cggaaaatattcatatcgcgtcggcatgacacgtccagaa 760
caaggtgcatacatgcagactgacacagaagaatttgca 800
       810       820       830       840
gatcgattccttgtcgacacacggcaggcactgcatacct 840
cgagacgatggcactgagaagcgcctcttgggatcacgga 880
aaagtcctagtggagatagaccgtctgcagactgtggtgt 920
tcccttcatgctacgcagacctgcgtcacaacgcttcagg 960
aatactgttggcctgagtcgaagaccgtcactgtactgga 1000
```

```
        1010      1020      1030      1040
tatagagaggctgagcaaattatttgcgtgcataacagcc 1040
tgacgatggtttattgcagtatatctgcggtcgctggtca 1080
gcccgacatttgtataggaaagtcctttatcaggctggaa 1120
cgcttttcctgttttcgtcatccgtgaccggaccgggcag 1160
ctgaacagggagtgaaagaattttatggcagattatcctt 1200
        1210      1220      1230      1240
cagatctacctaccttgtccacactgtcatgaaagccgac 1240
gtcgtcagcattttactagttcatatccgatcgcttggta 1280
gctgagggtttgaaatgtgtgcatttagactatacaacac 1320
agtttgccgtgttcgtccactctcaatactggaaacggat 1360
agtggtcggatttcatgtgtcattcgtacttgtaacacat 1400
        1410      1420      1430      1440
cggaataaaatttagtcgattcatgctccaggacattatc 1440
ggagctcttcaaaggggctggcccacacgctgcctcagcc 1480
ccacatgtggaagacgttcattttcgagtatttcgttga 1520
ggcggaaagcgagtcccgcccacaccgcttgtcgaaaaga 1560
ggcctgccggagttgtcatcgctatcgaggaactccgata 1600
        1610      1620      1630      1640
tcgttgttatccgcccttattctcttcaccccactcaaca 1640
cagctggaggtaccttgatcagcacgaaaccttgcattca 1680
aacccgcccgcggaagatccgatcttgctgctgttcgcag 1720
tcccagtagcgtcctgtcggccgcgccgtctctgttggtg 1760
ggcagccgctacacctgttatctgactgccgtgcgcgaaa 1800
        1810      1820      1830      1840
atgacgccattttgggaaaatcggggaacttcattcttt 1840
aaaagtatgcggaggtttccttttcttctgttcgtttct 1880
ttttctcgggtttgataaccgtgttcgatgtaagcacttt 1920
ccgtctctcctccgtgctttgttcgacatcgagaccaggt 1960
gtgcagatccttcgcttgtcgatccggagacgcgtgtctc 2000
        2010      2020      2030      2040
gtagaacctttcatttaccacacggcagtgcggagcac 2040
tgctctgagtgcagcagggacgggtgaagtttcgctttag 2080
tagtgcgtttctgctctacggggcgttgtcagatccagca 2120
aaatggcgtccaaacccattgaagactacggcaagggcaa 2160
gggccgtattgagcccatgtatatccccgacaacaccttc 2200
        2210      2220      2230      2240
tacaacgctgatgactttcttgtgccccccactgcaagc 2240
cctacattgacaaaatcctcctccctggtggattggtcaa 2280
ggacagagttgagaagttggcgtatgacatccacagaact 2320
tacttcggcgaggagttgcacatcatttgcatcctgaaag 2360
gctctcgcggcttcttcaaccttctgatcgactaccttgc 2400
```

```
        2410      2420      2430      2440
caccatacagaagtacagtggtcgtgagtccagcgtgccc 2440
cccttcttcgagcactatgtccgcctgaagtcctaccaga 2480
acgacaacagcacaggccagctcaccgtcttgagcgacga 2520
cttgtcaatctttcgcgacaagcacgttctgattgttgag 2560
gacatcgtcgacaccggtttcaccctcaccgagttcggtg 2600
        2610      2620      2630      2640
agcgcctgaaagccgtcggtcccaagtcgatgagaatcgc 2640
caccctcgtcgagaagcgcacagatcgctccaacagcttg 2680
aagggcgacttcgtcggcttcagcattgaagacgtctgga 2720
tcgttggttgctgctacgacttcaacgagatgttccgcga 2760
cttcgaccacgtcgccgtcctgagcgacgccgctcgcaaa 2800
        2810      2820      2830      2840
aagttcgagaagtaaaccctgcatagccctacagaagctg 2840
cccgtctctcgttttcctctcttttcggagggatcaggga 2880
gagtgcctcgggtcggagagagctgacgagggggtgccag 2920
agacccctgtgtcctttatcgaagaaaagggatgactctt 2960
catgtggcatttcacacagtctcacctcgccttgttttct 3000
        3010      3020      3030      3040
ttttgtcaatcagaacgaaagcgagttgcgggtgacgcag 3040
atgtgcgtgtatccactcgtgaatgcgttatcgttctgta 3080
tgccgctagagtgctggactgttgctgtctgcccacgaca 3120
gcagacaactttccttctatgcacttgcaggatggtgcag 3160
cgcaaacgacggagagaaaggagcaccctctcagtttccc 3200
        3210      3220      3230      3240
tacgatgtgctgtcagtttcgactcttcaccgcgaacgat 3240
tggcgatacgtctctgttgacttgttaggctccgaccacg 3280
aagctcccttaactaaataagccgcgacacctaagtgtac 3320
accatttgcagatcgataatctgcgaccgctgaatccgtc 3360
cagatcaggaaaaccgcaccacctaagtgtaaaccttgtt 3400
        3410      3420      3430      3440
taggtcgataaaatgctaccaacccccacccacaatcgag 3440
ccttgagcgtttctgcgcacgcgttggcctacgtgacttg 3480
ctgatgcctgcctctggccattcatgccagtcagtgcgca 3520
taaaaatgtggacacagtcggttgacaagtgttctggcag 3560
gctacagtgacaccgcggtggaggtaccaatttgggcttt 3600
        3610      3620      3630      3640
cctgcgggctatgaaaggataattcccacgttttactatg 3640
gagaaaagtatgcaaacagccatccggtcattgtacatct 3680
ggtgtagcagaggttgaagaacaaaagcaacactataccg 3720
tacaggggattgctgtagccatgctcagacattcccttgt 3760
ctccgctatgtctctttctcttcatctacttctctaacgt 3800
```

```
        3810      3820      3830      3840
aaaccatcaagagttgcctgccacctctgttacaggattt 3840
gccaagtgcaacgggaatgttttgatttgccatttgcttt 3880
ctctaacaggaacacaggtcaaattatgttggacgcaaaa 3920
acaattagcggtgacgcgctttgcatcggctatccggatc 3960
gcgttttcttgtgcgaggacgagaaacggacgagaaattt 4000
        4010      4020      4030      4040
ctctgtggaaattcgtcagaacggcttggtagtttggacc 4040
gattccccggaaacaagacaggctaaagcttgcttgacgc 4080
tagtcgacgcccacaagttttcaagtccttctcaggtacg 4120
tggcataacaaaacgaattttaaaacgaaggagaaagctg 4160
tggggtatgcatagcgtgagaaaagagcgagagctccaaa 4200
        4210      4220      4230      4240
agtagttgaatccgactgccagagaagagccgtgagacac 4240
gcgcgagggacgggcatctggaaccggtgaaataagtgtc 4280
acaagcacgcaggaagttttctcgtagctcatgcgactca 4320
cggtgagcgcagataagatactttgtacacaacagctaac 4360
attatctcttgttcgctgtcttcggaattgtttatttctt 4400
        4410      4420      4430      4440
cttgttagggttttgttcgtttcgaggcttgcgccgagac 4440
tgccaacgatgctagccagctctggattgtgaaggagata 4480
caaaacggcagagatatgacctcttcgtagcaacgtctac 4520
gcgaaataacaagtatttttgcctttggaacagttgcaga 4560
aggcagcgtgtatggccgagcgaagtgtgatcagccgttg 4600
        4610      4620      4630      4640
acgcttgttcttgaatgcctcttaaggttttgagtgaccc 4640
accaggggaagcttcccttccctaaaccaccagcagcca 4680
ccccttcccattgtctgcgcagactttcttacagcttcca 4720
ctggggtcttgttactcgtgcaattcagttatgtacgtga 4760
ataaattaagtgttcatgtgatcaacgcaatatgcacgtg 4800
        4810      4820      4830      4840
acgaccatttaaatggatcctctagagtcgacctgcaggc 4840
atgcaagcttggcgtaatcatggtcatagctgtttcctgt 4880
gtgaaattgttatccgctcacaattccacacaacatacga 4920
gccggaagcataaagtgtaaagcctggggtgcctaatgag 4960
tgagctaactcacattaattgcgttgcgctcactgcccgc 5000
        5010      5020      5030      5040
tttccagtcgggaaacctgtcgtgccagctgcattaatga 5040
atcggccaacgcgcggggagaggcggtttgcgtattgggc 5080
gctcttccgcttcctcgctcactgactcgctgcgctcggt 5120
cgttcggctgcggcgagcggtatcagctcactcaaaggcg 5160
gtaatacggttatccacagaatcaggggataacgcaggaa 5200
```

```
        5210      5220      5230      5240
agaacatgtgagcaaaaggccagcaaaaggccaggaaccg 5240
taaaaaggccgcgttgctggcgtttttccataggctccgc 5280
cccccctgacgagcatcacaaaaatcgacgctcaagtcaga 5320
ggtggcgaaacccgacaggactataaagataccaggcgtt 5360
tcccccctggaagctccctcgtgcgctctcctgttccgacc 5400
        5410      5420      5430      5440
ctgccgcttaccggatacctgtccgcctttctcccttcgg 5440
gaagcgtggcgctttctcatagctcacgctgtaggtatct 5480
cagttcggtgtaggtcgttcgctccaagctgggctgtgtg 5520
cacgaaccccccgttcagcccgaccgctgcgccttatccg 5560
gtaactatcgtcttgagtccaacccggtaagacacgactt 5600
        5610      5620      5630      5640
atcgccactggcagcagccactggtaacaggattagcaga 5640
gcgaggtatgtaggcggtgctacagagttcttgaagtggt 5680
ggcctaactacggctacactagaagaacagtatttggtat 5720
ctgcgctctgctgaagccagttaccttcggaaaaagagtt 5760
ggtagctcttgatccggcaaacaaaccaccgctggtagcg 5800
        5810      5820      5830      5840
gtggtttttttgtttgcaagcagcagattacgcgcagaaa 5840
aaaaggatctcaagaagatcctttgatcttttctacgggg 5880
tctgacgctcagtggaacgaaaactcacgttaagggattt 5920
tggtcatgagattatcaaaaaggatcttcacctagatcct 5960
tttaaattaaaaatgaagttttaaatcaatctaaagtata 6000
        6010      6020      6030      6040
tatgagtaaacttggtctgacagttaccaatgcttaatca 6040
gtgaggcacctatctcagcgatctgtctatttcgttcatc 6080
catagttgcctgactccccgtcgtgtagataactacgata 6120
cgggagggcttaccatctggccccagtgctgcaatgatac 6160
cgcgagacccacgctcaccggctccagatttatcagcaat 6200
        6210      6220      6230      6240
aaaccagccagccggaagggccgagcgcagaagtggtcct 6240
gcaactttatccgcctccatccagtctattaattgttgcc 6280
gggaagctagagtaagtagttcgccagttaatagtttgcg 6320
caacgttgttgccattgctacaggcatcgtggtgtcacgc 6360
tcgtcgtttggtatggcttcattcagctccggttcccaac 6400
        6410      6420      6430      6440
gatcaaggcgagttacatgatcccccatgttgtgcaaaaa 6440
agcggttagctccttcggtcctccgatcgttgtcagaagt 6480
aagttggccgcagtgttatcactcatggttatggcagcac 6520
tgcataattctcttactgtcatgccatccgtaagatgctt 6560
ttctgtgactggtgagtactcaaccaagtcattctgagaa 6600
```

```
        6610      6620      6630      6640
tagtgtatgcggcgaccgagttgctcttgcccggcgtcaa 6640
tacgggataataccgcgccacatagcagaactttaaaagt 6680
gctcatcattggaaaacgttcttcggggcgaaaactctca 6720
aggatcttaccgctgttgagatccagttcgatgtaaccca 6760
ctcgtgcacccaactgatcttcagcatcttttactttcac 6800
        6810      6820      6830      6840
cagcgtttctgggtgagcaaaaacaggaaggcaaaatgcc 6840
gcaaaaaagggaataagggcgacacggaaatgttgaatac 6880
tcatactcttcctttttcaatattattgaagcatttatca 6920
gggttattgtctcatgagcggatacatatttgaatgtatt 6960
tagaaaaataaacaaataggggttccgcgcacatttcccc 7000
        7010      7020      7030      7040
gaaaagtgccacctgacgtctaagaaaccattattatcat 7040
gacattaacctataaaaataggcgtatcacgaggcccttt 7080
cgtc 7084
```

```
LOCUS       pT2CompDHFRv2           9646 bp
        26-MAY-2015
EMBL_ID     pT2CompDHFRv2           9646 bp
        26-FEB-2015
EMBL_ID     pT2CompDHFRv2           9646 bp
        11-FEB-2015
EMBL_ID     pT2CompDHFRv2           9646 bp
        15-JAN-2015
EMBL_ID     pT2CompDHFRv2           9646 bp
        12-DEC-2014
EMBL_ID     pT2CompDHFRv2           9646 bp
        12-DEC-2014
EMBL_ID     pT2CompDHFRv2           9646 bp
        30-DEC-2014
EMBL_ID     pT2CompDHFRv2           9646 bp
        11-FEB-2015
EMBL_ID     pT2CompDHFRv2           9646 bp
        20-FEB-2015
EMBL_ID     pT2CompDHFRv2           9646 bp
        14-APR-2015
UNIMARK     pT2CompDHFRv2 annotations
            pT2CompDHFRv2



ORIGIN
Features
     source             1..415
     /organism="Cloning vector pUC19"
     /mol_type="other DNA"
     /label="source Cloning vector pUC19"
     source             1..9646
     /comment="Molecule is created with Unipro UGENE v1.13"
     CDS                complement(146..415)
     /gene="lacZalpha"
     /codon_start=1
     /product="beta-galactosidase alpha fragment"
     /translation="MTMITPSLHACRSTLEDPRVPSSNSLAVVLQRRDWENPGVTQLNRLAAHPPFASWRNSEEARTDRPSQQLRSLNGEWRLMRYFLLTHLCGISHR[illegible]
     STICSDAA"
     /label="lacZalpha CDS"
     primer_bind        complement(391..415)
     /created_by="User"
     /label="pUC19productionR-Smi"
     /note="Geneious type: primer_bind_reverse"
     misc_feature       396..415
     /gene="lacZalpha"
     /note="multiple cloning site (EcoRI-HindIII)"
     /label="lacZalpha misc"
     primer bind        401..440
```

```
misc_feature        396..415
/gene="lacZalpha"
/note="multiple cloning site (EcoRI-HindIII)"
/label="lacZalpha misc"
```

```
primer_bind         401..440
/created_by="User"
/label="T2ko-5'F w/adp"
```

```
Site                412..419
/label="SwaI"
```

```
misc_recomb         420..1336
/created_by="User"
/label="T2-5'Recomb"
```

```
primer_bind         420..440
/created_by="User"
/label="T2ko-5'F"
```

```
primer_bind         420..437
/mismatches=0
```

```
promoter            420..1336
/label="T2 promoter Pru"
/ugene_group="misc_recomb"
```

```
primer_bind         1212..1231
/label="T2comp-coloF"
```

```
primer_bind         complement(1315..1336)
/created_by="User"
/label="T2ko-5'R"
```

```
primer_bind         1347..1366
/label="T2CompSeqF"
```

```
primer_bind         complement(1369..1401)
/label="T2-5p-RecombR"
```

```
primer_bind         1385..1411
/label="T2CDScompF"
```

```
misc_feature        1391..1393
/label="Start"
```

```
CDS                 1394..3466
/note="Geneious type: Concatenated sequence"
/label="T2_cds"
```

```
exon                1394..1748
/label="Ex1"
```

```
primer_bind         1500..1521
/created_by="User"
/label="T2colonyF"
```

```
primer_bind         1504..1521
/note="Oligo predicted by CLC primer designer"
```

```
     /note="Oligo type: primer"
     /note="Melting temp.: 59.08"
     /label="T2F3"
     Region              1511..1579
     /label="Transmembrane"
     primer_bind         1575..1592
     /label="T2F wo TM"
     exon                1749..1882
     /label="Ex2"
     primer_bind         complement(1764..1783)
     /label="T2comp-coloR"
     Region              1847..2722
     /label="Transferase"
     exon                1883..2045
     /label="Ex3"
     misc_feature        complement(2034..2055)
     /created_by="User"
     /label="T2colonyR"
     /note="Geneious type: primer_bind_reverse"
     exon                2046..2212
     /label="Ex4"
     exon                2213..2372
     /label="Ex5"
     exon                2373..2536
     /label="Ex6"
     exon                2537..2777
     /label="Ex7"
     Region              2741..3016
     /label="Ricin B lectin"
     exon                2778..2863
     /label="Ex8"
     exon                2864..3466
     /label="Ex9"
     primer_bind         complement(3306..3326)
     /note="Oligo predicted by CLC primer designer"
     /note="Oligo type: primer"
     /note="Melting temp.: 59.17"
     /label="T2R3"
     misc_feature        3313..4543
     /mismatches=0
     /ugene_name="verified"
     primer_bind         complement(3450..3466)
```

/mismatches=0	
primer_bind	complement(3450..3498)
/label="T2CDScompR"	
misc_feature	3467..3472
/label="GAlinker"	
primer_bind	complement(3472..3510)
/mismatches=0	
/label="T2CDSr-83-2"	
misc_feature	3473..3496
/mismatches=0	
/label="FLAG"	
/ugene_name="Tag"	
primer_bind	3491..3517
/mismatches=0	
/label="SAG1-3'UTRf"	
misc_feature	3497..3499
/label="Stop"	
3'UTR	3500..3812
/label="SAG1 3'UTR"	
misc_difference	3587..3587
/label="C->T"	
primer_bind	complement(3806..3828)
/mismatches=0	
/label="SAG1-3'UTRr"	
primer_bind	3812..3837
/mismatches=0	
/label="TUBp-loxPf-83-2"	
promoter	3826..4358
/label="P5RT70 prmtr"	
primer_bind	3832..3851
/mismatches=0	
promoter	complement(3852..3984)
/label="SAG1 promoter"	
promoter	3991..4309
/label="alpha tubulin TUBA1 promoter"	
misc_difference	4139..4140
/label="CC->GG"	
/ugene_group="misc_feature"	
misc_difference	4181..4183
/label="GTG->CTC"	
misc_feature	4310..4343
/ugene_name="loxP"	

```
primer_bind         complement(4324..4363)
/mismatches=0
/label="TUBp-loxPr"
```

```
primer_bind         4344..4375
/mismatches=0
/label="DHFR-loxP-F"
```

```
CDS                 4359..6191
/mismatches=0
/label="DHFR"
```

```
misc_feature        4359..6191
/source_doc="pT2CompDHFR.gb"
/label="DHFR from HA DHFR"
/ugene_name="fragment"
```

```
primer_bind         complement(6177..6216)
/label="DHFR-loxP-R"
```

```
misc_feature        6192..6225
/mismatches=0
/ugene_name="loxP"
```

```
primer_bind         6201..6240
/label="pUC19wUPRT-vecF"
```

```
misc_feature        6226..9646
/source_doc="pT2CompDHFR.gb"
/label="pUC19-T2KOvector"
/ugene_name="fragment"
```

```
misc_recomb         6232..7368
/created_by="User"
/modified_by="User"
/label="T2-3'Recomb"
```

```
primer_bind         6232..6252
/created_by="User"
/label="T2ko-3'F"
```

```
3'UTR               6232..6795
/label="T2-3'UTR"
/ugene_group="UTR"
```

```
primer_bind         complement(6796..6815)
/ugene_group="gene"
```

```
primer_bind         complement(7348..7387)
/created_by="User"
/label="T2ko-3'R w/adpt"
/note="Geneious type: primer_bind_reverse"
```

```
primer_bind         complement(7348..7368)
/created_by="User"
```

```
/label="T2ko-3'R"
```

```
Site                 7369..7376
/label="SmiI"
```

```
misc_feature         7373..7412
/gene="lacZalpha"
/note="multiple cloning site (EcoRI-HindIII)"
/label="lacZalpha misc"
```

```
source               7373..9646
/organism="Cloning vector pUC19"
/mol_type="other DNA"
/label="source Cloning vector pUC19"
```

```
CDS                  complement(7373..7429)
/gene="lacZalpha"
/codon_start=1
/product="beta-galactosidase alpha fragment"
/translation="MTMITPSLHACRSTLEDPRVPSSNSLAVVLQRRDWENPGVTQLNRLAAHPPFASWRNSEEARTD
RPSQQLRSLNGEWRLMRYFLLTHLCGISHRIWCTLSTICSDAA"
/label="lacZalpha CDS"
```

```
gene                 complement(7373..7429)
/gene="lacZalpha"
/label="lacZalpha gene"
```

```
primer_bind          7373..7398
/created_by="User"
/label="pUC19productionF-Smi"
```

```
-10_signal           complement(7474..7479)
/note="Plac promoter (drives lacZalpha;
counter-clockwise); TATGTT"
/label="-10 signal"
```

```
-35_signal           complement(7498..7503)
/note="Plac promoter (drives lacZalpha;
counter-clockwise); TTTACA"
/label="-35 signal"
```

```
protein_bind         complement(7523..7535)
/note="CAP protein binding site"
/bound_moiety="catabolite activator protein (CAP)"
/label="CAP protein binding site protein bind"
```

```
rep_origin           complement(7827..8415)
/note="pMB1 origin of replication (counter-clockwise)
(RNAII -35 to RNA/DNA switch point)"
/label="rep origin"
```

```
misc_RNA             complement(7827..8379)
/note="RNAII transcript (complementary strand)"
```

/label="RNA"

-35_signal 8233..8238
/note="RNAI promoter (clockwise); TTGAAG"
/label="RNAI promoter (clockwise); TTGAAG -35 signal"

-10_signal 8255..8260
/note="RNAI promoter (clockwise); GCTACA"
/label="RNAI promoter (clockwise); GCTACA -10 signal"

misc_RNA 8269..8376
/note="RNAI transcript"
/label="RNAI transcript"

-10_signal complement(8389..8394)
/note="RNAII promoter (counter-clockwise); CGTAAT"
/label="-10 signal"

-35_signal complement(8410..8415)
/note="RNAII promoter (counter-clockwise); TTGAGA"
/label="-35 signal"

CDS complement(8586..9446)
/gene="bla"
/note="ampR (confers resistance to ampicillin)"
/codon_start=1
/product="beta-lactamase"
/translation="MSIQHFRVALIPFFAAFCLPVFAHPETLVKVKDAEDQLGARVGYIELDLNSGKILESFRPEERF
PMMSTFKVLLCGAVLSRIDAGQEQLGRRIHYSQNDLVEYSPVTEKHLTDGMTVRELCSAAITMSDNTAANLLLTTIGG
PKELTAFLHNMGDHVTRLDRWEPELNEAIPNDERDTTMPVAMATTLRKLLTGELLTLASRQQLIDWMEADKVAGPLL
RSALPAGWFIADKSGAGERGSRGIIAALGPDGKPSRIVVIYTTGSQATMDERNRQIAEIGASLIKHW"
/label="bla CDS"

gene complement(8586..9446)
/gene="bla"
/label="bla gene"

sig_peptide complement(9378..9446)
/gene="bla"
/note="required for secretion to the periplasm; cleaved
off to form the mature beta-lactamase protein."
/label="bla signal peptide"

-10_signal complement(9490..9495)
/note="bla promoter (counter-clockwise); GAGACA"
/label="-10 signal"

-35_signal complement(9511..9516)
/note="bla promoter (counter-clockwise); TTCAAA"
/label="-35 signal"

```
        10        20        30        40
TCGCGCGTTTCGGTGATGACGGTGAAAACCTCTGACACAT 40
GCAGCTCCCGGAGACGGTCACAGCTTGTCTGTAAGCGGAT 80
GCCGGGAGCAGACAAGCCCGTCAGGGCGCGTCAGCGGGTG 120
TTGGCGGGTGTCGGGGCTGGCTTAACTATGCGGCATCAGA 160
GCAGATTGTACTGAGAGTGCACCATATGCGGTGTGAAATA 200
       210       220       230       240
CCGCACAGATGCGTAAGGAGAAAATACCGCATCAGGCGCC 240
ATTCGCCATTCAGGCTGCGCAACTGTTGGGAAGGGCGATC 280
GGTGCGGGCCTCTTCGCTATTACGCCAGCTGGCGAAAGGG 320
GGATGTGCTGCAAGGCGATTAAGTTGGGTAACGCCAGGGT 360
TTTCCCAGTCACGACGTTGTAAAACGACGGCCAGTGAATT 400
       410       420       430       440
CGAGCTCGGTAATTTAAATACTCTCCTCGCTGCGGTTCTT 440
TCCTTCTTAAGAGCATCCGTTGTCGCGTCCTGACCCTCTC 480
CCCCCCCACCCTCCGCGAGAACGGGGAAAACCTGAACCCT 520
CTGAGAAATGAAGCGCCTCTCGTCAGACGCGGCGACTCGC 560
CACGGACTTTCCAGAAAAGTCAACGCGTGGAACTGAGGGC 600
       610       620       630       640
CGCTCTCGAAGGCTCTCTGCCCTGCACCTTGAGCCCAGAT 640
GTATTGCGAAGGAAGGCCTGCCGCACTGCCTCGTCTCGAC 680
AAGGTTGTCTCCTGCGCCGCCGGGACTCGACTAAGAAGAA 720
TCTTCGCAAACGCTTCACACTCCTCATGTGCTCGAGTTCG 760
GTCCTCGTTCCCAGGCTCCTCGTTCCTCTCTTCCCCTGTC 800
       810       820       830       840
TCCGCGGGCTCTTCGCGTCCACCGAAAAGCGGTCTTCCTC 840
CTCAAGAAAACGGATCGCCTTTCTGTGGGGAACATCCGTG 880
TTCTCCCGCTCGGCGTGTGAACTAGGAAGCTCGCAGCGTC 920
TCAACTTCTTAAATCTATTTACATTTTAAGTGTAATCATA 960
TATGCATATTTGTGTTTAAATAAGGTAAACATAACTGTAT 1000
      1010      1020      1030      1040
ACAGATTTATACTATAACCATAAGCATAAATACACATATA 1040
GATATATGTATACGTATATGTCTCAGCGCCGTTGAATCCC 1080
CTTCCTGGTGTCCGTCCTGGGAACTGTCGAACTTCTCTTG 1120
GTGGGGGATTGTGGGTCGGCGGCGCCTACGCTCTCAACTC 1160
GCTTCTGTCCTCTTCTCGGTTCTTCTGGAGTTCTGATGGT 1200
      1210      1220      1230      1240
TCCACAAGGACTCCCCGTTACCATTTCGTCCGCTCTTTGA 1240
CTTCGTTCACTTCCTCTCTGCTGGGCAAGCGATACGTGAA 1280
GGCCCTGGCCTAGGCTGCCATTGTTCTCTCTCCTCGTTCG 1320
TTCCCGAGGAGCCTTATTTCTCTCCGTCTTGGCCTCTGTT 1360
CTGGTGCTTCGCCTTCCATTCTCGTCTACTATGCCGGTCG 1400
```

```
        1410      1420      1430      1440
AGTGCCCAGTGGGAGGCGGCCAGGAGACGCCGCGAGCCTC 1440
GTGGCGGAGGGGAGAGCCGGGCAAGAAGCCGCTGAACTCG 1480
AAGCTGGTCGCGCGGCTGCGGCGTCGGTGTTTGCTTCTGC 1520
TCGGATCGCTCTGCGTGAGTGTGGGGATTTTCTGGTGTCT 1560
CTTCTCCTCGGGAGTTCTCACGCTGCTCTTCCCCGGCGCC 1600
        1610      1620      1630      1640
TTCAGCCGCGACTCGCCTCAGACGCACTCCCTGGACTTGC 1640
ATGCACCTGCAGTCGCCGAACTCCACAGCCTCCTCAAAGG 1680
CCTGCCCCTCCCGACGCACCACGAAAAAGCCACCGTCGAC 1720
AACAGCGCAATCGTCGTCGGCGTCCACGGCACAGTGAAGG 1760
CACCGTACAGCAGAGTCGTCGACCATCTGTTGAAGATCGT 1800
        1810      1820      1830      1840
GGTACTTCCGGTGTTGACAGGAGAGCCGCGTTACGTGACT 1840
GGAGACGATTTCTCAGTTATCATTCCTGTTCGCAATGAAG 1880
AGGCGTATCTTCCCAAGACGTTGACTTTCTTGTTCGAAGT 1920
AACTCCAATCGAGCGCATCCGCGAGGTGATTGTCGTCGAC 1960
GACCACAGCGACAAGCCGATAAAAGCAATTCTGGAGAAGA 2000
        2010      2020      2030      2040
CGCTTCCTCAACACATCCTCGACAAGACGCGCATCATCCG 2040
CTTCGACTCGCCTCAAGGCTTAATTCGCGGACGGGTGGCG 2080
GGAGCAGCGATTGCGACAAGCGACAACTTTTTCTTTCTCG 2120
ACGGACACTGCAGACCGAAGGTCGGCTGGGCGGAGCCTCT 2160
CCTCGCGCATTTGAAAACCAATTACCGGAGAATTGCTTGC 2200
        2210      2220      2230      2240
CCCAAGATTTACGATATTTACCTGGACTCCTGGGAAGACG 2240
TCGGCACCCACGGAACGAAGATGATGTTTGAGTGGACTTT 2280
CGAATTCGGCTGGTTTGAAGACCTCGAAGACGAAGTCCCC 2320
GTGCTTGCAGGCGGCATTCTCGCGATGACTAAGAAGTGGT 2360
GGATTGAGTCCGGGCTGTACGACGAAGGCATGCTCGAGTG 2400
        2410      2420      2430      2440
GGGTGGAGAAAATCTGGAGCAGTCGATTCGCTCCTGGCTC 2440
TGCGGAGGCGAAATCGTGGCCGTGCAGGAGTCAAAAATCG 2480
GACACATTTTCTCGAGGCCTCCGAAACCGAATCCAGGAAA 2520
TCGACTTGTCATACAGGTCCAGAAGAATCAGAAACGCGGA 2560
GCGAAAGTCTGGCTGGACGAGTACTACTTTTTATTCTACA 2600
        2610      2620      2630      2640
AGTACCACCGGGAAGTGCGTGGACACCAGGAAGGCGACAT 2640
TACGCAGCGAAAGAAACTGCGGTACGAACAGTTGACTTGC 2680
ATGCCATTCCAGTGGTACGTGGAGAAATTCAAAACTGCCT 2720
TCGACCGCAAGGGACTTCTCGACCACAACTTCTTCCACAT 2760
TGAACACGAACAAACAGGCCTGTGTCTCTCCTCTGCGTAC 2800
```

```
        2810      2820      2830      2840

ACCCCCGGTTTTCCCGACCACCGTCTCGTGTTGGAGCCTT 2840
GCGATCTCCACGCCCGTCTCCAGCAATGGACCGTGGCCGC 2880
GGGGAATCGGTTGATTGAAAACCGAGAGAAGAAGTGCATC 2920
GACGCGTTTGTCCAGCAAGCCGATGCGCGGGCGCCGGTTG 2960
TGTGGACCTGCGACTGGAACCGCGTCCTGCAAAGGCAAAA 3000

        3010      3020      3030      3040

CGAAAACCAGTTCTGGCAGTGGGATGCGTCGCTGCCGGGC 3040
CAGGCTGCCTCTGCAAACGCGACGGGACGAATCTTCTCCT 3080
TCTCGCACCCCGACACTGTCTCCCTCACGGGTCACGACAC 3120
GCTGTCGGTGTACAGACACCTGGCGGCGTCGCACCATGCG 3160
CGGTGCCTGGTGGTGGAGGCGCCGCGGAGCCGCGGAGACG 3200

        3210      3220      3230      3240

AGCAGAAGAAGCTCCTTTTCGTGAACTGGAAGAGCTGCGA 3240
CGAGCCCGTCCGTCCGGAGACGAACGACGGGTGGACCGTC 3280
GAGAACCGCGGAGAGGGGCAGGACGAGGAAGAAGAAGTAC 3320
GAGGAGACCGCATGCGCTTCAAGCCAAAGTGGTTCTTCGC 3360
GCCGCTCTCGTCCAGAGTCGGCCTGCGCACGATGGAGGAC 3400

        3410      3420      3430      3440

GCTAACAGCATCACCGACCAAGCCGCAGAAAAGTACCTCA 3440
GAAAGGCGCCGAAGCCGCTCGACGAGGGAGCTGATTACAA 3480
GGATGACGACGATAAGTAGATCACCGTTGTGCTCACTTCT 3520
CAAATCGACAAAGGAAACACACTTCGTGCAGCATGTGCCC 3560
CATTATAAAGAAACTGAGTTGTTCCGTTGTGGCTTGCAGG 3600

        3610      3620      3630      3640

TGTCACATCCACAAAAACCGGCCGACTCTAAATAGGAGTG 3640
TTTCGCAGCAAGCAGCGAAAGTTTATGACTGGGTCCGAAT 3680
CTCTGAACGGATGTGTGGCGGACCTGGCTGATGTTGATCG 3720
CCGTCGACACACGCGCCACATGGGTCAATACACAAGACAG 3760
CTATCAGTTGTTTTAGTCGAACCGGTTAACACAATTCTTG 3800

        3810      3820      3830      3840

CCCCCCCGAGGGGGATCGATAAGCTCCCCCCTCGACGGTA 3840
TCGATAAGCTTGAACACGCGTCTCAGGGTGCGTGGTTAGA 3880
ACACGCGTCTCAGGGTGCGTGGTTAGAACACGCGTCTCAG 3920
GGTGCGTGGTTGGAACACGCGTGTCAGGGTGCGTGGTTGG 3960
AACACGCGTCTCAAGGTTTGTGGTTAAGCTTGCCTGCATT 4000

        4010      4020      4030      4040

GGGTGCGGTTGGTGATCCTGGTTGGACCGGTGGAGATGCG 4040
CGCGCACGAAGGGGATGTGTCAGAAACATTTTGTTTGTTC 4080
TCTGTGAACTTTTAGATGTGTTAAAGGCGGCGAATATTAG 4120
CAGAGAGTCCTCCTTGTTGGATTCTCTCTTGAATTTCGCC 4160
CTTTCCTTCTCTTTGCGAGTCTCGTAGAGAACAAGCACTC 4200
```

```
        4210      4220      4230      4240
GTTCGCCGTCCCTGACGACGCAACCCGCGCAGAAGACATC 4240
CACCAAACGGTGTTACACAATCACCTTGTGTGAAGTTCTT 4280
GCGGAAAACTACTCGTTGGCATTTTTTCTATAACTTCGTA 4320
TAGCATACATTATACGAAGTTATAGAATTCCGACAAAAAT 4360
GCAGAAACCGGTGTGTCTGGTCGTCGCGATGACCCCCAAG 4400
        4410      4420      4430      4440
AGGGGCATCGGCATCAACAACGGCCTCCCGTGGCCCCACT 4440
TGACCACAGATTTCAAACACTTTCGTCGTGTGACAAAAAC 4480
GACGCCCGAAGAAGCCAGTCGCCTGAACGGGTGGCTTCCC 4520
AGGAAATTTGCAAAGACGGGCGACTCTGGACTTCCCTCTC 4560
CATCAGTCGGCAAGAGATTCAACGCCGTTGTCATGGGACG 4600
        4610      4620      4630      4640
GAAAAACTGGGAAAGCATGCCTCGAAAGTTTAGACCCCTC 4640
GTGGACAGATTGAACATCGTCGTTTCCTCTTCCCTCAAAG 4680
AAGAAGACATTGCGGCGGAGAAGCCTCAAGCTGAAGGCCA 4720
GCAGCGCGTCCGAGTCTGTGCTTCACTCCCAGCAGCTCTC 4760
AGCCTTCTGGAGGAAGAGTACAAGGATTCTGTCGACCAGA 4800
        4810      4820      4830      4840
TTTTTGTCGTGGGAGGAGCGGGACTGTACGAGGCAGCGCT 4840
GTCTCTGGGCGTTGCCTCTCACCTGTACATCACGCGTGTA 4880
GCCCGCGAGTTTCCGTGCGACGTTTTCTTCCCTGCGTTCC 4920
CCGGAGATGACATTCTTTCAAACAAATCAACTGCTGCGCA 4960
GGCTGCAGCTCCTGCCGAGTCTGTGTTCGTTCCCTTTTGT 5000
        5010      5020      5030      5040
CCGGAGCTCGGAAGAGAGAAGGACAATGAAGCGACGTATC 5040
GACCCATCTTCATTTCCAAGACCTTCTCAGACAACGGGGT 5080
ACCCTACGACTCCGTGGTTCTCGAGAAGAGAAGGAAGACT 5120
GACGACGCAGCCACTGCGGAACCGAGCAACGCAATGAGCT 5160
CCTTGACGTCCACGAGGGAGACAACTCCCGTGCACGGGTT 5200
        5210      5220      5230      5240
GCAGGCTCCTTCTTCGGCCGCAGCCATTGCCCCGGTGTTG 5240
GCGTGGATGGACGAAGAAGACCGGAAAAAACGCGAGCAAA 5280
AGGAACTGATTCGGGCCGTTCCGCATGTTCACTTTAGAGG 5320
CCATGAAGAATTCCAGTACCTTGATCTCATTGCCGACATT 5360
ATTAACAATGGAAGGACAATGGATGACCGAACGGGCGTTG 5400
        5410      5420      5430      5440
GTGTCATCTCCAAATTCGGCTGCACTATGCGCTACTCGCT 5440
GGATCAGGCCTTTCCACTTCTCACCACAAAGCGTGTGTTC 5480
TGGAAAGGGGTCCTCGAAGAGTTGCTGTGGTTCATTCGCG 5520
GCGACACGAACGCAAACCATCTTTCTGAGAAGGGCGTGAA 5560
GATCTGGGACAAGAATGTGACACGCGAGTTCCTCGATTCG 5600
```

```
        5610      5620      5630      5640
CGCAATCTCCCCCACCGAGAGGTCGGAGACATCGGCCCGG 5640
GCTACGGCTTCCAGTGGAGACACTTCGGCGCGGCATACAA 5680
AGACATGCACACAGACTACACAGGGCAGGGCGTCGACCAG 5720
CTGAAGAATGTGATCCAGATGCTGAGAACGAATCCAACAG 5760
ATCGTCGCATGCTCATGACTGCCTGGAATCCTGCAGCGCT 5800
        5810      5820      5830      5840
GGACGAAATGGCGCTGCCGCCTTGTCACTTGTTGTGCCAG 5840
TTCTACGTGAACGACCAGAAGGAGCTGTCGTGCATCATGT 5880
ATCAGCGGTCGTGCGATGTCGGCCTCGGCGTCCCCTTCAA 5920
CATCGCTTCCTATTCGCTTTTGACGCTCATGGTTGCACAC 5960
GTCTGCAACCTAAAACCTAAGGAGTTCATTCACTTCATGG 6000
        6010      6020      6030      6040
GGAACACGCATGTCTACACGAACCATGTCGAGGCTTTAAA 6040
AGAGCAGCTGCGGAGAGAACCGAGACCGTTCCCCATTGTG 6080
AACATCCTCAACAAGGAACGCATCAAGGAAATCGACGATT 6120
TCACCGCCGAGGATTTTGAGGTCGTGGGCTACGTCCCGCA 6160
CGGACGAATCCAGATGGAGATGGCTGTCTAGATAACTTCG 6200
        6210      6220      6230      6240
TATAGCATACATTATACGAAGTTATGGTACCAGCGTAAGC 6240
GAGAAACGGCACACAAGGCGGCCGTTTCAGTTCTCCCGGC 6280
GTTCGCAACGAAACCCCCCAAACGGGGAAAACGGCGACAC 6320
CCAGGAGGCAGAACGGCTATACGCGACGGGGGAGAAGGCG 6360
ATCCACGGAAGAGCACACCCGTCGCCGAATGCAGACTGCA 6400
        6410      6420      6430      6440
AAGAGCTGTACAAACGAACCCGGCGCCACAAAAACGAGCG 6440
TGTAGATAAACGAAAAAAAGCAGAATCGCGAAGTTGGCTG 6480
GCGCTCCAACTGGCACGAGAAATTTAGGTGTACATACATA 6520
TGGTCCGATGTACAGACACCGGAGGAACTCCGAGTGTCTC 6560
TCGGTGTAAATTCCGCGTCTTGGTTTCTCCTCGACTCAGA 6600
        6610      6620      6630      6640
GGGGAAATAGTGTACACTGACTTTTTCTTTCACATCGAGG 6640
CTCAGCGCGCAGAACGTCTCTTTTTTCCGTTTGAAGACAC 6680
AGTCGTCGGAAGTCTCTTCTTTTGGTGTAGCGATACTGCT 6720
TTCGCTGGTGCTGGCGAAGCGCCGAGCCGGGTTCCATCAA 6760
CAGCCGTTTAAAACAGATTTTTGAACTAAGAGACACAGAG 6800
        6810      6820      6830      6840
CCACTGCATTTTGACGATCAAGCTGCATTACATGTAGACT 6840
TTACACTGGCTGTTTCAGTTTCCAGCGAAGCTTCCTTCGT 6880
CTTTTTATTTGCATACTTGTTTTCGCAACGCGATGCCGCT 6920
CAGCAGCAGGCAGAGCCTCTCTCCTGGAGGCGCAGTGTTT 6960
TCTTTTTTCGTACACTGGATCGACCAGCCGTTGCGGCCGC 7000
```

```
        7010      7020      7030      7040
GAGTCTCTTTCGGAGCAGTTCCGCTTCTTTGCCCGTACGG 7040
GGTCTCTGTGGGGGCAGACTCTTAGGCGAAAGCAGCGAGT 7080
GCGGCGACTCTGTGTAGTCGGGAGACGCGTGGAAAAAGCG 7120
CGGCGCGAAATGGATTTCTGGATGCGGAGAAAGAGTTGAG 7160
GTTGTCGATCTTTCGCAACTGGAGTGATTCTGGCCTGCCT 7200
        7210      7220      7230      7240
GTTCTCACAAAAAACAAATGAGCATCTATGCCGGCTTTTG 7240
CGACTTGGAGAAGTGTACTGACATCTGTTCGAGGTGTTGG 7280
AGCGTAAACTGTTCCTTCTGATCTTTCGGTGGAGTCTTTC 7320
AAGCTCTCAGTTTTCTCGCTCTTTGTCGACCTTTGCGGAA 7360
GCGTTTGCATTTAAATGGATCCTCTAGAGTCGACCTGCAG 7400
        7410      7420      7430      7440
GCATGCAAGCTTGGCGTAATCATGGTCATAGCTGTTTCCT 7440
GTGTGAAATTGTTATCCGCTCACAATTCCACACAACATAC 7480
GAGCCGGAAGCATAAAGTGTAAAGCCTGGGGTGCCTAATG 7520
AGTGAGCTAACTCACATTAATTGCGTTGCGCTCACTGCCC 7560
GCTTTCCAGTCGGGAAACCTGTCGTGCCAGCTGCATTAAT 7600
        7610      7620      7630      7640
GAATCGGCCAACGCGCGGGGAGAGGCGGTTTGCGTATTGG 7640
GCGCTCTTCCGCTTCCTCGCTCACTGACTCGCTGCGCTCG 7680
GTCGTTCGGCTGCGGCGAGCGGTATCAGCTCACTCAAAGG 7720
CGGTAATACGGTTATCCACAGAATCAGGGGATAACGCAGG 7760
AAAGAACATGTGAGCAAAAGGCCAGCAAAAGGCCAGGAAC 7800
        7810      7820      7830      7840
CGTAAAAAGGCCGCGTTGCTGGCGTTTTTCCATAGGCTCC 7840
GCCCCCCTGACGAGCATCACAAAAATCGACGCTCAAGTCA 7880
GAGGTGGCGAAACCCGACAGGACTATAAAGATACCAGGCG 7920
TTTCCCCCTGGAAGCTCCCTCGTGCGCTCTCCTGTTCCGA 7960
CCCTGCCGCTTACCGGATACCTGTCCGCCTTTCTCCCTTC 8000
        8010      8020      8030      8040
GGGAAGCGTGGCGCTTTCTCATAGCTCACGCTGTAGGTAT 8040
CTCAGTTCGGTGTAGGTCGTTCGCTCCAAGCTGGGCTGTG 8080
TGCACGAACCCCCCGTTCAGCCCGACCGCTGCGCCTTATC 8120
CGGTAACTATCGTCTTGAGTCCAACCCGGTAAGACACGAC 8160
TTATCGCCACTGGCAGCAGCCACTGGTAACAGGATTAGCA 8200
        8210      8220      8230      8240
GAGCGAGGTATGTAGGCGGTGCTACAGAGTTCTTGAAGTG 8240
GTGGCCTAACTACGGCTACACTAGAAGAACAGTATTTGGT 8280
ATCTGCGCTCTGCTGAAGCCAGTTACCTTCGGAAAAAGAG 8320
TTGGTAGCTCTTGATCCGGCAAACAAACCACCGCTGGTAG 8360
CGGTGGTTTTTTTGTTTGCAAGCAGCAGATTACGCGCAGA 8400
```

```
        8410      8420      8430      8440
AAAAAAGGATCTCAAGAAGATCCTTTGATCTTTTCTACGG 8440
GGTCTGACGCTCAGTGGAACGAAAACTCACGTTAAGGGAT 8480
TTTGGTCATGAGATTATCAAAAAGGATCTTCACCTAGATC 8520
CTTTTAAATTAAAAATGAAGTTTTAAATCAATCTAAAGTA 8560
TATATGAGTAAACTTGGTCTGACAGTTACCAATGCTTAAT 8600
        8610      8620      8630      8640
CAGTGAGGCACCTATCTCAGCGATCTGTCTATTTCGTTCA 8640
TCCATAGTTGCCTGACTCCCCGTCGTGTAGATAACTACGA 8680
TACGGGAGGGCTTACCATCTGGCCCCAGTGCTGCAATGAT 8720
ACCGCGAGACCCACGCTCACCGGCTCCAGATTTATCAGCA 8760
ATAAACCAGCCAGCCGGAAGGGCCGAGCGCAGAAGTGGTC 8800
        8810      8820      8830      8840
CTGCAACTTTATCCGCCTCCATCCAGTCTATTAATTGTTG 8840
CCGGGAAGCTAGAGTAAGTAGTTCGCCAGTTAATAGTTTG 8880
CGCAACGTTGTTGCCATTGCTACAGGCATCGTGGTGTCAC 8920
GCTCGTCGTTTGGTATGGCTTCATTCAGCTCCGGTTCCCA 8960
ACGATCAAGGCGAGTTACATGATCCCCCATGTTGTGCAAA 9000
        9010      9020      9030      9040
AAAGCGGTTAGCTCCTTCGGTCCTCCGATCGTTGTCAGAA 9040
GTAAGTTGGCCGCAGTGTTATCACTCATGGTTATGGCAGC 9080
ACTGCATAATTCTCTTACTGTCATGCCATCCGTAAGATGC 9120
TTTTCTGTGACTGGTGAGTACTCAACCAAGTCATTCTGAG 9160
AATAGTGTATGCGGCGACCGAGTTGCTCTTGCCCGGCGTC 9200
        9210      9220      9230      9240
AATACGGGATAATACCGCGCCACATAGCAGAACTTTAAAA 9240
GTGCTCATCATTGGAAAACGTTCTTCGGGGCGAAAACTCT 9280
CAAGGATCTTACCGCTGTTGAGATCCAGTTCGATGTAACC 9320
CACTCGTGCACCCAACTGATCTTCAGCATCTTTTACTTTC 9360
ACCAGCGTTTCTGGGTGAGCAAAAACAGGAAGGCAAAATG 9400
        9410      9420      9430      9440
CCGCAAAAAAGGGAATAAGGGCGACACGGAAATGTTGAAT 9440
ACTCATACTCTTCCTTTTTCAATATTATTGAAGCATTTAT 9480
CAGGGTTATTGTCTCATGAGCGGATACATATTTGAATGTA 9520
TTTAGAAAAATAAACAAATAGGGGTTCCGCGCACATTTCC 9560
CCGAAAAGTGCCACCTGACGTCTAAGAAACCATTATTATC 9600
        9610      9620      9630      9640
ATGACATTAACCTATAAAAATAGGCGTATCACGAGGCCCT 9640
TTCGTC 9646
```

```
LOCUS       pT3Comp               11949 bp
        23-JUL-2014
EMBL_ID     pT3Comp               11949 bp
        23-APR-2014
EMBL_ID     pT3Comp               11949 bp
        22-APR-2014
EMBL_ID     pT3Comp               11949 bp
        12-MAY-2014
UNIMARK     pT3Comp annotations
            pT3Comp



ORIGIN
Features
     rep_origin         1..217
     /note="ori2; oriS"
     /label="ori2; oriS rep origin"
     misc_feature       1..1804
     /label="pSMART-BAC2-5'"
     source             1..11949
     /comment="Molecule is created with Unipro UGENE v1.13"
     rep_origin         293..907
     /note="oriV"
     /label="oriV rep origin"
     primer_bind        1765..1784
     /label="pSMART-seqF"
     primer_bind        complement(1787..1812)
     /label="pBAC2-SwaI-R"
     primer_bind        1793..1832
     /label="T3-5p-RecombF"
     primer_bind        1804..1824
     /label="T3-UPRTf"
     /ugene_group="misc_recomb"
     misc_feature       1805..1812
     /label="SwaI"
     misc_recomb        1813..2858
     /created_by="User"
     /label="5'Recomb"
     primer_bind        1813..1833
     /created_by="User"
     /label="T3ko-5'F"
     misc_feature       complement(2838..2858)
     /created_by="User"
     /label="T3ko-5'R"
     /note="Geneious type: primer_bind_reverse"
     primer_bind        2945..2964
     /label="T3CompSeqF"
     primer_bind        complement(2966..2991)
     /mismatches=0
     primer_bind        2977..3009
     /label="T3CDRcompF"
```

```
/label="T3ko-5'R"
/note="Geneious type: primer_bind_reverse"
```

```
primer_bind         2945..2964
/label="T3CompSeqF"
```

```
primer_bind         complement(2966..2991)
/mismatches=0
```

```
primer_bind         2977..3009
/label="T3CDRcompF"
```

```
primer_bind         2977..2991
/mismatches=0
```

```
misc_feature        2988..2990
/label="Start"
```

```
CDS                 2991..4892
/label="T3_dcs (concatenated sequence 2)"
/note="Geneious type: Concatenated sequence"
```

```
primer              2991..3007
/note="Forward primer region"
/label="F1"
```

```
Region              3048..3116
/label="TransMembrane"
```

```
primer              3219..3245
/label="primer from paper"
```

```
Region              3420..4454
/label="Transferase"
```

```
Region              4476..4829
/label="Ricin-B-Lectin"
```

```
primer_bind         4477..4498
/created_by="User"
/label="T3colonyF"
```

```
primer              complement(4525..4548)
/label="primer from paper"
```

```
misc_feature        complement(4818..4839)
/created_by="User"
/label="T3colonyR2"
/note="Geneious type: primer_bind_reverse"
```

```
primer_bind         complement(4874..4926)
/label="T3CDRcompR"
```

```
primer              complement(4876..4892)
/note="Reverse primer region"
/label="R1"
```

```
misc_feature        4893..4898
/label="GAlinker"
```

```
misc_feature        4899..4922
/mismatches=0
/label="FLAG"
/ugene_name="Tag"
primer_bind         4910..4949
/label="T3-3p-RecombF"
primer_bind         4910..4926
/mismatches=0
misc_feature        4923..4925
/label="Stop"
misc_feature        4926..5440
/Source="annotation"
/isDeprecated=0
/label="UTR"
/ugene_name="UTR"
misc_recomb         4987..6167
/created_by="User"
/label="3'Recomb"
primer_bind         4987..5007
/created_by="User"
/modified_by="User"
/label="T3ko-3'F"
misc_feature        complement(5289..5308)
/created_by="User"
/label="T3detectR"
/note="Geneious type: primer_bind_reverse"
primer_bind         complement(5449..5466)
/label="T3-UPRTr"
misc_feature        complement(6147..6167)
/created_by="User"
/label="T3ko-3'R"
/note="Geneious type: primer_bind_reverse"
primer_bind         complement(6148..6183)
/label="T3-3p-RecombR"
misc_feature        6168..11949
/label="pSMAR-BAC2-3'"
primer_bind         6168..6186
/label="pBAC2-F"
misc_feature        6168..6183
/ugene_group="primer_bind"
CDS                 6390..7049
/gene="CAT"
```

```
/EC_number="2.3.1.28"
/codon_start=1
/transl_table=11
/product="chloramphenicol acetyltransferase"
/protein_id="ABW82143.1"
/db_xref="GI:158854232"
/translation="MEKKITGYTTVDISQWHRKEHFEAFQSVAQCTYNQTVQLDITAFLKTVKKNKHKFYPAFIHILA
RLMNAHPEFRMAMKDGELVIWDSVHPCYTVFHEQTETFSSLWSEYHDDFRQFLHIYSQDVACYGENLAYFPKGFIENM
FFVSANPWVSFTSFDLNVANMDNFFAPVFTMGKYYTQGDKVLMPLAIQVHHAVCDGFHVGRMLNELQQYCDEWQGGA
"
/label="CAT CDS"
```

```
gene                    6390..7049
/gene="CAT"
/label="CAT gene"
```

```
misc_feature            7120..7153
/note="loxP site"
/label="loxP site"
```

```
misc_feature            7167..7569
/note="cosN"
/label="cosN"
```

```
CDS                     complement(8376..9347)
/gene="sopB"
/note="partitioning protein; essential for partitioning
ofF' plasmid replicon (RepFIA)"
/codon_start=1
/transl_table=11
/product="SopB"
/protein_id="ABW82144.1"
/db_xref="GI:158854233"
/translation="MKRAPVIPKHTLNTQPVEDTSLSTPAAPMVDSLIARVGVMARGNAITLPVCGRDVKFTLEVLRG
DSVEKTSRVWSGNERDQELLTEDALDDLIPSFLLTGQQTPAFGRRVSGVIEIADGSRRRKAAALTESDYRVLVGELDD
EQMAALSRLGNDYRPTSAYERGQRYASRLQNEFAGNISALADAENISRKIITRCINTAKLPKSVVALFSHPGELSAR
SGDALQKAFTDKEELLKQQASNLHEQKKAGVIFEAEEVITLLTSVLKTSSASRTSLSSRHQFAPGATVLYKGDKMVLN
LDRSRVPTECIEKIEAILKELEKPAP"
/label="sopB CDS"
```

```
gene                    complement(8376..9347)
/gene="sopB"
/label="sopB gene"
```

```
CDS                     complement(9347..10522)
/gene="sopA"
/note="partitioning protein; essential for partitioning
ofF' plasmid replicon (RepFIA)"
```

/codon_start=1
/transl_table=11
/product="SopA"
/protein_id="ABW82145.1"
/db_xref="GI:158854234"
/translation="MFRMKLMETLNQCINAGHEMTKAIAIAQFNDDSPEARKITRRWRIGEAADLVGVSSQAIRDAEK
AGRLPHPDMEIRGRVEQRVGYTIEQINHMRDVFGTRLRRAEDVFPPVIGVAAHKGGVYKTSVSVHLAQDLALKGLRVL
LVEGNDPQGTASMYHGWVPDLHIHAEDTLLPFYLGEKDDVTYAIKPTCWPGLDIIPSCLALHRIETELMGKFDEGKL
PTDPHLMLRLAIETVAHDYDVIVIDSAPNLGIGTINVVCAADVLIVPTPAELFDYTSALQFFDMLRDLLKNVDLKGFE
PDVRILLTKYSNSNGSQSPWMEEQIRDAWGSMVLKNVVRETDEVGKGQIRMRTVFEQAIDQRSSTGAWRNALSIWEPV
CNEIFDRLIKPRWEIR"
/label="sopA CDS"

---

gene complement(9347..10522)
/gene="sopA"
/label="sopA gene"

CDS complement(11101..11856)
/gene="repE"
/note="replication initiation protein"
/codon_start=1
/transl_table=11
/product="RepE"
/protein_id="ABW82146.1"
/db_xref="GI:158854235"
/translation="MAETAVINHKKRKNSPRIVQSNDLTEAAYSLSRDQKRMLYLFVDQIRKSDGTLQEHDGICEIHV
AKYAEIFGLTSAEASKDIRQALKSFAGKEVVFYRPEEDAGDEKGYESFPWFIKRAHSPSRGLYSVHINPYLIPFFIGL
QNRFTQFRLSETKEITNPYAMRLYESLCQYRKPDGSGIVSLKIDWIIERYQLPQSYQRMPDFRRRFLQVCVNEINSR
TPMRLSYIEKKKGRQTTHIVFSFRDITSMTTG"
/label="repE CDS"

---

gene complement(11101..11856)
/gene="repE"
/label="repE gene"

```
        10        20        30        40
CTTCTGAGGGCAATTTGTCACAGGGTTAAGGGCAATTTGT 40
CACAGACAGGACTGTCATTTGAGGGTGATTTGTCACACTG 80
AAAGGGCAATTTGTCACAACACCTTCTCTAGAACCAGCAT 120
GGATAAAGGCCTACAAGGCGCTCTAAAAAAGAAGATCTAA 160
AAACTATAAAAAAAATAATTATAAAAATATCCCCGTGGAT 200
       210       220       230       240
AAGTGGATAACCCCAAGGGAAGTTTTTTCAGGCATCGTGT 240
GTAAGCAGAATATATAAGTGCTGTTCCCTGGTGCTTCCTC 280
GCTCACTCGACCGGGAGGGTTCGAGAAGGGGGGGCACCCC 320
CCTTCGGCGTGCGCGGTCACGCGCACAGGGCGCAGCCCTG 360
GTTAAAAACAAGGTTTATAAATATTGGTTTAAAAGCAGGT 400
```

```
        410       420       430       440

TAAAAGACAGGTTAGCGGTGGCCGAAAAACGGGCGGAAAC 440
CCTTGCAAATGCTGGATTTTCTGCCTGTGGACAGCCCCTC 480
AAATGTCAATAGGTGCGCCCCTCATCTGTCAGCACTCTGC 520
CCCTCAAGTGTCAAGGATCGCGCCCCTCATCTGTCAGTAG 560
TCGCGCCCCTCAAGTGTCAATACCGCAGGGCACTTATCCC 600

        610       620       630       640

CAGGCTTGTCCACATCATCTGTGGGAAACTCGCGTAAAAT 640
CAGGCGTTTTCGCCGATTTGCGAGGCTGGCCAGCTCCACG 680
TCGCCGGCCGAAATCGAGCCTGCCCCTCATCTGTCAACGC 720
CGCGCCGGGTGAGTCGGCCCCTCAAGTGTCAACGTCCGCC 760
CCTCATCTGTCAGTGAGGGCCAAGTTTTCCGCGAGGTATC 800

        810       820       830       840

CACAACGCCGGCGGCCGGCCGCGGTGTCTCGCACACGGCT 840
TCGACGGCGTTTCTGGCGCGTTTGCAGGGCCATAGACGGC 880
CGCCAGCCCAGCGGCGAGGGCAACCAGCCGAGGGCTTCGC 920
CCTGTCGCTCGACTGCGGCGAGCACTACTGGCTGTAAAAG 960
GACAGACCACATCATGGTTCTGTGTTCATTAGGTTGTTCT 1000

       1010      1020      1030      1040

GTCCATTGCTGACATAATCCGCTCCACTTCAACGTAACAC 1040
CGCACGAAGATTTCTATTGTTCCTGAAGGCATATTCAAAT 1080
CGTTTTCGTTACCGCTTGCAGGCATCATGACAGAACACTA 1120
CTTCCTATAAACGCTACACAGGCTCCTGAGATTAATAATG 1160
CGGATCTCTACGATAATGGGAGATTTTCCCGACTGTTTCG 1200

       1210      1220      1230      1240

TTCGCTTCTCAGTGGATAACAGCCAGCTTCTCTGTTTAAC 1240
AGACAAAAACAGCATATCCACTCAGTTCCACATTTCCATA 1280
TAAAGGCCAAGGCATTTATTCTCAGGATAATTGTTTCAGC 1320
ATCGCAACCGCATCAGACTCCGGCATCGCAAACTGCACCC 1360
GGTGCCGGGCAGCCACATCCAGCGCAAAAACCTTCGTGTA 1400

       1410      1420      1430      1440

GACTTCCGTTGAACTGATGGACTTATGTCCCATCAGGCTT 1440
TGCAGAACTTTCAGCGGTATACCGGCATACAGCATGTGCA 1480
TCGCATAGGAATGGCGGAACGTATGTGGTGTGACCGGAAC 1520
AGAGAACGTCACACCGTCAGCAGCAGCGGCGGCAACCGCC 1560
TCCCCAATCCAGGTCCTGACCGTTCTGTCCGTCACTTCCC 1600

       1610      1620      1630      1640

AGATCCGCGCTTTCTCTGTCCTTCCTGTGCGACGGTTACG 1640
CCGCTCCATGAGCTTATCGGGTCCACTCGCGAACGCAGAA 1680
AGGCCCACCCGAAGGTGAGCCAGTGTGATTACATTTGCGG 1720
CCTAACTGTGGCCAGTCCAGTTACGCTGGAGTCACTAGTA 1760
TTTAGGTGACACTATAGAAGCGGCCGCGACAACTTGTCCA 1800
```

```
        1810      1820      1830      1840
CGTGATTTAAATAAAGATGCGCCGCCGATCAAACAGCGAG 1840
AACTTTAAGACCTCAAGCGCGCCTTAGGGCGCGAACAGCA 1880
GAGAGAAAAGCTGCGTGCTAGAGGAAACGAAACCCAGTCA 1920
ACATTTGTAAAACCGCCATGTTTTCCGGGGTTATTTATTT 1960
TTATACGAGAACCCGCAGGACACAGCTGGAGAGCTGTTGG 2000
        2010      2020      2030      2040
GGAAAAAAGCCTTTGTGTGTGCGAGTGCATGTGCAAGGGA 2040
GGTAAACTGGCTCCGCCGCTCCCCTGCGCTGGCAATTTGC 2080
CATTCTTACAGCTCTCGTTTCCTTCCCCTTCCTGTCGCTA 2120
TACGCTTTTCGTTTCAGGGGTGCTTTGTTTCTGCGAGACC 2160
AGTGCCTTGCCCCTTCTCGTATGTCACCCCGTCTGTGTTC 2200
        2210      2220      2230      2240
CGGGATGCTGCTGCGTGCACCGAGTCTGGCTCGCTTCGAG 2240
GAAAACGAACGAGTGTGAGTTTGACTCACTTCTGCTCGAA 2280
AAGTCAAATGCGTGTTTTTCTCCACTGGTAGGACAAATGT 2320
ACTTATTTTGGCCATCAACAGACGCCTGAGCCCACACTGC 2360
TGCACATCCGCCCCTGTTAGACAGGTCGGCATTGCTTTGC 2400
        2410      2420      2430      2440
TCGTGTGTGCGCGGCATCTGCGTGAGACACAACTATCCAG 2440
TGTGCACATGCTTGTTAGTAACTATGCGAAAGAGCCACAA 2480
AATCCGCTGATCATACATCTATGCATGCTTGATCATGCGT 2520
GATTCAGTGTCAGCAAGTGCGAGAAACTGCCGGTTTGCCG 2560
GCAAGAGTCGCTTTTTCTAGCTGCATCACCAAGAGACCAA 2600
        2610      2620      2630      2640
CTAGAGGAAGTACATCGTGTTCGTCGTCCCGACATCTCCA 2640
TTTCCTGTGTGTCTGCGTGCTTTGCTTTTCTCTCAACCTT 2680
CCGTTTTTCGTTTCTGTTCGCGAAATGTCGATCGAACAGT 2720
GCAATTCTCTGTTGTCCCTCACTGCGTTTGCCAATGCCAT 2760
AAGACAGTTACGTCCTGTCACACAAATCGCCATCATTCCC 2800
        2810      2820      2830      2840
CCTGTCTCTTCCACGTTTCTGCACAAGCACCTCACGTGGT 2840
CGGCGGTGCTCATTTGAATCCTTCGTTTCTGTGTTCTCCA 2880
CGTTCCGTCGCTTCCCCCCCTTCCCGTACAAGCACTGCTT 2920
CGGTTGTTTTCGGCAAGTTCCTTCGCGTCTGGTTTCGGTA 2960
CTCAAAACGAATCAGGAGCGGCTGAGAATGAGGGTTATGA 3000
        3010      3020      3030      3040
CGCCTCCGTCGGCGCCTCACCCGCGCGCATCGCCGTGGTG 3040
GGCGAGTTGCCTCTGGGCGGCCCTCCAGATCATTCTTTTT 3080
TTGGTCGGCATATGCTGGGTGGCAGTGAATGTCGGGTTTC 3120
TCTCTGATTATTACTTCAAAGGTTCAGATTTCTCGTCACC 3160
ATCGCTCAACAACAACAGCGATGCTTCTCGCCAACCGCCG 3200
```

```
        3210      3220      3230      3240

CGCGCCGCCAGAGATGTGCAGGAAGGCGTGTCGTCGTTTG 3240
AAGAATCCGGAGGGGCGCAGCTCCCGCCGCGTGTGCACGC 3280
ACTTGAGGTTCTTGAGGGAGCTGGCCCCGGCCGACTTCAC 3320
GGGAGGCTCGGAATCAAACCTGATGGCCAGCCGGGCTACA 3360
CACGGGCACCCTCGCCTCCTACAGATCTGAGCATGCCCCA 3400

        3410      3420      3430      3440

AGCACTGGCTCGCGGGGGAGGCTTCAATCTGTATCTGTCT 3440
GACCATCTGGAGCTGGACCGCACGGCGCCTGACGCGCGGC 3480
ACGCGTCCTGTCGGCAGCTACACTACGACCTCTCGACGCT 3520
GCCGAAGGCATCAGTGATTATCGTGTTTTACAACGAGCCA 3560
TTTTCGACGTTGATGCGGTCCGTCCACAGCGTTCTGAACG 3600

        3610      3620      3630      3640

GGACTCCTCCGCAGATTCTGGAGGAGCTGATTCTCGTCGA 3640
CGATGGCAGCACGTTGCCGTACATCCGGGAAGACGGCAAC 3680
CAACAGTTGGTGGAGTACCTTAAGCTGCTTCCGGCGAAGG 3720
TGCGGCTCATCCGCAACGAGGTGCGCAAAGGGATTGTCGG 3760
TGCACGGATGAAGGGGATTCGCGCGAGTCGCGCGCCGATC 3800

        3810      3820      3830      3840

TTCGCTATCCTCGACAGCCACATCGAGGTGAGTCCTCAGT 3840
GGCTGGAGCCGCTGCTGCTGCGGATCAAGGAAGACGGCCG 3880
GCGCGTGGTGATGCCGCAGATCGATGGAATCGACGCGGAA 3920
ACCTTCAAGCACATCGCCGGCGGGATAGGCTGCAAGCTGG 3960
GGTTTCTCTGGAAGCTGATGGAGCACTCGTACGAGGGCCA 4000

        4010      4020      4030      4040

CCAGACCGCGCGACTGCCGCCGGAGGAACGCCAACCGTCT 4040
CCGACCGACTTTCAGACGTCGCCGGCGATGGCCGGGGGTC 4080
TTTTTGCAGCGAACAAGGCGTTCTTCTTTGACGTCGGAGC 4120
CTACGATGAAGACTTCCAGTTCTGGGGAACAGAAAACTTA 4160
GAACTGAGTTTCCGCCTCTGGCAGTGCGGCGGCGTCCTGG 4200

        4210      4220      4230      4240

AGTGCGCTCCTTGCTCTCGCGTCTACCACATCTTTCGAAA 4240
AGGCGGCTCCGGCTACAGCAGTCCAGGCGACTCCATCACC 4280
ATCAACAAGATGCGGACGATGCTGTGGATGGACGAGTATG 4320
CCGACCTCGCCTGGCGAGTCATTGGGAAACCCAGAGTCAA 4360
CTACCGGCCCGAGAGCCTTGAAAAACGCAGAGAGTGGAGA 4400

        4410      4420      4430      4440

AAGAGGAAGGGATGTAAAAGCTTCCGCTGGTTCATGGAAA 4440
ACGTCTTTCCCGAGGGAGATGTCGTCACTCTCGACGACGT 4480
CCCGTACCTTGGCCCTTTGAGAAATGACAAGATAGGCATG 4520
TGTTTGGACAACATGGGCTGGGCCTCTCCCGGACATGCTG 4560
TTGGCCTCGAGTATTGCCACGGGGGCGACACTCAGACATT 4600
```

```
        4610      4620      4630      4640
....|....|....|....|....|....|....|....|

TATGTTTTTAGGAAGGTAGGACACGTAATGCCAGTTAAT 4640
GATGACGAGGCTTGCTTGCAGCCGTCAGGAAGACTGGACT 4680
GGTGTCGAGGAACGGCCCAATTCTGGTGGGACTTCACATC 4720
GAGTGGCCAACTGATGTTTCGCGAAACAAAGCAGTGCCTC 4760
TCAGCCTTTGGCAGAAAACTCAGGATGGTCGAGTGCGATG 4800

        4810      4820      4830      4840
....|....|....|....|....|....|....|....|

ACACTGATCCTTACCAGATCTGGTCGTGGACGGCCTACAA 4840
TCCACCCGATACATTCACGTTTCCGTCGGTTAGCAGATCA 4880
ATCCGAAGTGGCGGAGCTGATTACAAGGATGACGACGATA 4920
AGTAGGAAAGTAAAGGAAAAGACGATTCAGGAACTGCTTC 4960
TCAGTCGCCGACGGTACTGGGAAGGCTGCTGCGGCTGCTG 5000

        5010      5020      5030      5040
....|....|....|....|....|....|....|....|

TAAAACTGGTTGTTGCAGCTGTTGCAGCTGGATCCGAGGA 5040
TGTTGGAGATCATAAATGCTCCGTTTAAGTAATGGGTGGT 5080
TGGCCCAAGTGCCACAGAGAAAAATGCGATTTGTAGAACC 5120
GTAACGAAGCGACTGGTTCACCTAATTCCTGCGTGGCAAG 5160
TGACTCCCGTGCGGGTGTCTCAACGGGAGAATGGTCTCAG 5200

        5210      5220      5230      5240
....|....|....|....|....|....|....|....|

ATTTCAATGTTGTGCTTTATATCATCTCGATTCACTGAGC 5240
GTCAGCTCTTCAATACATTAAGGCGGCCGACAGTTGTGCC 5280
CCAGACCCATGTATAGGCACTCGCGCAAAGGGTCCCGTGG 5320
TCATTCCTTCAGGAAAGCTTGTTGTGCTCCCCATAACTTG 5360
TCTTTTCAAGCTATTCGATTCTCTAGATTCCGTGTACGAA 5400

        5410      5420      5430      5440
....|....|....|....|....|....|....|....|

CCCCTGAACCGGGTCAACTCATCCGCTTGATTCGCATCGT 5440
CTACACAGAACCCAATGAACTACACCAATGCACGCAGTCT 5480
AGAAAACTGTCCAATACCTTCAGAAGGTGGTGAACTCGTG 5520
CTCGAGATACGTAACTTCCCCATGCATTAGTCTGGTTACT 5560
GGGAAGAGATTCGTTAGTTCGGCGCTACGTTGTAGTCAGA 5600

        5610      5620      5630      5640
....|....|....|....|....|....|....|....|

ATTCTTGGTTGTCTGATGAAACACAAGAGCGCTCTTGACC 5640
CGCGAAGTACTGCGGGTGAGCATGTAGACGCCCAGCACGA 5680
GTGTTTCATTCGAACAGGGAATTCCCACGTAGCTCCGCAT 5720
GACCTGACAACTTCGGGTGTGTGTGCATTCTGTATACTGA 5760
TTGAGCTCACTTGGTAAAACCTTGATTCTGGAATGTCCGT 5800

        5810      5820      5830      5840
....|....|....|....|....|....|....|....|

GGTACACAGCAATGTCCTCGTGGACGCGAGCGAGTTATTC 5840
GCCAGTTCCTTTCTTGTAATTGTGGACCATCTGCAGGTCT 5880
CGAAGGAAGATTATCACTTTTTAAACGGCATGTGGCGTTT 5920
TCGTTAGGTCACATTTAGGTTTCCACTCCCCCGGTCAGTT 5960
GCCAACAGCAACGGAAAGCACAAAGGAGGAAGCTGGTGAG 6000
```

```
        6010      6020      6030      6040
GGACTTCACGGGCTTTTACACAGCGCACTATGGTGCCCAG 6040
TCATGGTATACGCTTGCATCCAGTGTTCGAGCACACAAAG 6080
GCGAATGTACACCCTTTGACAACCGAAGCGACTCCGGCGA 6120
TGCCTACTCTCTAATGGATTGAAAATTCGTGCATGCGTCC 6160
ACTCTTTACGTGACTTGAAGTCGCGGCCGCACTGACCCTA 6200
        6210      6220      6230      6240
TAGTGAGTCGTATTAATTTAAATCATACCAACATGGTCAA 6240
ATAAAACGAAAGGCTCAGTCGAAAGACTGGGCCTTTCGTT 6280
TTAATCTGATCGGCACGTAAGAGGTTCCAACTTTCACCAT 6320
AATGAAATAAGATCACTACCGGGCGTATTTTTTGAGTTAT 6360
CGAGATTTTCAGGAGCTAAGGAAGCTAAAATGGAGAAAAA 6400
        6410      6420      6430      6440
AATCACTGGATATACCACCGTTGATATATCCCAATGGCAT 6440
CGTAAAGAACATTTTGAGGCATTTCAGTCAGTTGCTCAAT 6480
GTACCTATAACCAGACCGTTCAGCTGGATATTACGGCCTT 6520
TTTAAAGACCGTAAAGAAAAATAAGCACAAGTTTTATCCG 6560
GCCTTTATTCACATTCTTGCCCGCCTGATGAATGCTCATC 6600
        6610      6620      6630      6640
CGGAATTTCGTATGGCAATGAAAGACGGTGAGCTGGTGAT 6640
ATGGGATAGTGTTCACCCTTGTTACACCGTTTTCCATGAG 6680
CAAACTGAAACGTTTTCATCGCTCTGGAGTGAATACCACG 6720
ACGATTTCCGGCAGTTTCTACACATATATTCGCAAGATGT 6760
GGCGTGTTACGGTGAAAACCTGGCCTATTTCCCTAAAGGG 6800
        6810      6820      6830      6840
TTTATTGAGAATATGTTTTTCGTCTCAGCCAATCCCTGGG 6840
TGAGTTTCACCAGTTTTGATTTAAACGTGGCCAATATGGA 6880
CAACTTCTTCGCCCCCGTTTTCACCATGGGCAAATATTAT 6920
ACGCAAGGCGACAAGGTGCTGATGCCGCTGGCGATTCAGG 6960
TTCATCATGCCGTTTGTGATGGCTTCCATGTCGGCAGAAT 7000
        7010      7020      7030      7040
GCTTAATGAATTACAACAGTACTGCGATGAGTGGCAGGGC 7040
GGGGCGTAACCTAGGTGACAGAAGTCAAAAGCCTCCGGTC 7080
GGAGGCTTTTGACTTTCTGCTAGATCGGCCGCATCGAATA 7120
TAACTTCGTATAATGTATGCTATACGAAGTTATTAGCGAT 7160
GAGCTCGGACTTCCATTGTTCATTCCACGGACAAAAACAG 7200
        7210      7220      7230      7240
AGAAAGGAAACGACAGAGGCCAAAAAGCTCGCTTTCAGCA 7240
CCTGTCGTTTCCTTTCTTTTCAGAGGGTATTTTAAATAAA 7280
AACATTAAGTTATGACGAAGAAGAACGGAAACGCCTTAAA 7320
CCGGAAAATTTTCATAAATAGCGAAAACCCGCGAGGTCGC 7360
CGCCCCGTAACCTGTCGGATCACCGGAAAGGACCCGTAAA 7400
```

```
        7410      7420      7430      7440

GTGATAATGATTATCATCTACATATCACAACGTGCGTGGA 7440
GGCCATCAAACCACGTCAAATAATCAATTATGACGCAGGT 7480
ATCGTATTAATTGATCTGCATCAACTTAACGTAAAAGCAA 7520
CTTCAGACAATACAAATCAGCGACACTGAATACGGGGCAA 7560
CCTCATGTCCGAGCTCGCGAGCTCGTCGACAGCGACACAC 7600

        7610      7620      7630      7640

TTGCATCGGATGCAGCCCGGTTAACGTGCCGGCACGGCCT 7640
GGGTAACCAGGTATTTTGTCCACATAACCGTGCGCAAAAT 7680
GTTGTGGATAAGCAGGACACAGCAGCAATCCACAGCAGGC 7720
ATACAACCGCACACCGAGGTTACTCCGTTCTACAGGTTAC 7760
GACGACCATGTCAATACTTGCCCTTGACAGGCATTGATGG 7800

        7810      7820      7830      7840

AATCGTAGTCTCACGCTGATAGTCTGATCGACAATACAAG 7840
TGGGACCGTGGTCCCAGACCGATAATCAGACCGACAACAC 7880
GAGTGGGATCGTGGTCCCAGACTAATAATCAGACCGACGA 7920
TACGAGTGGGACCGTGGTCCCAGACTAATAATCAGACCGA 7960
CGATACGAGTGGGACCGTGGTTCCAGACTAATAATCAGAC 8000

        8010      8020      8030      8040

CGACGATACGAGTGGGACCGTGGTCCCAGACTAATAATCA 8040
GACCGACGATACGAGTGGGACCATGGTCCCAGACTAATAA 8080
TCAGACCGACGATACGAGTGGGACCGTGGTCCCAGTCTGA 8120
TTATCAGACCGACGATACGAGTGGGACCGTGGTCCCAGAC 8160
TAATAATCAGACCGACGATACGAGTGGGACCGTGGTCCCA 8200

        8210      8220      8230      8240

GACTAATAATCAGACCGACGATACGAGTGGGACCGTGGTC 8240
CCAGTCTGATTATCAGACCGACGATACAAGTGGAACAGTG 8280
GGCCCAGAGAGAATATTCAGGCCAGTTATGCTTTCTGGCC 8320
TGTAACAAAGGACATTAAGTAAAGACAGATAAACGTAGAC 8360
TAAAACGTGGTCGCATCAGGGTGCTGGCTTTTCAAGTTCC 8400

        8410      8420      8430      8440

TTAAGAATGGCCTCAATTTTCTCTATACACTCAGTTGGAA 8440
CACGAGACCTGTCCAGGTTAAGCACCATTTTATCGCCCTT 8480
ATACAATACTGTCGCTCCAGGAGCAAACTGATGTCGTGAG 8520
CTTAAACTAGTTCTTGATGCAGATGACGTTTTAAGCACAG 8560
AAGTTAAAAGAGTGATAACTTCTTCAGCTTCAAATATCAC 8600

        8610      8620      8630      8640

CCCAGCTTTTTTCTGCTCATGAAGGTTAGATGCCTGCTGC 8640
TTAAGTAATTCCTCTTTATCTGTAAAGGCTTTTTGAAGTG 8680
CATCACCTGACCGGGCAGATAGTTCACCGGGGTGAGAAAA 8720
AAGAGCAACAACTGATTTAGGCAATTTGGCGGTGTTGATA 8760
CAGCGGGTAATAATCTTACGTGAAATATTTTCCGCATCAG 8800
```

8810 8820 8830 8840

CCAGCGCAGAAATATTTCCAGCAAATTCATTCTGCAATCG 8840
GCTTGCATAACGCTGACCACGTTCATAAGCACTTGTTGGG 8880
CGATAATCGTTACCCAATCTGGATAATGCAGCCATCTGCT 8920
CATCATCCAGCTCGCCAACCAGAACACGATAATCACTTTC 8960
GGTAAGTGCAGCAGCTTTACGACGGCGACTCCCATCGGCA 9000

9010 9020 9030 9040

ATTTCTATGACACCAGATACTCTTCGACCGAACGCCGGTG 9040
TCTGTTGACCAGTCAGTAGAAAAGAAGGGATGAGATCATC 9080
CAGTGCGTCCTCAGTAAGCAGCTCCTGGTCACGTTCATTA 9120
CCTGACCATACCCGAGAGGTCTTCTCAACACTATCACCCC 9160
GGAGCACTTCAAGAGTAAACTTCACATCCCGACCACATAC 9200

9210 9220 9230 9240

AGGCAAAGTAATGGCATTACCGCGAGCCATTACTCCTACG 9240
CGCGCAATTAACGAATCCACCATCGGGGCAGCTGGTGTCG 9280
ATAACGAAGTATCTTCAACCGGTTGAGTATTGAGCGTATG 9320
TTTTGGAATAACAGGCGCACGCTTCATTATCTAATCTCCC 9360
AGCGTGGTTTAATCAGACGATCGAAAATTTCATTGCAGAC 9400

9410 9420 9430 9440

AGGTTCCCAAATAGAAAGAGCATTTCTCCAGGCACCAGTT 9440
GAAGAGCGTTGATCAATGGCCTGTTCAAAAACAGTTCTCA 9480
TCCGGATCTGACCTTTACCAACTTCATCCGTTTCACGTAC 9520
AACATTTTTTAGAACCATGCTTCCCCAGGCATCCCGAATT 9560
TGCTCCTCCATCCACGGGGACTGAGAGCCATTACTATTGC 9600

9610 9620 9630 9640

TGTATTTGGTAAGCAAAATACGTACATCAGGCTCGAACCC 9640
TTTAAGATCAACGTTCTTGAGCAGATCACGAAGCATATCG 9680
AAAAACTGCAGTGCGGAGGTGTAGTCAAACAACTCAGCAG 9720
GCGTGGGAACAATCAGCACATCAGCAGCACATACGACATT 9760
AATCGTGCCGATACCCAGGTTAGGCGCGCTGTCAATAACT 9800

9810 9820 9830 9840

ATGACATCATAGTCATGAGCAACAGTTTCAATGGCCAGTC 9840
GGAGCATCAGGTGTGGATCGGTGGGCAGTTTACCTTCATC 9880
AAATTTGCCCATTAACTCAGTTTCAATACGGTGCAGAGCC 9920
AGACAGGAAGGAATAATGTCAAGCCCCGGCCAGCAAGTGG 9960
GCTTTATTGCATAAGTGACATCGTCCTTTTCCCCAAGATA 10000

10010 10020 10030 10040

GAAAGGCAGGAGAGTGTCTTCTGCATGAATATGAAGATCT 10040
GGTACCCATCCGTGATACATTGAGGCTGTTCCCTGGGGGT 10080
CGTTACCTTCCACGAGCAAAACACGTAGCCCCTTCAGAGC 10120
CAGATCCTGAGCAAGATGAACAGAAACTGAGGTTTTGTAA 10160
ACGCCTCCTTTATGGGCAGCAACCCCGATCACCGGTGGAA 10200

```
        10210     10220     10230     10240
ATACGTCTTCAGCACGTCGCAATCGCGTACCAAACACATC 10240
ACGCATATGATTAATTTGTTCAATTGTATAACCAACACGT 10280
TGCTCAACCCGTCCTCGAATTTCCATATCCGGGTGCGGTA 10320
GTCGCCCTGCTTTCTCGGCATCTCTGATAGCCTGAGAAGA 10360
AACCCCAACTAAATCCGCTGCTTCACCTATTCTCCAGCGC 10400

        10410     10420     10430     10440
CGGGTTATTTTCCTCGCTTCCGGGCTGTCATCATTAAACT 10440
GTGCAATGGCGATAGCCTTCGTCATTTCATGACCAGCGTT 10480
TATGCACTGGTTAAGTGTTTCCATGAGTTTCATTCTGAAC 10520
ATCCTTTAATCATTGCTTTGCGTTTTTTTATTAAATCTTG 10560
CAATTTACTGCAAAGCAACAACAAAATCGCAAAGTCATCA 10600

        10610     10620     10630     10640
AAAAACCGCAAAGTTGTTTAAAATAAGAGCAACACTACAA 10640
AAGGAGATAAGAAGAGCACATACCTCAGTCACTTATTATC 10680
ACTAGCGCTCGCCGCAGCCGTGTAACCGAGCATAGCGAGC 10720
GAACTGGCGAGGAAGCAAAGAAGAACTGTTCTGTCAGATA 10760
GCTCTTACGCTCAGCGCAAGAAGAAATATCCACCGTGGGA 10800

        10810     10820     10830     10840
AAAACTCCAGGTAGAGGTACACACGCGGATAGCCAATTCA 10840
GAGTAATAAACTGTGATAATCAACCCTCATCAATGATGAC 10880
GAACTAACCCCCGATATCAGGTCACATGACGAAGGGAAAG 10920
AGAAGGAAATCAACTGTGACAAACTGCCCTCAAATTTGGC 10960
TTCCTTAAAAATTACAGTTCAAAAAGTATGAGAAAATCCA 11000

        11010     11020     11030     11040
TGCAGGCTGAAGGAAACAGCAAAACTGTGACAAATTACCC 11040
TCAGTAGGTCAGAACAAATGTGACGAACCACCCTCAAATC 11080
TGTGACAGATAACCCTCAGACTATCCTGTCGTCATGGAAG 11120
TGATATCGCGGAAGGAAAATACGATATGAGTCGTCTGGCG 11160
GCCTTTCTTTTTCTCAATGTATGAGAGGCGCATTGGAGTT 11200

        11210     11220     11230     11240
CTGCTGTTGATCTCATTAACACAGACCTGCAGGAAGCGGC 11240
GGCGGAAGTCAGGCATACGCTGGTAACTTTGAGGCAGCTG 11280
GTAACGCTCTATGATCCAGTCGATTTTCAGAGAGACGATG 11320
CCTGAGCCATCCGGCTTACGATACTGACACAGGGATTCGT 11360
ATAAACGCATGGCATACGGATTGGTGATTTCTTTTGTTTC 11400

        11410     11420     11430     11440
ACTAAGCCGAAACTGCGTAAACCGGTTCTGTAACCCGATA 11440
AAGAAGGGAATGAGATATGGGTTGATATGTACACTGTAAA 11480
GCCCTCTGGATGGACTGTGCGCACGTTTGATAAACCAAGG 11520
AAAAGATTCATAGCCTTTTTCATCGCCGGCATCCTCTTCA 11560
GGGCGATAAAAAACCACTTCCTTCCCCGCGAAACTCTTCA 11600
```

```
       11610     11620     11630     11640
ATGCCTGCCGTATATCCTTACTGGCTTCCGCAGAGGTCAA 11640
TCCGAATATTTCAGCATATTTAGCAACATGGATCTCGCAG 11680
ATACCGTCATGTTCCTGTAGGGTGCCATCAGATTTTCTGA 11720
TCTGGTCAACGAACAGATACAGCATACGTTTTTGATCCCG 11760
GGAGAGACTATATGCCGCCTCAGTGAGGTCGTTTGACTGG 11800
       11810     11820     11830     11840
ACGATTCGCGGGCTATTTTTACGTTTCTTGTGATTGATAA 11840
CCGCTGTTTCCGCCATGACAGATCCATGTGAAGTGTGACA 11880
AGTTTTTAGATTGTCACACTAAATAAAAAAGAGTCAATAA 11920
GCAGGGATAACTTTGTGAAAAAACAGCTT 11949
```

```
LOCUS       pSMARTBAC2_UPRT-DHFR_GRA1p_SwStrp-MucOnly-3HA
 15126 bp   22-JAN-2016
EMBL_ID     pSMARTBAC2_UPRT-DHFR_GRA1p_SwStrp-MucOnly-3HA
 15126 bp   01-OCT-2015
EMBL_ID     pSMARTBAC2_UPRT-DHFR_GRA1p_SwStrp-MucOnly-3HA
 15126 bp   31-AUG-2015
EMBL_ID     pSMARTBAC2_UPRT-DHFR_GRA1p_SwStrp-MucOnly-3HA
 15260 bp   25-AUG-2015
EMBL_ID     pSMARTBAC2_UPRT-DHFR_GRA1p_SwStrp-MucOnly-3HA
 15126 bp   25-AUG-2015
EMBL_ID     pSMARTBAC2_UPRT-DHFR_GRA1p_SwStrp-MucOnly-3HA
 15126 bp   12-SEP-2015
EMBL_ID     pSMARTBAC2_UPRT-DHFR_GRA1p_SwStrp-MucOnly-3HA
 15126 bp   08-OCT-2015
UNIMARK     pSMARTBAC2 UPRT-DHFR GRA1p SwStrp-MucOnly-3HA
 annotations
            pSMARTBAC2 UPRT-DHFR GRA1p SwStrp-MucOnly-3HA


     primer_bind     9368..9436
                     /label="signal peptide CST1"
                     /ugene_name="Misc.\ Feature"
                     /ugene_group="Primer\ Binding\ Site"
     primer          complement(9449..9482)
                     /label="in-fusion 5'R"
     Region          9452..9457
                     /label="cleavage site"
     misc_feature    complement(9499..9569)
                     /label="105-4 vecR"
                     /ugene_name="primer_bind"
                     /ugene_group="Misc.\ Feature"
     protein_bind    9500..9595
                     /mismatches=0
                     /label="TwinStrep"
     misc_feature    9550..9589
                     /label="105-4 MOf"
                     /ugene_name="primer_bind"
     misc_feature    9575..9614
                     /label="Strep2MucOnlyF"
                     /ugene_name="primer_bind"
     primer_bind     complement(9611..9628)
                     /label="Null Primer R"
```

```
     primer_bind     9611..9628
                     /mismatches=0
                     /label="MucSeqF"
     Region          9629..10417
                     /label="Muc"
     repeat_unit     9668..9707
                     /label="M1"
     repeat_unit     9708..9733
                     /label="M2"
     repeat_unit     9734..9763
                     /label="M3"
     repeat_unit     9764..9793
                     /label="M4"
     repeat_unit     9794..9823
                     /label="M5"
     repeat_unit     9824..9856
                     /label="M6"
     repeat_unit     9857..9886
                     /label="M7"
     repeat_unit     9887..9916
                     /label="M8"
     repeat_unit     9917..9946
                     /label="M9"
     repeat_unit     9947..9976
                     /label="+M10"
     repeat_unit     9977..10012
                     /label="M11"
     repeat_unit     10013..10042
                     /label="M12"
     repeat_unit     10043..10072
                     /label="M13"
     repeat_unit     10073..10105
                     /label="M14"
     repeat_unit     10106..10135
                     /label="M15"
     repeat_unit     10136..10165
                     /label="M16"
     repeat_unit     10166..10195
                     /label="M17"
     repeat_unit     10196..10222
                     /label="M18"
     repeat_unit     10223..10249
```

```
                     /label="M19"
     repeat_unit     10250..10270
                     /label="M20"
     primer_bind     10418..10435
                     /label="MucSeqR"
     primer_bind     complement(10466..10485)
                     /mismatches=0
                     /label="101-colR2MO"
     misc_feature    complement(10548..10577)
                     /label="105-4 MOr"
                     /ugene_name="primer_bind"
     misc_feature    10558..10583
                     /label="105-4 HAf"
                     /ugene_name="primer_bind"
     misc_feature    10568..10651
                     /label="3HA"
                     /ugene_name="protein_bind"
     misc_feature    10653..11066
                     /label="3' UTR HXGPRT"
                     /ugene_name="3'UTR"
     primer_bind     complement(10799..10819)
                     /mismatches=0
                     /label="LIC-tagging-seq-R"
     misc_feature    complement(11051..11076)
                     /label="105-4 HAr"
                     /ugene_name="primer_bind"
     misc_feature    11057..11096
                     /label="105-4 vecF"
                     /ugene_name="primer_bind"
     misc_feature    11057..11076
                     /ugene_name="Misc.\ Feature"
                     /ugene_group="3'UTR"
     3'UTR           complement(11067..11630)
                     /label="T2-3'UTR"
     primer_bind     complement(11360..11379)
                     /mismatches=0
                     /label="ColR84-3"
     primer_bind     complement(11610..11630)
                     /created_by="User"
                     /label="T2ko-3'F"
     primer_bind     complement(11622..11661)
                     /label="pUC19wUPRT-vecF"
```

```
     misc_feature    complement(11637..11670)
                     /mismatches=0
                     /ugene_name="loxP"
     primer_bind     11646..11685
                     /label="DHFR-loxP-R"
     CDS             complement(11671..13503)
                     /mismatches=0
     primer_bind     complement(13487..13518)
                     /mismatches=0
                     /label="DHFR-loxP-F"
     primer_bind     13499..13538
                     /mismatches=0
                     /label="TUBp-loxPr"
     misc_feature    complement(13519..13552)
                     /ugene_name="loxP"
     promoter        complement(13553..13871)
                     /label="alpha tubulin TUBA1 promoter"
     misc_difference complement(13679..13681)
                     /label="GTG->CTC"
     misc_difference complement(13722..13723)
                     /label="CC->GG"
     promoter        13878..14010
                     /label="SAG1 promoter"
     primer_bind     complement(14011..14030)
                     /mismatches=0
     primer_bind     complement(14011..14047)
                     /label="DHFR-r-83-3"
     primer_bind     14031..14051
                     /mismatches=0
                     /label="OpenF-UPRT3-83-3"
     misc_recomb     14031..15126
                     /mismatches=0
                     /label="UPRT3' Recomb"
     3'UTR           14044..14175
                     /label="UPRT 3'UTR"
     misc_feature    complement(14207..15126)
                     /mismatches=0
                     /ugene_name="verified_seq"
     primer_bind     complement(14273..14296)
                     /created_by="User"
                     /label="UPRT3' sequencing primer"
                     /note="Geneious type: primer_bind
```

```
_reverse"
     primer_bind     complement(15106..15126)
                     /created_by="User"
                     /label="UPRT3'R"
                     /note="Geneious type: primer_bind
_reverse"
ORIGIN
Features
      misc_feature       1..15126
      /comment="Molecule is created with Unipro UGENE v1.16"
      /ugene_name="source"
      source             6..7601
      /organism="Cloning vector pSMART BAC v2.0"
      /mol_type="other DNA"
      /db_xref="taxon:480178"
      /PCR_primers="fwd_name: SL1, fwd_seq:
      cagtccagttacgctggagtc, rev_name: NZ-SR4, rev_seq:
      ttgaccatgttggtatgattt"
      /PCR_primers="fwd_name: SP6 promoter, fwd_seq:
      tatttaggtgacactatagaa, rev_name: T7 promoter, rev_seq:
      taatacgactcactataggg"
      /label="source Cloning vector pSMART BAC v2.0"
      primer_bind        complement(63..83)
      /mismatches=0
      /label="SR4-seqR"
      rep_origin         5793..6009
      /note="ori2; oriS"
      /label="ori2; oriS rep origin"
      rep_origin         6085..6699
      /note="oriV"
      /label="oriV rep origin"
      primer_bind        7525..7545
      /mismatches=0
      /label="SL1-seqF"
      misc_feature       7571..8381
      /mismatches=0
      /ugene_name="verified_seq"
      primer_bind        7582..7629
      /created_by="User"
      /label="UPRT5'F w/ adapter"
      primer_bind        7608..7629
      /created_by="User"
      /label="UPRT5'F"
      misc_recomb        7608..8758
      /modified_by="User"
      /label="UPRT5'"
      5'UTR              8362..8729
      /Source="annotation"
      /isDeprecated=0
      /label="UPRT 5'UTR"
      misc_feature       8388..8565
      /mismatches=0
      /ugene_name="verified_seq"
```

misc_recomb	7608..8758
/modified_by="User"	
/label="UPRT5'"	
5'UTR	8362..8729
/Source="annotation"	
/isDeprecated=0	
/label="UPRT 5'UTR"	
misc_feature	8388..8565
/mismatches=0	
/ugene_name="verified_seq"	
primer_bind	8466..8488
/created_by="User"	
/label="UPRT5' seqencing primer"	
misc_feature	8571..8600
/mismatches=0	
/ugene_name="verified_seq"	
misc_feature	8712..8720
/mismatches=0	
/ugene_name="verified_seq"	
primer_bind	complement(8742..8758)
/mismatches=0	
/label="UPRT5'R-84"	
misc_feature	8744..8780
/mismatches=0	
/label="GRApF-84"	
/ugene_name="primer_bind"	
promoter	8759..9361
/created_by="User"	
/modified_by="User"	
/label="GRA1promoter"	
primer_bind	8793..8810
/label="GRA1p-colonyF"	
primer_bind	9294..9313
/label="GRA1pSeqF"	
primer_bind	complement(9337..9364)
/mismatches=0	
/label="GRApR-84"	
primer_bind	9344..9383
/mismatches=0	
/label="CST1-strepII-F"	
misc_difference	9348..9348
/label="deleted"	

```
      misc_difference     9350..9350
      /label="deteted"
      CDS                 9362..10651
      /mismatches=0
      misc_feature        9367..10708
      /mismatches=0
      /ugene_name="verified_seq"

        10        20        30        40
TTAACAATTCACGTGACTTGAAGTCGCGGCCGCACTGACC 40
CTATAGTGAGTCGTATTAATTTAAATCATACCAACATGGT 80
CAAATAAAACGAAAGGCTCAGTCGAAAGACTGGGCCTTTC 120
GTTTTAATCTGATCGGCACGTAAGAGGTTCCAACTTTCAC 160
CATAATGAAATAAGATCACTACCGGGCGTATTTTTTGAGT 200
       210       220       230       240
TATCGAGATTTTCAGGAGCTAAGGAAGCTAAAATGGAGAA 240
AAAAATCACTGGATATACCACCGTTGATATATCCCAATGG 280
CATCGTAAAGAACATTTTGAGGCATTTCAGTCAGTTGCTC 320
AATGTACCTATAACCAGACCGTTCAGCTGGATATTACGGC 360
CTTTTTAAAGACCGTAAAGAAAAATAAGCACAAGTTTTAT 400
       410       420       430       440
CCGGCCTTTATTCACATTCTTGCCCGCCTGATGAATGCTC 440
ATCCGGAATTTCGTATGGCAATGAAAGACGGTGAGCTGGT 480
GATATGGGATAGTGTTCACCCTTGTTACACCGTTTTCCAT 520
GAGCAAACTGAAACGTTTTCATCGCTCTGGAGTGAATACC 560
ACGACGATTTCCGGCAGTTTCTACACATATATTCGCAAGA 600
       610       620       630       640
TGTGGCGTGTTACGGTGAAAACCTGGCCTATTTCCCTAAA 640
GGGTTTATTGAGAATATGTTTTTCGTCTCAGCCAATCCCT 680
GGGTGAGTTTCACCAGTTTTGATTTAAACGTGGCCAATAT 720
GGACAACTTCTTCGCCCCCGTTTTCACCATGGGCAAATAT 760
TATACGCAAGGCGACAAGGTGCTGATGCCGCTGGCGATTC 800
       810       820       830       840
AGGTTCATCATGCCGTTTGTGATGGCTTCCATGTCGGCAG 840
AATGCTTAATGAATTACAACAGTACTGCGATGAGTGGCAG 880
GGCGGGGCGTAACCTAGGTGACAGAAGTCAAAAGCCTCCG 920
GTCGGAGGCTTTTGACTTTCTGCTAGATCGGCCGCATCGA 960
ATATAACTTCGTATAATGTATGCTATACGAAGTTATTAGC 1000
       1010      1020      1030      1040
GATGAGCTCGGACTTCCATTGTTCATTCCACGGACAAAAA 1040
CAGAGAAAGGAAACGACAGAGGCCAAAAAGCTCGCTTTCA 1080
GCACCTGTCGTTTCCTTTCTTTTCAGAGGGTATTTTAAAT 1120
AAAAACATTAAGTTATGACGAAGAAGAACGGAAACGCCTT 1160
AAACCGGAAAATTTTCATAAATAGCGAAAACCCGCGAGGT 1200
```

```
        1210       1220       1230       1240
```

CGCCGCCCCGTAACCTGTCGGATCACCGGAAAGGACCCGT 1240
AAAGTGATAATGATTATCATCTACATATCACAACGTGCGT 1280
GGAGGCCATCAAACCACGTCAAATAATCAATTATGACGCA 1320
GGTATCGTATTAATTGATCTGCATCAACTTAACGTAAAAG 1360
CAACTTCAGACAATACAAATCAGCGACACTGAATACGGGG 1400

```
        1410       1420       1430       1440
```

CAACCTCATGTCCGAGCTCGCGAGCTCGTCGACAGCGACA 1440
CACTTGCATCGGATGCAGCCCGGTTAACGTGCCGGCACGG 1480
CCTGGGTAACCAGGTATTTGTCCACATAACCGTGCGCAA 1520
AATGTTGTGGATAAGCAGGACACAGCAGCAATCCACAGCA 1560
GGCATACAACCGCACACCGAGGTTACTCCGTTCTACAGGT 1600

```
        1610       1620       1630       1640
```

TACGACGACCATGTCAATACTTGCCCTTGACAGGCATTGA 1640
TGGAATCGTAGTCTCACGCTGATAGTCTGATCGACAATAC 1680
AAGTGGGACCGTGGTCCCAGACCGATAATCAGACCGACAA 1720
CACGAGTGGGATCGTGGTCCCAGACTAATAATCAGACCGA 1760
CGATACGAGTGGGACCGTGGTCCCAGACTAATAATCAGAC 1800

```
        1810       1820       1830       1840
```

CGACGATACGAGTGGGACCGTGGTTCCAGACTAATAATCA 1840
GACCGACGATACGAGTGGGACCGTGGTCCCAGACTAATAA 1880
TCAGACCGACGATACGAGTGGGACCATGGTCCCAGACTAA 1920
TAATCAGACCGACGATACGAGTGGGACCGTGGTCCCAGTC 1960
TGATTATCAGACCGACGATACGAGTGGGACCGTGGTCCCA 2000

```
        2010       2020       2030       2040
```

GACTAATAATCAGACCGACGATACGAGTGGGACCGTGGTC 2040
CCAGACTAATAATCAGACCGACGATACGAGTGGGACCGTG 2080
GTCCCAGTCTGATTATCAGACCGACGATACAAGTGGAACA 2120
GTGGGCCCAGAGAGAATATTCAGGCCAGTTATGCTTTCTG 2160
GCCTGTAACAAAGGACATTAAGTAAAGACAGATAAACGTA 2200

```
        2210       2220       2230       2240
```

GACTAAAACGTGGTCGCATCAGGGTGCTGGCTTTTCAAGT 2240
TCCTTAAGAATGGCCTCAATTTTCTCTATACACTCAGTTG 2280
GAACACGAGACCTGTCCAGGTTAAGCACCATTTTATCGCC 2320
CTTATACAATACTGTCGCTCCAGGAGCAAACTGATGTCGT 2360
GAGCTTAAACTAGTTCTTGATGCAGATGACGTTTTAAGCA 2400

```
        2410       2420       2430       2440
```

CAGAAGTTAAAAGAGTGATAACTTCTTCAGCTTCAAATAT 2440
CACCCCAGCTTTTTTCTGCTCATGAAGGTTAGATGCCTGC 2480
TGCTTAAGTAATTCCTCTTTATCTGTAAAGGCTTTTTGAA 2520
GTGCATCACCTGACCGGGCAGATAGTTCACCGGGGTGAGA 2560
AAAAAGAGCAACAACTGATTTAGGCAATTTGGCGGTGTTG 2600

```
        2610      2620      2630      2640
....|....|....|....|....|....|....|....|

ATACAGCGGGTAATAATCTTACGTGAAATATTTTCCGCAT 2640
CAGCCAGCGCAGAAATATTTCCAGCAAATTCATTCTGCAA 2680
TCGGCTTGCATAACGCTGACCACGTTCATAAGCACTTGTT 2720
GGGCGATAATCGTTACCCAATCTGGATAATGCAGCCATCT 2760
GCTCATCATCCAGCTCGCCAACCAGAACACGATAATCACT 2800

        2810      2820      2830      2840
....|....|....|....|....|....|....|....|

TTCGGTAAGTGCAGCAGCTTTACGACGGCGACTCCCATCG 2840
GCAATTTCTATGACACCAGATACTCTTCGACCGAACGCCG 2880
GTGTCTGTTGACCAGTCAGTAGAAAAGAAGGGATGAGATC 2920
ATCCAGTGCGTCCTCAGTAAGCAGCTCCTGGTCACGTTCA 2960
TTACCTGACCATACCCGAGAGGTCTTCTCAACACTATCAC 3000

        3010      3020      3030      3040
....|....|....|....|....|....|....|....|

CCCGGAGCACTTCAAGAGTAAACTTCACATCCCGACCACA 3040
TACAGGCAAAGTAATGGCATTACCGCGAGCCATTACTCCT 3080
ACGCGCGCAATTAACGAATCCACCATCGGGGCAGCTGGTG 3120
TCGATAACGAAGTATCTTCAACCGGTTGAGTATTGAGCGT 3160
ATGTTTTGGAATAACAGGCGCACGCTTCATTATCTAATCT 3200

        3210      3220      3230      3240
....|....|....|....|....|....|....|....|

CCCAGCGTGGTTTAATCAGACGATCGAAAATTTCATTGCA 3240
GACAGGTTCCCAAATAGAAAGAGCATTTCTCCAGGCACCA 3280
GTTGAAGAGCGTTGATCAATGGCCTGTTCAAAAACAGTTC 3320
TCATCCGGATCTGACCTTTACCAACTTCATCCGTTTCACG 3360
TACAACATTTTTTAGAACCATGCTTCCCCAGGCATCCCGA 3400

        3410      3420      3430      3440
....|....|....|....|....|....|....|....|

ATTTGCTCCTCCATCCACGGGGACTGAGAGCCATTACTAT 3440
TGCTGTATTTGGTAAGCAAAATACGTACATCAGGCTCGAA 3480
CCCTTTAAGATCAACGTTCTTGAGCAGATCACGAAGCATA 3520
TCGAAAAACTGCAGTGCGGAGGTGTAGTCAAACAACTCAG 3560
CAGGCGTGGGAACAATCAGCACATCAGCAGCACATACGAC 3600

        3610      3620      3630      3640
....|....|....|....|....|....|....|....|

ATTAATCGTGCCGATACCCAGGTTAGGCGCGCTGTCAATA 3640
ACTATGACATCATAGTCATGAGCAACAGTTTCAATGGCCA 3680
GTCGGAGCATCAGGTGTGGATCGGTGGGCAGTTTACCTTC 3720
ATCAAATTTGCCCATTAACTCAGTTTCAATACGGTGCAGA 3760
GCCAGACAGGAAGGAATAATGTCAAGCCCCGGCCAGCAAG 3800

        3810      3820      3830      3840
....|....|....|....|....|....|....|....|

TGGGCTTTATTGCATAAGTGACATCGTCCTTTTCCCCAAG 3840
ATAGAAAGGCAGGAGAGTGTCTTCTGCATGAATATGAAGA 3880
TCTGGTACCCATCCGTGATACATTGAGGCTGTTCCCTGGG 3920
GGTCGTTACCTTCCACGAGCAAAACACGTAGCCCCTTCAG 3960
AGCCAGATCCTGAGCAAGATGAACAGAAACTGAGGTTTTG 4000
```

```
        4010       4020       4030       4040
TAAACGCCTCCTTTATGGGCAGCAACCCCGATCACCGGTG 4040
GAAATACGTCTTCAGCACGTCGCAATCGCGTACCAAACAC 4080
ATCACGCATATGATTAATTTGTTCAATTGTATAACCAACA 4120
CGTTGCTCAACCCGTCCTCGAATTTCCATATCCGGGTGCG 4160
GTAGTCGCCCTGCTTTCTCGGCATCTCTGATAGCCTGAGA 4200
        4210       4220       4230       4240
AGAAACCCCAACTAAATCCGCTGCTTCACCTATTCTCCAG 4240
CGCCGGGTTATTTTCCTCGCTTCCGGGCTGTCATCATTAA 4280
ACTGTGCAATGGCGATAGCCTTCGTCATTTCATGACCAGC 4320
GTTTATGCACTGGTTAAGTGTTTCCATGAGTTTCATTCTG 4360
AACATCCTTTAATCATTGCTTTGCGTTTTTTTATTAAATC 4400
        4410       4420       4430       4440
TTGCAATTTACTGCAAAGCAACAACAAAATCGCAAAGTCA 4440
TCAAAAAACCGCAAAGTTGTTTAAAATAAGAGCAACACTA 4480
CAAAAGGAGATAAGAAGAGCACATACCTCAGTCACTTATT 4520
ATCACTAGCGCTCGCCGCAGCCGTGTAACCGAGCATAGCG 4560
AGCGAACTGGCGAGGAAGCAAAGAAGAACTGTTCTGTCAG 4600
        4610       4620       4630       4640
ATAGCTCTTACGCTCAGCGCAAGAAGAAATATCCACCGTG 4640
GGAAAAACTCCAGGTAGAGGTACACACGCGGATAGCCAAT 4680
TCAGAGTAATAAACTGTGATAATCAACCCTCATCAATGAT 4720
GACGAACTAACCCCCGATATCAGGTCACATGACGAAGGGA 4760
AAGAGAAGGAAATCAACTGTGACAAACTGCCCTCAAATTT 4800
        4810       4820       4830       4840
GGCTTCCTTAAAAATTACAGTTCAAAAAGTATGAGAAAAT 4840
CCATGCAGGCTGAAGGAAACAGCAAAACTGTGACAAATTA 4880
CCCTCAGTAGGTCAGAACAAATGTGACGAACCACCCTCAA 4920
ATCTGTGACAGATAACCCTCAGACTATCCTGTCGTCATGG 4960
AAGTGATATCGCGGAAGGAAAATACGATATGAGTCGTCTG 5000
        5010       5020       5030       5040
GCGGCCTTTCTTTTTCTCAATGTATGAGAGGCGCATTGGA 5040
GTTCTGCTGTTGATCTCATTAACACAGACCTGCAGGAAGC 5080
GGCGGCGGAAGTCAGGCATACGCTGGTAACTTTGAGGCAG 5120
CTGGTAACGCTCTATGATCCAGTCGATTTTCAGAGAGACG 5160
ATGCCTGAGCCATCCGGCTTACGATACTGACACAGGGATT 5200
        5210       5220       5230       5240
CGTATAAACGCATGGCATACGGATTGGTGATTTCTTTTGT 5240
TTCACTAAGCCGAAACTGCGTAAACCGGTTCTGTAACCCG 5280
ATAAAGAAGGGAATGAGATATGGGTTGATATGTACACTGT 5320
AAAGCCCTCTGGATGGACTGTGCGCACGTTTGATAAACCA 5360
AGGAAAAGATTCATAGCCTTTTTCATCGCCGGCATCCTCT 5400
```

```
         5410      5420      5430      5440
TCAGGGCGATAAAAACCACTTCCTTCCCCGCGAAACTCT 5440
TCAATGCCTGCCGTATATCCTTACTGGCTTCCGCAGAGGT 5480
CAATCCGAATATTTCAGCATATTTAGCAACATGGATCTCG 5520
CAGATACCGTCATGTTCCTGTAGGGTGCCATCAGATTTTC 5560
TGATCTGGTCAACGAACAGATACAGCATACGTTTTTGATC 5600
         5610      5620      5630      5640
CCGGGAGAGACTATATGCCGCCTCAGTGAGGTCGTTTGAC 5640
TGGACGATTCGCGGGCTATTTTTACGTTTCTTGTGATTGA 5680
TAACCGCTGTTTCCGCCATGACAGATCCATGTGAAGTGTG 5720
ACAAGTTTTTAGATTGTCACACTAAATAAAAAAGAGTCAA 5760
TAAGCAGGGATAACTTTGTGAAAAAACAGCTTCTTCTGAG 5800
         5810      5820      5830      5840
GGCAATTTGTCACAGGGTTAAGGGCAATTTGTCACAGACA 5840
GGACTGTCATTTGAGGGTGATTTGTCACACTGAAAGGGCA 5880
ATTTGTCACAACACCTTCTCTAGAACCAGCATGGATAAAG 5920
GCCTACAAGGCGCTCTAAAAAAGAAGATCTAAAAACTATA 5960
AAAAAAATAATTATAAAAATATCCCCGTGGATAAGTGGAT 6000
         6010      6020      6030      6040
AACCCCAAGGGAAGTTTTTTCAGGCATCGTGTGTAAGCAG 6040
AATATATAAGTGCTGTTCCCTGGTGCTTCCTCGCTCACTC 6080
GACCGGGAGGGTTCGAGAAGGGGGGGCACCCCCCTTCGGC 6120
GTGCGCGGTCACGCGCACAGGGCGCAGCCCTGGTTAAAAA 6160
CAAGGTTTATAAATATTGGTTTAAAAGCAGGTTAAAAGAC 6200
         6210      6220      6230      6240
AGGTTAGCGGTGGCCGAAAAACGGGCGGAAACCCTTGCAA 6240
ATGCTGGATTTTCTGCCTGTGGACAGCCCCTCAAATGTCA 6280
ATAGGTGCGCCCCTCATCTGTCAGCACTCTGCCCCTCAAG 6320
TGTCAAGGATCGCGCCCCTCATCTGTCAGTAGTCGCGCCC 6360
CTCAAGTGTCAATACCGCAGGGCACTTATCCCCAGGCTTG 6400
         6410      6420      6430      6440
TCCACATCATCTGTGGGAAACTCGCGTAAAATCAGGCGTT 6440
TTCGCCGATTTGCGAGGCTGGCCAGCTCCACGTCGCCGGC 6480
CGAAATCGAGCCTGCCCCTCATCTGTCAACGCCGCGCCGG 6520
GTGAGTCGGCCCCTCAAGTGTCAACGTCCGCCCCTCATCT 6560
GTCAGTGAGGGCCAAGTTTTCCGCGAGGTATCCACAACGC 6600
         6610      6620      6630      6640
CGGCGGCCGGCCGCGGTGTCTCGCACACGGCTTCGACGGC 6640
GTTTCTGGCGCGTTTGCAGGGCCATAGACGGCCGCCAGCC 6680
CAGCGGCGAGGGCAACCAGCCGAGGGCTTCGCCCTGTCGC 6720
TCGACTGCGGCGAGCACTACTGGCTGTAAAAGGACAGACC 6760
ACATCATGGTTCTGTGTTCATTAGGTTGTTCTGTCCATTG 6800
```

```
        6810      6820      6830      6840
CTGACATAATCCGCTCCACTTCAACGTAACACCGCACGAA 6840
GATTTCTATTGTTCCTGAAGGCATATTCAAATCGTTTTCG 6880
TTACCGCTTGCAGGCATCATGACAGAACACTACTTCCTAT 6920
AAACGCTACACAGGCTCCTGAGATTAATAATGCGGATCTC 6960
TACGATAATGGGAGATTTTCCCGACTGTTTCGTTCGCTTC 7000
        7010      7020      7030      7040
TCAGTGGATAACAGCCAGCTTCTCTGTTTAACAGACAAAA 7040
ACAGCATATCCACTCAGTTCCACATTTCCATATAAAGGCC 7080
AAGGCATTTATTCTCAGGATAATTGTTTCAGCATCGCAAC 7120
CGCATCAGACTCCGGCATCGCAAACTGCACCCGGTGCCGG 7160
GCAGCCACATCCAGCGCAAAAACCTTCGTGTAGACTTCCG 7200
        7210      7220      7230      7240
TTGAACTGATGGACTTATGTCCCATCAGGCTTTGCAGAAC 7240
TTTCAGCGGTATACCGGCATACAGCATGTGCATCGCATAG 7280
GAATGGCGGAACGTATGTGGTGTGACCGGAACAGAGAACG 7320
TCACACCGTCAGCAGCAGCGGCGGCAACCGCCTCCCCAAT 7360
CCAGGTCCTGACCGTTCTGTCCGTCACTTCCCAGATCCGC 7400
        7410      7420      7430      7440
GCTTTCTCTGTCCTTCCTGTGCGACGGTTACGCCGCTCCA 7440
TGAGCTTATCGGGTCCACTCGCGAACGCAGAAAGGCCCAC 7480
CCGAAGGTGAGCCAGTGTGATTACATTTGCGGCCTAACTG 7520
TGGCCAGTCCAGTTACGCTGGAGTCACTAGTATTTAGGTG 7560
ACACTATAGAAGCGGCCGCGACAACTTGTCCACGTGGAAT 7600
        7610      7620      7630      7640
TGTTAACGTGAGCTCATGCTGGAGCTTCGTATGGATAGGG 7640
TTACCCTGGTACACTCGGAAATGTTCTTTGAACGCGGCTG 7680
GTGTGGCTTCTTGTCCCTGGGTGTTTCCTCTCCTCCTTCG 7720
ACACGTGTTTTTTTCTGCTTTTTCCTGCGCCACCCGCTGT 7760
GCCTAGTATCGAAAGCTGTAGAGTTGCCACCCTGATCTTC 7800
        7810      7820      7830      7840
TCCCTTCCAACTCTCCGCTTCGACACACCCCCCAACGACT 7840
TGTTGTGAAGCTCTGGCCCCCCTGTTTCGGTTGTGACAGG 7880
CTCTCCAGGCGCTCTTGCACTCTCCAGTCCAGCTGGTGTC 7920
ATAGAACCGTTATCTCTAGAGTGCCACGAAAGAGCCCCCT 7960
CCTTTTATTCGTTGTTTCCCCTGGGTACGAGCTATCTTGT 8000
        8010      8020      8030      8040
GGCTTTCTTTGCTTGGGGATGACCCTTGTCGTGACCTTCT 8040
ATGCGTTCTCGATGAGAAACCGTCGACTCTTCGTTGCATG 8080
CAGGGGTTTCTGCGCGAAGATACTCTCCCACAGAGCCACA 8120
GAAAGTGGTAGCCGCTGTTTTCCAACGCAGCGTACGTACC 8160
TAGAGATCCTTCCAGGAAACGCTGAGCCTCCGCTGTGACT 8200
```

```
        8210      8220      8230      8240

GATTTTCTGCACGTTGGCACGTGGATATCGCGATCCCCTG 8240
TCCCCTCCACTGCATGCAGTGTGGCGGTAAGACGAGGGCC 8280
ATATCGATCCCATGGCAGCTGCTGTGTGGGGTGCCGCCAT 8320
ACGTAGGGGTACGATTTCTGGTCCTGCCCAACTTCCCGCC 8360
TGGTTGATTCGAGCAACTTACAAGGACAAGGCAATTAACC 8400

        8410      8420      8430      8440

ATCTTGCTATTTTTCCTGTTTTCGTCGTCATCGGCTGTCC 8440
TAGCTCTCACGGCTACCCATCGTTTTCCCAAGCGCTGTCA 8480
AGTGATGCCATTGGTTACACTGGACAAAGGCCCTGCAAAT 8520
ACGCGTGCCTCCATCATCGATCCCCCTTTATCTACTTTCT 8560
CGCTTTCCGTTATCCCTTCTGTCTGCTTTTTCTTTTCTCG 8600

        8610      8620      8630      8640

GCTCCATCTCATTCCCCTCGTTCCTTGTAGCGGCCCCGGC 8640
TTCTACGTTCCGCCTGCGATTCCCGTATTGGTCACGCCTC 8680
CACAAGGCGTATTCCTTTTTCGTCGGACCTTTCCACAGG 8720
GCTTCTAAAATGGCGCAGGTCCCAGCGAGCGGAAAGCTCG 8760
AAGGCTGTAGTACTGGTGCTCGTATGCGACACGCGGATTC 8800

        8810      8820      8830      8840

ACGGCGTATGCGACAAGCGGGATTGCTGATGAGCGTCGTT 8840
CCCCACTGAAGCGCGCGGAGTGGTGGGCGACCACCGGTCA 8880
CCTGGCGTCTCAGCTGCGCGAGGCACCCCTCTCATAGCGT 8920
GGTACTCGTCACGAATACCAATCGCTGGGGTCGCGGCGGG 8960
AGGAATATGCTGTTTGTTGACCATACGATCACGCTGAACG 9000

        9010      9020      9030      9040

AAAACATGGTATGAGACGCCGTAAGCGGGCACAGGTTGTT 9040
TGCCCTCGTCTCATTGCGGACCAATTCCCGGTCCACCGCT 9080
GCGTCTCGACTCGACGGTTGTGACCACCCCACTTCGCATT 9120
GGGCAGTCGGTAAAGCCACAACATTACTTTGCAATTTTAT 9160
CGGTTGAAACTGCCGAGCGAGCTTGCGTTTTTGGGTGCTA 9200

        9210      9220      9230      9240

TCTTCTCCCACCTTTTATCAGTTAAGTTGTACAGTGAGTG 9240
TCAGCTTGTTTCGACACGTCTGTATAGACGCAACTCGGTT 9280
TGCTTGTGTTGTTTGGTGGCTGGCCAAATCAAAGGCTATT 9320
CATTTTTCACTTGCTGTTGTTCTTTGAAGAAATCAAGCAA 9360
GATGAAATTATCTTATTTCTTTTTCGCGCTGTCAAGCAGT 9400

        9410      9420      9430      9440

GGAGCAACCCTGCTTTTATTCGCCGTTACGCAGCTAGCAA 9440
CCCAGGTGTCCGGGTTCGATCTGGAAGAACCCCGAGATCT 9480
GAGGCATGAGATGAAGAAATCGGCTTGGTCGCACCCGCAA 9520
TTTGAAAAAGGTGGAGGGTCGGGCGGCGGTTCGGGAGGTT 9560
CCGCATGGTCTCATCCTCAGTTCGAGAAGGGCGGTGGATG 9600
```

```
        9610      9620      9630      9640

CCGCCAACCAGACCCACTGCAACATGAAACGTGCACAGTA 9640
CGTGTAACCGTGTTGCCGCCTGCGGCGCAGCCGACAACTA 9680
CAACGACGACAACCACTACTACGACAATCAAGCCAACAAC 9720
GACTACTACAACCAGAAAACCTACCACAACAACAACCACA 9760
ACCAGGAGACCTACCACAACTACAACAACAACAAAGAAAC 9800

        9810      9820      9830      9840

CTACCACAACAACGACTACAACCAGGAAACCTAGAACAAC 9840
AACGACCACTACAACCAAAAAACCTACCACAACAACAACC 9880
ACAACCAGGAAACCTACCACAACAACAACTACAACCAAGA 9920
AACCTACCACAACAACAACTACAACCAAGAAACCTACCAC 9960
AACAACAACTACAACCAAGAAACCTACTACAACGACGACA 10000

       10010     10020     10030     10040

ACAACAACAACCAGGAAACCTACCACAACAACAACTACAA 10040
CCAAGAAACCTACCACAACAACAACTACAACCAAGAAACC 10080
TACCACAACAACGACAACAACAACCAAGAAACCTACTACA 10120
ACAACAACTACAACCAAGAAACCAACAACTACTACTACTA 10160
CAACCAGGAAACCTACCACAACAACAACTACGACCAGGAA 10200

       10210     10220     10230     10240

ACCAACAACAACTACTACAACCAACAAGCCTACCACAACG 10240
ACAACAACTAGAACACCTACCACAACAACATCGAAACCGC 10280
CTGTGGTTTTCGAGCTTCCTTCCGAATTTATCGAAGAAAC 10320
AGCCACAACAACCAGGGCGACCATCTCGCTGCGTGCGAGG 10360
TCAGCGGCAGCACCAACTTCAACTACTACAGTCGGACCAA 10400

       10410     10420     10430     10440

CAACACCTGCTACGTCACTCTTCGACTGGAGCAGTAAAGT 10440
AGCCTCCCTGGCAGGCCAGTGGAGCTTTTCAGGAGCTCTG 10480
CAGGGAACTGCCGGTTCCCGATCTGGTGTTTATGAAGCTC 10520
CAATCACTCAACTGCCCCCCCCTCTTTTGGTGCCTTTTAA 10560
GATCGACTACCCGTACGACGTCCCGGACTACGCTGGCTAT 10600

       10610     10620     10630     10640

CCCTATGATGTGCCCGATTATGCGTATCCTTACGATGTTC 10640
CAGATTATGCCTAACCCGGGCATATGTAGAAAAGTTGTAA 10680
CGTTAGTAAACGTAACACACGGGTATAATGCCATTTAATG 10720
GCGCCGTATATTTTTCACTCCCCCTCTCCGATAGGAGGAA 10760
AAAGACGATCCGTCTGCGAATGTATATTACATCGACCGGC 10800

       10810     10820     10830     10840

CATAGTACGAGAAGTGGTGCCGGTATTGTTCTGTCCGGGG 10840
GATGTACCATCCTATTTTTTGTATATGTCGTTCTTGTCGG 10880
GGAGCAACAGCCCATCTTCCGGCTATTGTCTCGTATCAGT 10920
TGTGAAACAGACTGCGTTTTGCCGAAAATGAGTCCTATGT 10960
CCGATGGCTATTTGTACCGCTTCGTACCATTTCGCTTGTC 11000
```

```
        11010     11020     11030     11040
AGCTGTAATCAACCGAATTCATTTGGCGAGACATGATTCC 11040
GATTAAATTCTTTACTGCGCGTGCTCTGTCTCTTAGTTCA 11080
AAAATCTGTTTTAAACGGCTGTTGATGGAACCCGGCTCGG 11120
CGCTTCGCCAGCACCAGCGAAAGCAGTATCGCTACACCAA 11160
AAGAAGAGACTTCCGACGACTGTGTCTTCAAACGGAAAAA 11200
        11210     11220     11230     11240
AGAGACGTTCTGCGCGCTGAGCCTCGATGTGAAAGAAAAA 11240
GTCAGTGTACACTATTTCCCCTCTGAGTCGAGGAGAAACC 11280
AAGACGCGGAATTTACACCGAGAGACACTCGGAGTTCCTC 11320
CGGTGTCTGTACATCGGACCATATGTATGTACACCTAAAT 11360
TTCTCGTGCCAGTTGGAGCGCCAGCCAACTTCGCGATTCT 11400
        11410     11420     11430     11440
GCTTTTTTTCGTTTATCTACACGCTCGTTTTTGTGGCGCC 11440
GGGTTCGTTTGTACAGCTCTTTGCAGTCTGCATTCGGCGA 11480
CGGGTGTGCTCTTCCGTGGATCGCCTTCTCCCCCGTCGCG 11520
TATAGCCGTTCTGCCTCCTGGGTGTCGCCGTTTTCCCCGT 11560
TTGGGGGGTTTCGTTGCGAACGCCGGGAGAACTGAAACGG 11600
        11610     11620     11630     11640
CCGCCTTGTGTGCCGTTTCTCGCTTACGCTGGTACCATAA 11640
CTTCGTATAATGTATGCTATACGAAGTTATCTAGACAGCC 11680
ATCTCCATCTGGATTCGTCCGTGCGGGACGTAGCCCACGA 11720
CCTCAAAATCCTCGGCGGTGAAATCGTCGATTTCCTTGAT 11760
GCGTTCCTTGTTGAGGATGTTCACAATGGGGAACGGTCTC 11800
        11810     11820     11830     11840
GGTTCTCTCCGCAGCTGCTCTTTTAAAGCCTCGACATGGT 11840
TCGTGTAGACATGCGTGTTCCCCATGAAGTGAATGAACTC 11880
CTTAGGTTTTAGGTTGCAGACGTGTGCAACCATGAGCGTC 11920
AAAAGCGAATAGGAAGCGATGTTGAAGGGGACGCCGAGGC 11960
CGACATCGCACGACCGCTGATACATGATGCACGACAGCTC 12000
        12010     12020     12030     12040
CTTCTGGTCGTTCACGTAGAACTGGCACAACAAGTGACAA 12040
GGCGGCAGCGCCATTTCGTCCAGCGCTGCAGGATTCCAGG 12080
CAGTCATGAGCATGCGACGATCTGTTGGATTCGTTCTCAG 12120
CATCTGGATCACATTCTTCAGCTGGTCGACGCCCTGCCCT 12160
GTGTAGTCTGTGTGCATGTCTTTGTATGCCGCGCCGAAGT 12200
        12210     12220     12230     12240
GTCTCCACTGGAAGCCGTAGCCCGGGCCGATGTCTCCGAC 12240
CTCTCGGTGGGGGAGATTGCGCGAATCGAGGAACTCGCGT 12280
GTCACATTCTTGTCCCAGATCTTCACGCCCTTCTCAGAAA 12320
GATGGTTTGCGTTCGTGTCGCCGCGAATGAACCACAGCAA 12360
CTCTTCGAGGACCCCTTTCCAGAACACACGCTTTGTGGTG 12400
```

```
        12410     12420     12430     12440
AGAAGTGGAAAGGCCTGATCCAGCGAGTAGCGCATAGTGC 12440
AGCCGAATTTGGAGATGACACCAACGCCCGTTCGGTCATC 12480
CATTGTCCTTCCATTGTTAATAATGTCGGCAATGAGATCA 12520
AGGTACTGGAATTCTTCATGGCCTCTAAAGTGAACATGCG 12560
GAACGGCCCGAATCAGTTCCTTTTGCTCGCGTTTTTTCCG 12600
        12610     12620     12630     12640
GTCTTCTTCGTCCATCCACGCCAACACCGGGGCAATGGCT 12640
GCGGCCGAAGAAGGAGCCTGCAACCCGTGCACGGGAGTTG 12680
TCTCCCTCGTGGACGTCAAGGAGCTCATTGCGTTGCTCGG 12720
TTCCGCAGTGGCTGCGTCGTCAGTCTTCCTTCTCTTCTCG 12760
AGAACCACGGAGTCGTAGGGTACCCCGTTGTCTGAGAAGG 12800
        12810     12820     12830     12840
TCTTGGAAATGAAGATGGGTCGATACGTCGCTTCATTGTC 12840
CTTCTCTCTTCCGAGCTCCGGACAAAAGGGAACGAACACA 12880
GACTCGGCAGGAGCTGCAGCCTGCGCAGCAGTTGATTTGT 12920
TTGAAAGAATGTCATCTCCGGGGAACGCAGGGAAGAAAAC 12960
GTCGCACGGAAACTCGCGGGCTACACGCGTGATGTACAGG 13000
        13010     13020     13030     13040
TGAGAGGCAACGCCCAGAGACAGCGCTGCCTCGTACAGTC 13040
CCGCTCCTCCCACGACAAAAATCTGGTCGACAGAATCCTT 13080
GTACTCTTCCTCCAGAAGGCTGAGAGCTGCTGGGAGTGAA 13120
GCACAGACTCGGACGCGCTGCTGGCCTTCAGCTTGAGGCT 13160
TCTCCGCCGCAATGTCTTCTTCTTTGAGGGAAGAGGAAAC 13200
        13210     13220     13230     13240
GACGATGTTCAATCTGTCCACGAGGGGTCTAAACTTTCGA 13240
GGCATGCTTTCCCAGTTTTTCCGTCCCATGACAACGGCGT 13280
TGAATCTCTTGCCGACTGATGGAGAGGGAAGTCCAGAGTC 13320
GCCCGTCTTTGCAAATTTCCTGGGAAGCCACCCGTTCAGG 13360
CGACTGGCTTCTTCGGGCGTCGTTTTTGTCACACGACGAA 13400
        13410     13420     13430     13440
AGTGTTTGAAATCTGTGGTCAAGTGGGGCCACGGGAGGCC 13440
GTTGTTGATGCCGATGCCCCTCTTGGGGGTCATCGCGACG 13480
ACCAGACACACCGGTTTCTGCATTTTTGTCGGAATTCTAT 13520
AACTTCGTATAATGTATGCTATACGAAGTTATAGAAAAAA 13560
TGCCAACGAGTAGTTTTCCGCAAGAACTTCACACAAGGTG 13600
        13610     13620     13630     13640
ATTGTGTAACACCGTTTGGTGGATGTCTTCTGCGCGGGTT 13640
GCGTCGTCAGGGACGGCGAACGAGTGCTTGTTCTCTACGA 13680
GACTCGCAAAGAGAAGGAAAGGGCGAAATTCAAGAGAGAA 13720
TCCAACAAGGAGGACTCTCTGCTAATATTCGCCGCCTTTA 13760
ACACATCTAAAAGTTCACAGAGAACAAACAAAATGTTTCT 13800
```

```
        13810     13820     13830     13840
GACACATCCCCTTCGTGCGCGCGCATCTCCACCGGTCCAA 13840
CCAGGATCACCAACCGCACCCAATGCAGGCAAGCTTAACC 13880
ACAAACCTTGAGACGCGTGTTCCAACCACGCACCCTGACA 13920
CGCGTGTTCCAACCACGCACCCTGAGACGCGTGTTCTAAC 13960
CACGCACCCTGAGACGCGTGTTCTAACCACGCACCCTGAG 14000
        14010     14020     14030     14040
ACGCGTGTTCAAGCTTATCGATACCGTCGATGGAACCATG 14040
TAGGTCTCTAGTTTTTTTGACAGACCGCTGACGGAATCGC 14080
GGACTGGAAGAAGCACGCCTCAAGAGGATAAACACCATTT 14120
TTATTTTTGTCTCGCTCCCACGTCCCTCACCATCGGCAGG 14160
AACACAGAAAAAAAGTTTTAGTTGTGAGGCTGCCTGGTGC 14200
        14210     14220     14230     14240
GCGACAAGCAGTTGCGCTTTTTTGTGCCACGGGGTATATC 14240
TTTGGTCGCGCTCCTGTCACGACTGTTGTTAGTCATGGAC 14280
CGGACGATTTCTTCGTATCAGGCACAGAGTCCCGTACACC 14320
TGTAACGGGAATGTTTACGAGGCGGCGTGCATCCCGTTCC 14360
TCGTCCATCTCGAGTTTTATTTCCCTTTCCTTTGTTCGAG 14400
        14410     14420     14430     14440
ATTCTGCTGTCCAGAACGAGACAGGGAAGCGAAGTGGTAG 14440
TGCCAGGAAGGGCGCAGCTTAATTGCTCCTCTTCACCTTA 14480
AGTCGTTTACTTGTGCCCAAGAAGTGTTCTTCACGATTAC 14520
CTTGTGTGTTTCCGCCGTTCTTTGCTTTTTGTTCGCGCTG 14560
CTCGGAAACATTTACAACCTCATATCTAGATGTTGTTGAA 14600
        14610     14620     14630     14640
GCAATGTGAAGAATGGTCCACGTTACATTCTCTAACGGAA 14640
TTTTAACGGTGTGGGATGGACAAGCAATGCATATGCGATG 14680
TCGAACCCTCGAAAACCCGTAATGTGCGACCACGTCCTGG 14720
GTGAAGCAAACGCGTTTCCACATACAGTCGGTATCGATGT 14760
CACGCTTGCCAGTTCGTGTAGGGAAGTTGGCGTGTTTTTT 14800
        14810     14820     14830     14840
TTTCCACGGTGGGGATTGAACAGTGTCATTGCCCAGACCA 14840
TGGCAGTACCAAGTCTGCTCTGATGAAGTGCGCCACCAGC 14880
ATTCTGTTTTTTCGCTCCCACGAGGGTGGAACCTCTAGCA 14920
CACAACTTCTCTCTTCTGAGGTTGCCGAAAAACTGTCTGC 14960
TAATTTATCGAGTTATTTGAACTACGGGACCAACGGGCAG 15000
        15010     15020     15030     15040
AGACTGTTTTCACTCGGATGTCGTACAGTTGACGTCGCGA 15040
CGAAAGGCACATGACCAAGGCGATTTTTTTTATTTTCTGG 15080
TTCATCCGGCTATCCACCAGTGTTGCTTCGGTCGATCGAA 15120
CGCCAG 15126
```
